# Efficacy of everolimus and letrozole with or without ribociclib in recurrent endometrial cancer: a phase 2 randomized clinical trial

**DOI:** 10.1038/s41467-026-77049-3

**Published:** 2026-09-01

**Authors:** Jeffrey A. How, Shannon N. Westin, Melissa Pham, Barrett Lawson, Minxing Chen, Nidhi Tandon, Ying Yuan, Rugang Zhang, Amir A. Jazaeri, Anil K. Sood, Michaela Onstad Grinsfelder, Kathleen M. Schmeler, David J. Vining, Samantha M. Fix, Yibo Dai, Mikayla B. Bowen, Karen H. Lu, Melinda S. Yates, Russell Broaddus, Pamela T. Soliman

**Affiliations:** 1 Department of Gynecologic Oncology and Reproductive Medicine, The University of Texas MD Anderson Cancer Center, Houston, TX USA; 2 Department of Anatomic Pathology, The University of Texas MD Anderson Cancer Center, Houston, TX USA; 3 Department of Biostatistics, The University of Texas MD Anderson Cancer Center, Houston, TX USA; 4 Department of Pathology and Laboratory Medicine, University of Texas McGovern Medical School, Houston, TX USA; 5 Department of Experimental Therapeutics, The University of Texas MD Anderson Cancer Center, Houston, TX USA; 6 Division of Diagnostic Imaging, The University of Texas MD Anderson Cancer Center, Houston, TX USA; 7 Department of Genomic Medicine, The University of Texas MD Anderson Cancer Center, Houston, TX USA; 8 Department of Pathology and Laboratory Medicine, University of North Carolina School of Medicine, Chapel Hill, NC USA

**Keywords:** Endometrial cancer, Targeted therapies, Tumour biomarkers

## Abstract

In this phase II trial, patients with endometrial cancer (EC) were randomized to everolimus/letrozole with (experimental arm) or without ribociclib (control arm) (ClinicalTrials.gov Identifier: NCT03008408). The primary endpoint was progression-free survival (PFS). Secondary endpoints included toxicity, overall survival (OS), objective response rate (ORR), and clinical benefit rate (CBR). For all-comers, there was no difference in PFS (HR 0.78, 95% CI 0.4-1.4, *p* = 0.38), OS (HR 0.8, 95% CI 0.4-1.5, *p* = 0.46), ORR (44.1% vs 25.7%, *p* = 0.11), or toxicity-related treatment discontinuation (4 [8.6%] vs 3 [11.4%], *p* = 0.22). Among those with prior endocrine therapy, ORR was higher in the experimental arm (61.5% vs 0.0%, *p* < 0.001). Within the experimental arm, *CTNNB1*_mut_ tumors were associated with improved PFS (HR 0.27, 95% CI 0.1-0.7, *p* = 0.006) and OS (HR 0.24, 95% CI 0.1-0.7, *p* = 0.004) compared to those with non-*CTNNB1*_mut_ tumors. Despite no survival improvement in all-comers, addition of ribociclib to everolimus/letrozole may improve efficacy in patients with prior endocrine therapy and cases with *CTNNB1*_mut_ tumors may have prolonged response.

## Abstract (web summary)

Therapeutic options for recurrent endometrial cancer (rEC) are limited. Here, the authors present a randomized phase 2 clinical trial comparing the combination of mTOR and aromatase inhibitors (everolimus and letrozole) with or without the CDK4/6 inhibitor, ribociclib, in patients with rEC.

## Introduction

Annual mortality for endometrial cancer (EC) has continued to rise over the past four decades, surpassing ovarian cancer as the most lethal gynecologic malignancy in the United States^1–3^. Management of advanced/recurrent EC is challenging with limited options following progression on first-line systemic therapy^4^. The predominant histologic subtype of EC is endometrioid (~80%) and often retains estrogen/progesterone receptor (ER/PR) expression, even in grade 3 endometrioid tumors^4,5^. Current endocrine therapeutic strategies are limited by modest, transient treatment responses. Thus, rational combinational strategies are needed to augment the efficacy of endocrine therapy.

The PI3K/AKT/mTOR pathway (activated in up to 90% of endometrioid EC) plays an important role in cellular growth and survival in EC with cross-talk with other signaling pathways (e.g., ER/PR signaling)^6–8^. Furthermore, 25% of patients with endometrioid EC will have a *CTNNB1* mutation resulting in WNT/β-catenin pathway activation and higher expression of downstream targets (e.g., cyclin D1, c-Myc)^6,9,10^. Cyclin D1 interacts with cyclin-dependent kinases (CDK4/6) to promote cell cycle progression from G1 to S phase. *CTNNB1* mutations are associated with worse progression-free survival (PFS) in endometrioid EC^6,10^.

Given the prevalence of PI3K/AKT/mTOR and WNT/β-catenin pathway alterations and their association with endocrine therapeutic resistance, there has been increasing interest in combining agents targeting these pathways^8,11,12^. Everolimus (mTOR inhibitor) plus letrozole (aromatase inhibitor) is an active regimen with an objective response rate (ORR) and clinical benefit rate (CBR) of 28–32% and 40–50%, respectively^13–15^. Additionally, CDK4/6 inhibitors have shown clinical activity (ORR 9–30% and CBR 35–64%) in combination with letrozole in ER-positive EC^16–18^. However, the feasibility of combining CDK4/6 inhibitors with everolimus plus letrozole and the potential to improve therapeutic efficacy in EC are unknown.

We conducted a phase II randomized controlled trial to evaluate the efficacy of the combination of everolimus and letrozole with or without ribociclib in advanced/recurrent EC. Our hypothesis was that the addition of ribociclib would improve therapeutic efficacy, particularly among patients with *CTNNB1* mutant (*CTNNB1*_mut_) tumors.

In this work, we show that there is significant, durable antitumor activity of everolimus and letrozole with or without ribociclib in advanced/recurrent endometrioid EC, but no statistical difference in efficacy between treatment arms in all comers. However, the addition of ribociclib to everolimus and letrozole may improve efficacy among those with prior endocrine therapy exposure and may prolong therapeutic response for multiple patients with tumors harboring *CTNNB1* mutations.

## Results

**Fig. 1:**
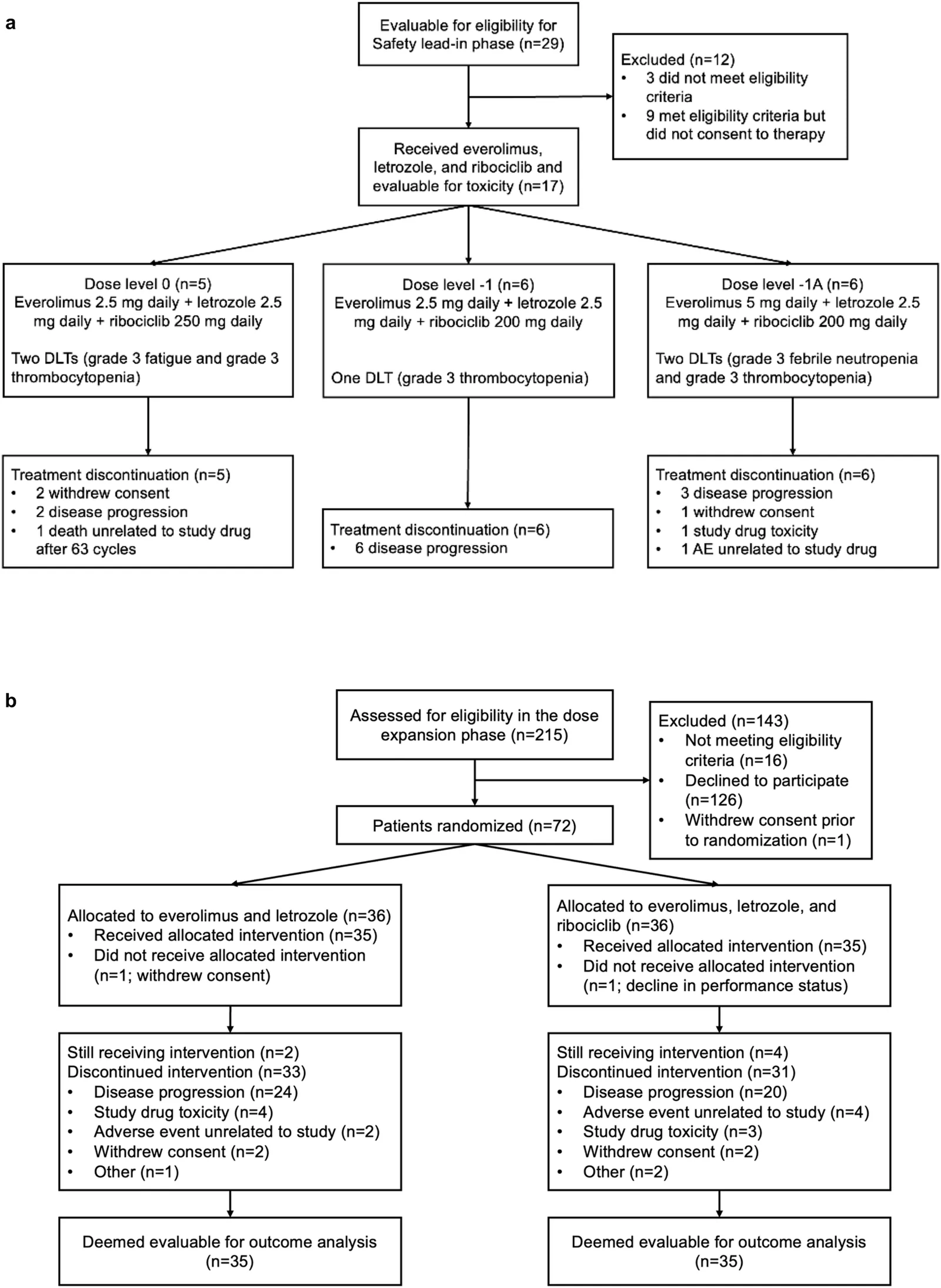
Flow diagram of patient enrollment and disposition. **a** Safety lead-in phase of patient enrollment and disposition. **b** Dose expansion randomization phase of patient enrollment and disposition.

**Fig. 2:**
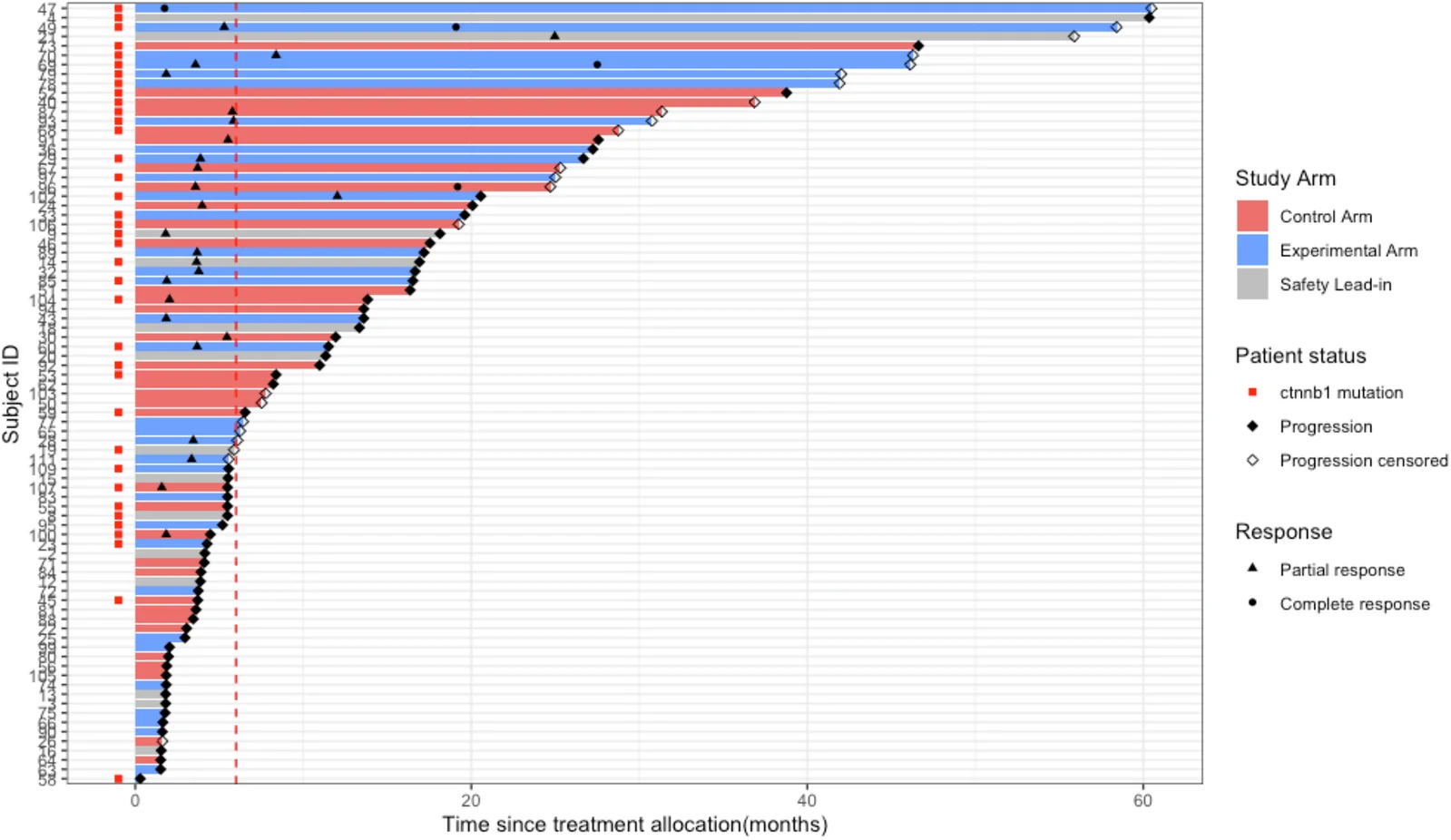
Swimmer plot demonstrating outcomes for evaluable patients in the study until disease progression. Three patients in the safety lead-in and one patient in the experimental group were not evaluable for antitumor response. The vertical dashed red line denotes 6 months of progression-free survival. Source data are provided as a Source Data file.

In the safety lead-in phase, 17 patients were enrolled (9/2017-11/2018) and treated with everolimus, letrozole, and ribociclib. Details of the dose selection for the experimental arm are demonstrated in Fig. 1a. There were two DLTs each in the DL 0 and −1A cohorts, and thus DL −1 was chosen. In the dose expansion phase, 72 patients were enrolled (3/2019-1/2024) and randomized (Fig. 1b). One patient in each arm received no study drug (Fig. 1b), leaving 35 patients per arm for safety and/or efficacy reporting. Baseline clinicopathologic characteristics were overall balanced in each arm (Table 1). For the overall study population (*n* = 87), 59% were Non-Hispanic White and 41% were Non-White and/or Hispanic. Approximately half had moderate-high/high SVI (48%) or ADI (55%) scores. 40% had grade 3 tumors, and 40% had prior endocrine therapy. Molecular profiling characteristics are shown in Supplementary Table 3. At the data cutoff date (June 14, 2025), the median follow-up was 17.8 months (range 1.1–86.4) in the safety lead-in arm, 19.3 months (range 3.4–65.2) in the control arm, and 26.9 months (range 2.9–68.9) in the experimental arm. Figure 2 presents a swimmer plot summarizing treatment duration and PFS outcomes for all patients.

**Table 1:** Clinicopathologic characteristics

	No. (%)		
		Randomized phase	
	Safety lead-in (*n* = 17)	Control Arm (*n* = 35)	Experimental Arm (*n* = 35)
Median age (range)	66 (53–75)	58 (40–79)	61 (36–79)
Race and ethnicity			
Non-Hispanic White	12 (71)	23 (66)	16 (46)
Non-Hispanic Black	1 (6)	1 (3)	3 (9)
Hispanic	1 (6)	9 (26)	12 (34)
Asian	2 (12)	2 (6)	3 (9)
Other	1 (6)	0 (0)	1 (3)
Social vulnerability index score^a^			
Low	3 (19)	9 (28)	10 (29)
Low-moderate	7 (44)	7 (22)	7 (20)
Moderate-high	3 (19)	8 (25)	8 (23)
High	3 (19)	8 (25)	10 (29)
Not evaluable	1	3	0
Area deprivation index score^a^			
Low	5 (31)	3 (9)	7 (20)
Low-moderate	1 (6)	11 (34)	10 (29)
Moderate-high	6 (38)	12 (38)	10 (29)
High	4 (25)	6 (19)	8 (23)
Not evaluable	1	3	0
Histology			
Endometrioid	16 (94)	32 (91)	31 (89)
Mixed	1 (6)	3 (9)	4 (11)
Grade			
1	0 (0)	3 (9)	4 (11)
2	7 (41)	19 (54)	19 (54)
3	10 (59)	13 (37)	12 (34)
*CTNNB1* status			
Wildtype	10 (59)	20 (57)	16 (49)
Mutant	7 (41)	15 (43)	17 (52)
Unknown	0	0	2
ECOG PS			
0	15 (88)	25 (71)	17 (49)
1	2 (12)	10 (29)	18 (51)
Prior pelvic radiation	8 (47)	18 (51.4%)	17 (48.6%)
Previous anticancer therapy type			
Chemotherapy	17 (100)	35 (100)	34 (97)
Endocrine therapy^b^	5 (29)	16 (46)	14 (40)
Aromatase inhibitor therapy	1 (6)	7 (20)	6 (17)
Progestin plus SERM	3 (18)	3 (9)	4 (11)
Hormonal receptor blocker	2 (12)	7 (20)	3 (9)
Progestin alone	0 (0)	1 (3)	1 (3)
Immunotherapy	1 (6)	12 (34)	7 (20)
Targeted therapy	5 (29)	6 (17)	7 (20)
Total number of prior systemic therapies in the frontline and recurrent setting			
Median (range)	2 (1–4)	2 (1–5)	2 (0–6)
0	0 (0)	0 (0)	1 (3)
1	6 (35)	17 (49)	16 (46)
2	8 (47)	13 (37)	10 (29)
≥3	3 (18)	5 (14)	8 (23)
Total number of prior systemic therapies in the recurrent setting			
Median (range)	1 (0–3)	1 (0–4)	1 (0–5)
0	4 (24)	5 (14)	10 (29)
1	5 (29)	19 (54)	13 (37)
2	5 (29)	7 (20)	6 (17)
≥3	3 (18)	4 (11)	6 (17)
Number of prior chemotherapy lines in the recurrent setting			
Median (range)	1 (0–2)	1 (0–2)	1 (0–2)

*ECOG PS* Eastern Cooperative Oncology Group Performance Status. Number (%) and median (range) are used as appropriate. *SERM* selective estrogen receptor modulator.

^a^Social Vulnerability Index (SVI) score is a composite measure of social vulnerability between 0 (least vulnerable) to 1 (most vulnerable) using 16 variables that correspond to social determinants of health^37^. Area Deprivation Index (ADI) is a census tract-level measure of socioeconomic disadvantage based upon factors for the theoretical domains of income, education, and employment, with an overall range from 0 (least disadvantaged) to 1 (most disadvantaged)^40,41^. SVI and ADI scores were categorized as low (<0.25), low-moderate (0.25–0.49), moderate-high (0.50–0.74), or high (0.75–1.0). Patients lacking a home address in their medical records or those with addresses for P.O. boxes instead of home addresses did not receive an SVI or ADI score and were excluded from analyses related to SVI and ADI.

^b^Patients may have had more than 1 regimen of endocrine therapy.

### Efficacy

**Fig. 3:**
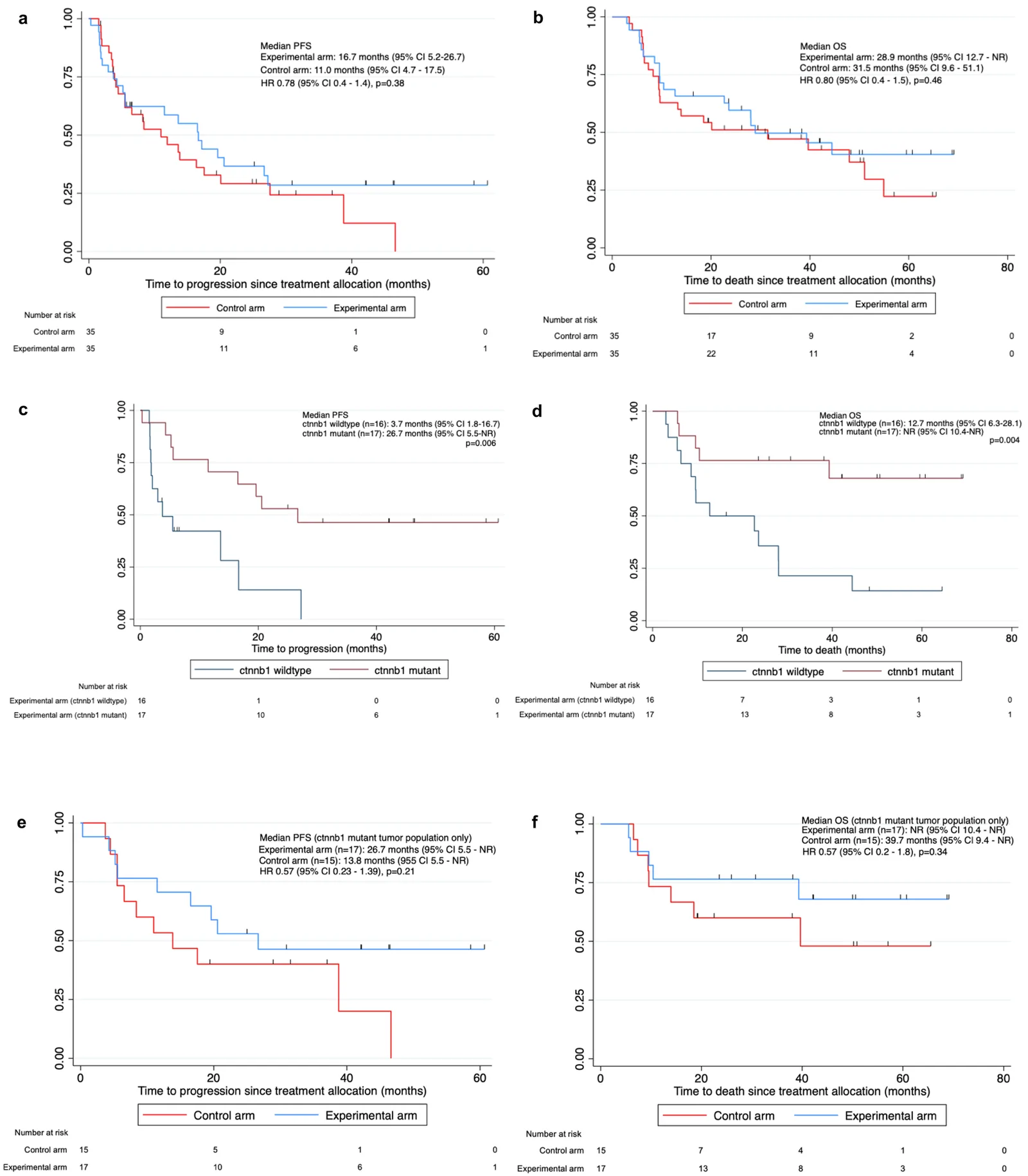
Kaplan-Meier curves demonstrating survival outcomes. **a** PFS in all-comers **b** OS in all-comers **c** PFS by *CTNNB1* status in the experimental arm **d** OS by *CTNNB1* status in the experimental arm **e** PFS in *CTNNB1*_mut_ tumors between treatment arms **f** OS in *CTNNB1*_mut_ tumors between treatment arms. CI confidence interval. Ctnnb1 mut *CTNNB1* mutant tumor. Ctnnb1 wt *CTNNB1* wild-type tumor. HR hazard ratio. NR not reached. OS overall survival. PFS progression-free survival. The Kaplan-Meier estimator was used to estimate PFS and OS Kaplan-Meier curves. The Cox proportional hazards model was used to estimate the HR, and the log-rank test was performed with two-sided p-values. Adjustments were not made for multiple comparisons.

**Fig. 4:**
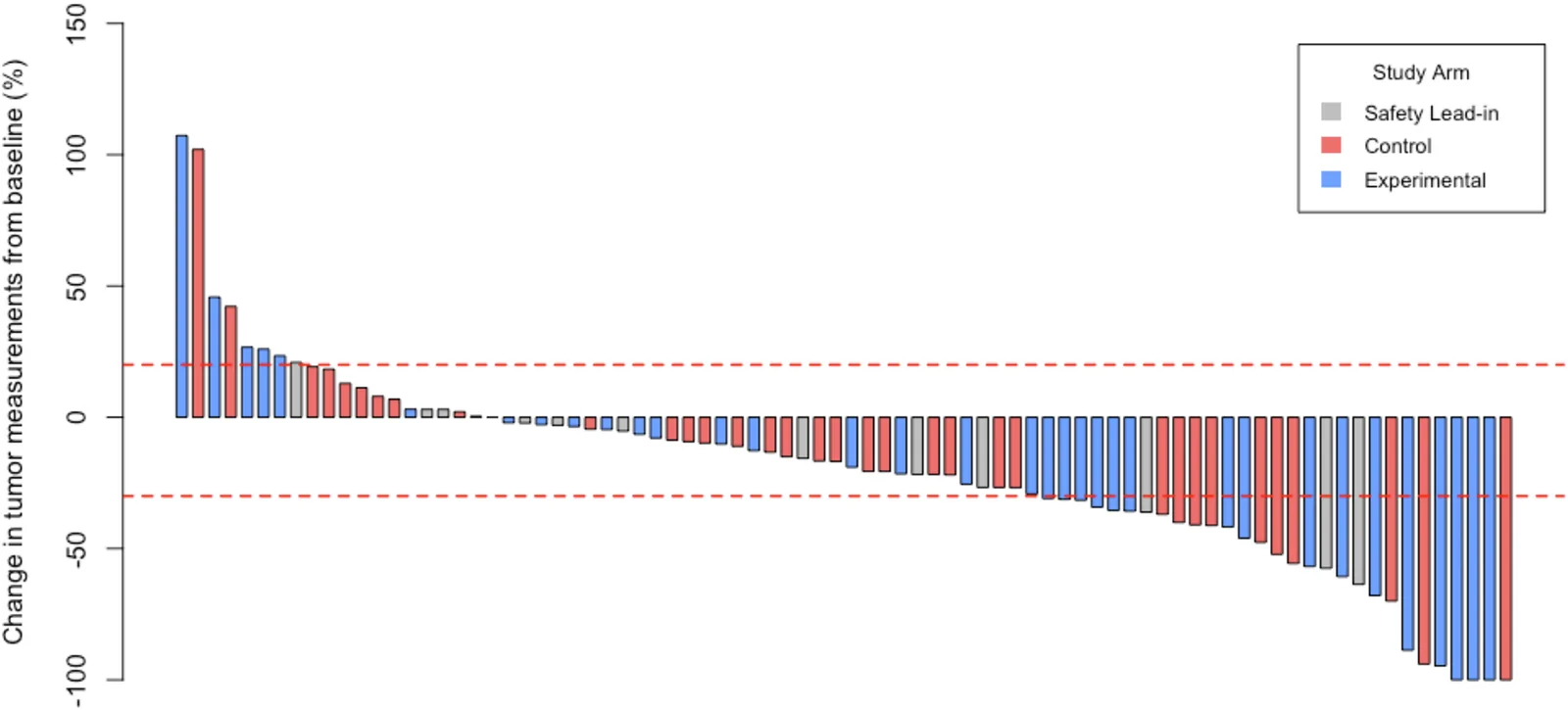
Best change in target lesions from baseline in all patients evaluable for RECIST 1.1 response. Horizontal lines at −30% and 20% denote partial response and disease progression by RECIST v1.1 criteria, respectively. All four patients with a complete response (three from the experimental arm and one from the control arm) did not have any evidence of disease recurrence at the time of data cutoff. Three patients in the safety lead-in and one patient in the experimental group were not evaluable for antitumor response and therefore are not included in the waterfall plot. Source data are provided as a Source Data file.

There was no significant difference in median PFS (16.7 vs 11.0 months; HR 0.78, 95% CI 0.4–1.4, *p* = 0.38) and OS (28.9 vs 31.5 months; HR 0.80, 95% CI 0.4–1.5, *p* = 0.46) between the experimental and control arms (Fig. 3a, b). The waterfall and spider plots demonstrate the best change in target lesions (Fig. 4) and tumor kinetics over time (Supplementary Fig. 1). There was no difference in ORR (44.1% vs 25.7%, *p* = 0.11) nor CBR (67.7% vs 68.6%, *p* = 0.93) (Supplementary Table 4). Despite no differences in ORR between arms, the experimental and control arms had three and one CR, respectively. In the experimental arm, two of these patients discontinued at cycles 16 and 34 due to treatment-related toxicity, and the remaining patient is still on treatment and has received 65 cycles. These patients have not had disease progression (PFS censored at 60.5, 46.1, and 58.4 months, respectively). In the control arm, the patient with a complete response is currently on treatment and has received 29 cycles (PFS censored at 27.7 months).

### Safety

Table 2 summarizes the treatment-related adverse events (TRAEs). Neutropenia (any [80% vs 25.7%, *p* < 0.001] and grade 3 [54.3% vs 5.7%, *p* < 0.001]) and leukopenia (48.6% vs 14.3%, *p* = 0.002) were significantly higher in the experimental arm. Hypertriglyceridemia (11.4% vs 37.1%, *p* = 0.02) and hypercholesterolemia (0% vs 28.6%, *p* = 0.001) were higher in the control arm. Dose interruption, dose level reduction, and treatment discontinuation of one or more drugs were similar between arms. Treatment discontinuation due to TRAE for all drugs occurred in one patient in the safety lead-in arm due to grade 4 elevated creatinine (after 59 cycles). In the control arm, four patients had treatment discontinuation of all drugs due to TRAE (grade 3 elevated ALT, grade 3 upper gastrointestinal hemorrhage, grade 2 lymphedema, and grade 2 pneumonitis). In the experimental arm, 3 patients discontinued all drugs due to TRAE (grade 3 hyperglycemia, grade 4 hypertriglyceridemia, and grade 2 fatigue). Ribociclib was discontinued in three and six patients in the safety lead-in and experimental arms, respectively (mainly due to neutropenia), but the patients continued treatment with everolimus plus letrozole.

**Table 2:** TRAE of any grade present in ≥20% of patients and grade ≥3 TRAE present in ≥5% of patients

	No. (%)			
		Randomized phase		
	Safety lead-in (*n* = 17)	Control Arm (*n* = 35)	Experimental Arm (*n* = 35)	*p*
Any grade TRAE				
Neutropenia	14 (82)	9 (26)	28 (80)	<0.001
Anemia	12 (71)	15 (43)	20 (57)	0.23
Leukopenia	12 (71)	5 (14)	17 (49)	0.002
Thrombocytopenia	9 (53)	6 (17)	11 (31)	0.16
Hyperglycemia	6 (35)	16 (46)	10 (29)	0.14
Creatinine increased	6 (35)	3 (9)	2 (6)	1.0
Hypertriglyceridemia	6 (35)	13 (37)	4 (11)	0.02
Hypercholesterolemia	3 (18)	10 (29)	0 (0)	0.001
Fatigue	3 (18)	7 (20)	9 (26)	0.57
Lung infection	1 (6)	3 (9)	8 (23)	0.19
Urinary tract infection	1 (6)	5 (14)	7 (20)	0.53
Grade ≥3 TRAE				
Neutropenia	11 (65)	2 (6)	19 (54)	<0.001
Thrombocytopenia	6 (35)	3 (9)	2 (6)	1.0
Leukopenia	6 (35)	1 (3)	10 (29)	0.07
Anemia	5 (29)	9 (26)	11 (31)	0.60
Hyperglycemia	1 (6)	3 (9)	6 (17)	0.48
Fatigue	2 (12)	0 (0)	1 (3)	1.0
Febrile neutropenia	2 (12)	0 (0)	1 (3)	1.0
Lung infection	1 (6)	2 (6)	4 (11)	0.67
Hypertriglyceridemia	1 (6)	4 (11)	2 (6)	0.67
Urinary tract infection	1 (6)	0 (0)	3 (9)	0.24
Abdominal pain	0 (0)	2 (6)	0 (0)	0.49
Acute kidney injury	1 (6)	1 (3)	0 (0)	1.0
ALT increased	0 (0)	2 (6)	1 (3)	0.49
AST increased	0 (0)	2 (6)	1 (3)	0.49
Creatinine increased	1 (6)	1 (3)	0 (0)	1.0
Diarrhea	1 (6)	1 (3)	1 (3)	1.0
Hypokalemia	0 (0)	0 (0)	2 (6)	0.49
Mucositis oral	0 (0)	1 (3)	2 (6)	1.0
Dose interruption, reduction, or discontinuation				
Dose interruption	14 (82)	27 (77)	33 (94)	0.08
Dose level reduction	5 (29)	11 (31)	17 (49)	0.14
Discontinuation ≥1 drug	4 (24)	4 (11)	9 (26)	0.22

N (%), *ALT* Alanine Aminotransferase, *AST* Aspartate Aminotransferase, *TRAE* treatment-related adverse event, Two-sided Chi-squared test was performed and two-sided Fisher exact tests when appropriate. Adjustments were not made for multiple comparisons.

### Impact of *CTNNB1* mutation status on efficacy—a prespecified analysis

**Fig. 5:**
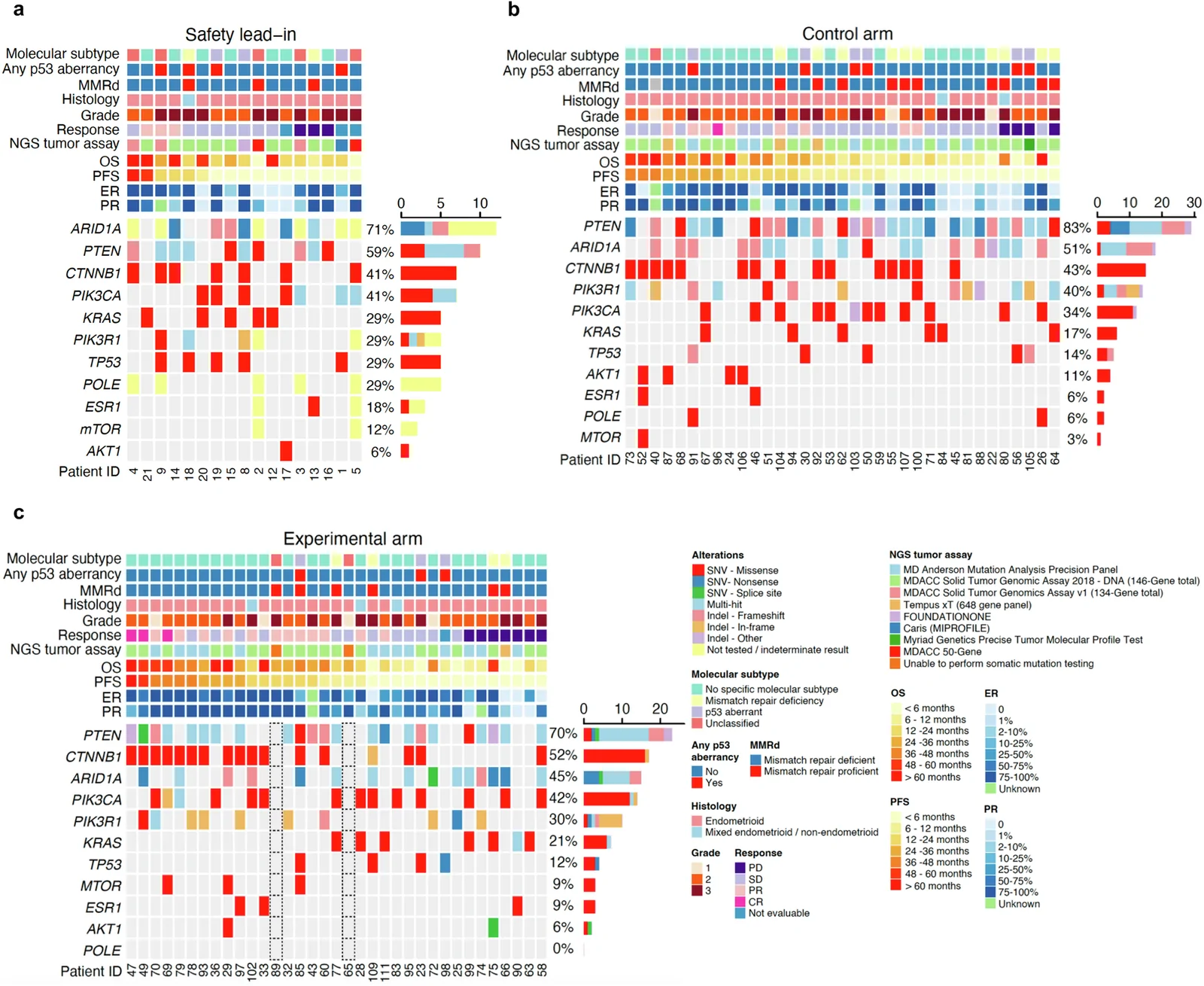
Molecular alterations and hormone receptor expression in evaluable patients by molecular subtype, histology, tumor grade, response, PFS, and OS. **a** Safety Lead-in **b** Control arm **c** Experimental arm. ER estrogen receptor. MDACC = MD Anderson Cancer Center. MMRd mismatch repair deficiency. NGS next-generation sequencing. OS overall survival. PFS progression-free survival. PR progesterone receptor. Response (PD progressive disease, SD stable disease, PR partial response, CR complete response). SNV single nucleotide variant. Please note that there were 6 patients in the control arm who did not have their ER expression levels quantified (patient IDs 22, 46, 55, 56, 84, 103) but had been interpreted as ER positive; in this situation, the ER expression level was quantified as 1%. Similarly, there were 4 patients in the control arm who did not have their PR expression level quantified (patient ID 22, 55, 56, 84) but were interpreted as PR positive; in this situation, the PR expression level was quantified as 1%. Source data are provided as a Source Data file.

32 patients (15 in the control arm and 17 in the experimental arm) had a *CTNNB1*_mut_ tumor, and all had pathogenic exon 3 mutations (Supplementary Table 5). Presence of *CTNNB1* mutations was associated with significantly improved clinical benefit in the experimental arm (Figs. 3c, d and  5). Within the experimental arm, those with *CTNNB1*_mut_ tumors had significantly higher CBR compared to those with *CTNNB1*_WT_ tumors (82.4% [14/17] vs 46.7% [7/15], *p* = 0.03). Additionally, those with *CTNNB1*_mut_ tumors had significantly longer median PFS compared to those with *CTNNB1*_WT_ tumors (26.7 vs 3.7 months; HR 0.27, 95% CI 0.1–0.7, *p* = 0.006) (Fig. 3c). Those with *CTNNB1*_mut_ tumors had significantly longer median OS compared to those with *CTNNB1*_WT_ tumors (not reached vs 12.7 months; HR 0.24, 95% CI 0.1–0.7, *p* = 0.004) (Fig. 3d). The 3-year Kaplan-Meier OS rate estimate was significantly higher for those with *CTNNB1*_mut_ tumors within the experimental arm (76.5% [standard error (SE) 10.3%] vs 21.4% [SE 10.8%], *p* = 0.0002; Z score 3.7) compared to those with *CTNNB1*_WT_ tumors. This was similarly observed for the 4-year Kaplan-Meier OS rate estimate (68.0% [SE 12.2%] vs 14.3% [SE 9.3%], *p* = 0.0004; Z score 3.5). There was no PFS and OS difference based on *CTNNB1*_mut_ status within the control arm (Supplementary Fig. 2a, b). There was no difference in PFS or OS between arms among *CTNNB1*_mut_ tumors (Fig. 3e, f). There was no difference in CBR between arms among *CTNNB1*_mut_ tumors (93.3% vs 82.4%, *p* = 0.60). Although there was no difference in ORR between arms among *CTNNB1*_mut_ tumors (58.8% vs 26.7%, *p* = 0.09), all three patients in the experimental arm with a CR had a *CTNNB1*_mut_ tumor with no evidence of disease at data cutoff (Fig. 2).

### Subgroup efficacy analyses

The impact of molecular profile on PFS and OS between (Table 3) and within (Table 4) arms is demonstrated. Between arms, there were no differences in PFS and OS based on molecular profile (Table 3) and patient characteristics (Supplementary Fig. 3). There was no difference in ORR (Table 5) or CBR (Supplementary Table 6) based on molecular profile. However, among those with prior endocrine therapy, ORR was significantly higher in the experimental arm compared to the control arm (61.5% vs 0.0%, *p* < 0.001). Within both arms, patients with grade 3 tumors had shorter PFS and OS compared to those with grade 1/2 tumors (Table 4). Among grade 1/2 tumors in the experimental arm (*n* = 22), PFS (HR 0.15, 95% CI 0.04–0.5; *p* = 0.002) and OS (HR 0.16, 95% CI 0.0–0.77; *p* = 0.01) were significantly longer among those with *CTNNB1*_mut_ tumors compared to those with *CTNNB1*_WT_ tumors (Table 4). PFS and OS benefit was not observed among grade 1/2 tumors based on *CTNNB1* mutation status in the control arm (Table 4).

**Table 3:** PFS and OS based on tumor grade and biomarker status between treatment arms

	PFS		OS	
	HR (95% CI)^a^	*p*	HR (95% CI)^a^	*p*
High tumor grade				
Low grade (*n* = 45)	0.73 (95% CI 0.3–1.6)	0.42	0.77 (95% CI 0.3–1.8)	0.53
High grade (*n* = 25)	0.86 (95% CI 0.4–2.1)	0.75	0.93 (95% CI 0.4–2.3)	0.87
*CTNNB1* mutation				
Wildtype (*n* = 36)	1.4 (95% CI 0.6–3.1)	0.40	1.2 (95% CI 0.6–2.6)	0.60
Mutant (*n* = 32)	0.57 (95% CI 0.2–1.4)	0.21	0.57 (95% CI 0.2–1.8)	0.34
*P53* aberrant				
Wildtype (*n* = 59)	0.73 (95% CI 0.4–1.4)	0.33	0.82 (95% CI 0.4–1.6)	0.57
Mutant (*n* = 10)	1.5 (95% CI 0.3–7.4)	0.64	1.5 (95% CI 0.3–6.2)	0.61
*PTEN* mutation				
Wildtype (*n* = 16)	1.5 (95% CI 0.4–5.1)	0.52	1.6 (95% CI 0.4–6.3)	0.51
Mutant (*n* = 52)	0.67 (95% CI 0.3–1.3)	0.23	0.67 (95% CI 0.3–1.4)	0.27
*PI3KCA* mutation				
Wildtype (*n* = 42)	0.81 (95% CI 0.4–1.7)	0.57	0.74 (95% CI 0.3–1.7)	0.47
Mutant (*n* = 26)	0.67 (95% CI 0.2–1.8)	0.44	0.91 (95% CI 0.3–2.4)	0.85
*AKT1* mutation				
Wildtype (*n* = 63)	0.66 (95% CI 0.4–1.2)	0.18	0.74 (95% CI 0.4–1.4)	0.34
Mutant (*n* = 5)	NE	NE	NE	NE
*mTOR* mutation				
Wildtype (*n* = 64)	0.85 (95% CI 0.5–1.5)	0.59	0.88 (95% CI 0.5–1.6)	0.69
Mutant (*n* = 4)	NE	NE	NE	NE
*PI3K* pathway alterations^b^				
Wildtype (*n* = 5)	NE	NE	NE	NE
Aberrant (*n* = 63)	0.70 (95% CI 0.4–1.3)	0.25	0.67 (95% CI 0.3–1.3)	0.23
*KRAS* mutation				
Wildtype (*n* = 55)	0.73 (95% CI 0.4–1.4)	0.34	0.79 (95% CI 0.4–1.6)	0.51
Mutant (*n* = 13)	1.4 (95% CI 0.3–6.5)	0.63	0.99 (95% CI 0.3–3.7)	0.99
MMR status				
Proficient (*n* = 53)	0.80 (95% CI 0.4–1.6)	0.53	0.76 (95% CI 0.4–1.5)	0.44
Deficient (*n* = 16)	0.41 (95% CI 0.1–1.5)	0.16	0.71 (95% CI 0.2–2.9)	0.62
ER/PR				
Negative (*n* = 4)	NE	NE	NE	NE
Any positivity (*n* = 65)	0.76 (95% CI 0.42–1.4)	0.37	0.72 (95% CI 0.4–1.4)	0.32
Low tumor grade				
*CTNNB1* wildtype (*n* = 21)	1.4 (95% CI 0.5–4.0)	0.50	1.2 (95% CI 0.4–3.2)	0.78
*CTNNB1* mutant (*n* = 23)	0.39 (95% CI 0.1–1.4)	0.13	0.38 (95% CI 0.1–2.1)	0.25
High tumor grade				
*CTNNB1* wildtype (*n* = 15)	0.97 (95% CI 0.3–3.4)	0.96	1.5 (95% CI 0.5–4.7)	0.48
*CTNNB1* mutant (*n* = 9)	0.59 (95% CI 0.1–2.7)	0.49	1.0 (95% CI 0.2–5.0)	0.99

*CI* confidence interval, *ER/PR* estrogen receptor/progesterone receptor, *HR* hazard ratio, *NE* not evaluable given too few cases, *MMR* mismatch repair, *OS* overall survival, *PFS* progression-free survival.

^a^Control arm is the reference.

^b^PI3K pathway alterations are defined by at least one mutation in the following: *PTEN*, *PIK3CA*, *AKT1*, or *mTOR*. Please note that numbers may not add up to 70 if there is data for the molecular characteristic is not present. Cox proportional hazards model and log-rank test were performed with two-sided p-values. Adjustments were not made for multiple comparisons. Source data are provided as a Source Data file.

**Table 4:** PFS and OS based on tumor grade and biomarker status within each arm

	PFS		OS	
	HR (95% CI)^a^	*p*	HR (95% CI)^a^	*p*
High tumor grade^b^				
Control (*n* = 35)	2.5 (95% CI 1.1–5.7)	0.04	2.6 (95% CI 1.1–6.6)	0.04
Experimental (*n* = 35)	2.4 (95% CI 1.0–5.8)	0.05	3.0 (95% CI 1.2–7.6)	0.02
*CTNNB1* mutation				
Control (*n* = 35)	0.47 (95% CI 0.2–1.1)	0.08	0.50 (95% CI 0.2–1.2)	0.12
Experimental (*n* = 33)	0.27 (95% CI 0.1–0.7)	0.006	0.24 (95% CI 0.1–0.7)	0.004
*P53* aberrant				
Control (*n* = 35)	1.2 (95% CI 0.4–3.6)	0.71	2.2 (95% CI 0.78–6.2)	0.16
Experimental (*n* = 34)	1.9 (95% CI 0.5–6.6)	0.37	2.9 (95% CI 0.83–10.5)	0.14
*PTEN* mutation				
Control (*n* = 35)	1.8 (95% CI 0.6–5.4)	0.24	1.9 (95% CI 0.6–6.7)	0.56
Experimental (*n* = 33)	0.75 (95% CI 0.3–1.9)	0.54	0.69 (95% CI 0.3–1.9)	0.47
*PI3KCA* mutation				
Control (*n* = 35)	1.2 (95% CI 0.5–2.9)	0.67	0.9 (95% CI 0.39–2.2)	0.81
Experimental (*n* = 33)	0.95 (95% CI 0.4–2.3)	0.90	1.3 (95% CI 0.5–3.2)	0.62
*AKT1* mutation				
Control (*n* = 35)	NE	NE	NE	NE
Experimental (*n* = 33)	NE	NE	NE	NE
*mTOR* mutation				
Control (*n* = 35)	NE	NE	NE	NE
Experimental (*n* = 33)	NE	NE	NE	NE
*PI3K* pathway alterations^c^				
Control (*n* = 35)	NE	NE	NE	NE
Experimental (*n* = 33)	NE	NE	NE	NE
*KRAS* mutation				
Control (*n* = 35)	1.7 (95% CI 0.6–4.6)	0.33	1.8 (95% CI 0.7–4.9)	0.28
Experimental (*n* = 33)	2.4 (95% CI 0.7–7.8)	0.18	1.9 (95% CI 0.7–5.3)	0.27
MMR status				
Control (*n* = 34)	3.5 (95% CI 1.4–8.6)	0.01	1.1 (95% CI 0.4–2.9)	0.82
Experimental (*n* = 35)	2.2 (95% CI 0.8–6.4)	0.15	1.1 (95% CI 0.31–3.6)	0.94
ER/PR				
Control (*n* = 34)	NE	NE	NE	NE
Experimental (*n* = 35)	NE	NE	NE	NE
*CTNNB1* mutation among low-grade tumors				
Control (*n* = 22)	0.40 (95% CI 0.1–1.2)	0.10	0.44 (95% CI 0.1–1.5)	0.17
Experimental (*n* = 22)	0.15 (95% CI 0.04–0.5)	0.002	0.16 (95% CI 0.03–0.77)	0.01
*CTNNB1* mutation among high-grade tumors				
Control (*n* = 13)	1.0 (95% CI 0.3–3.6)	0.99	0.96 (95% CI 0.2–3.9)	0.96
Experimental (*n* = 11)	0.79 (95% CI 0.2–3.6)	0.76	0.59 (95% CI 0.1–2.4)	0.45

*CI* confidence interval, *ER/PR* estrogen receptor/progesterone receptor, *HR* hazard ratio, *NE* not evaluable given too few wild-type or mutant/aberrant cases, *MMR* mismatch repair, *OS* overall survival, *PFS* progression-free survival.

^a^Wild-type mutation is the reference. For MMR status, mismatch repair proficient is the reference. Cox proportional hazards model and log-rank test were performed with two-sided p-values. Adjustments were not made for multiple comparisons.

^b^High tumor grade was defined as grade 3 and reference was grade 1 or 2. ^c^PI3K pathway alterations were defined by at least one mutation in the following: *PTEN*, *PIK3CA*, *AKT1*, or *mTOR*. Source data are provided as a Source Data file.

**Table 5:** ORR based on clinicopathologic characteristics and molecular status

	Control Arm	Experimental Arm	p^a^
Grade			
1 or 2	22.7% (5/22)	45.5% (10/22)	0.11
3	30.8% (4/13)	41.7% (5/12)	0.69
Histology			
Endometrioid	28.1% (9/32)	43.3% (13/30)	0.21
Mixed	0.0% (0/3)	50.0% (2/4)	0.43
Prior endocrine therapy			
No	47.4% (9/19)	33.3% (7/21)	0.37
Yes	0.0% (0/16)	61.5% (8/13)	<0.001
Prior aromatase inhibitor			
No	32.1% (9/28)	42.9% (12/28)	0.41
Yes	0.0% (0/7)	50.0% (3/6)	0.07
Prior radiation therapy			
No	17.6% (3/17)	38.9% (7/18)	0.26
Yes	33.3% (6/18)	50.0% (8/16)	0.32
Prior lines of systemic therapy			
0 or 1	29.4% (5/17)	47.1% (8/17)	0.29
≥2	22.2% (4/18)	41.2% (7/17)	0.29
*CTNNB1*			
Wildtype	25.0% (5/20)	26.7% (4/15)	0.91
Mutant	26.7% (4/15)	58.8% (10/17)	0.09
*P53* aberrant			
Wildtype	24.1% (7/29)	43.3% (13/30)	0.12
Aberrant	33.3% (2/6)	33.3% (1/3)	1.0
*PTEN*			
Wildtype	33.3% (2/6)	44.4% (4/9)	1.0
Mutant	24.1% (7/29)	43.5% (10/23)	0.14
*PI3KCA*			
Wildtype	26.1% (6/23)	50.0% (9/18)	0.12
Mutant	25.0% (3/12)	35.7% (5/14)	0.68
*AKT1*			
Wildtype	22.6% (7/31)	45.2% (14/31)	0.06
Mutant	50.0% (2/4)	0.0% (0/1)	1.0
*mTOR*			
Wildtype	26.5% (9/34)	37.9% (11/29)	0.33
Mutant	0.0% (0/1)	100.0% (3/3)	0.25
PI3K pathway mutation^b^			
Normal	0.0% (0/1)	0.0% (0/3)	NE
Aberrant	26.5% (9/34)	48.3% (14/29)	0.07
*KRAS*			
Wildtype	27.6% (8/29)	48.0% (12/25)	0.12
Mutant	16.7% (1/6)	28.6% (2/7)	1.0
MMR status			
Proficient	25.0% (6/24)	46.4% (13/28)	0.11
Deficient	30.0% (3/10)	33.3% (2/6)	1.0
ER/PR			
Negative	0.0% (0/3)	0.0% (0/1)	NE
Any positivity	29.0% (9/31)	45.5% (15/33)	0.18

*ER/PR* estrogen receptor/progesterone receptor, *NE* not evaluable given too few wildtype or mutant/aberrant cases, *MMR* mismatch repair.

^a^Chi-squared test or two-sided fisher exact test was performed as appropriate. Adjustments were not made for multiple comparisons.

^b^PI3K pathway alterations defined by at least one mutation in the following: *PTEN*, *PIK3CA*, *AKT*, or *mTOR*. Source data are provided as a Source Data file.

## Discussion

This study represents the first clinical trial evaluating the antitumor efficacy of the combination of a CDK4/6 inhibitor, mTOR inhibitor, and aromatase inhibitor in EC. The trial demonstrated the addition of ribociclib to everolimus/letrozole was feasible and tolerable but the experimental regimen did not significantly improve efficacy compared to the control regimen in all comers. The control arm represents a stringent comparison as everolimus plus letrozole is an active combination in EC (median PFS 3-6 months and median OS 14-31 months)^13–15^. The overperformance of the control arm may have limited the ability to detect a difference between arms at the specified sample size since sample size calculations were based on available PFS trial data reported by Slomovitz et al.^13^. In this study, the trial population was heterogeneous with the patients with serous histology having no treatment responses (0/11 patients) to everolimus and letrozole^13^. Given this finding, pure non-endometrioid tumors were excluded in the current trial design and this may have improved efficacy in the control arm. Nonetheless, potential antitumor benefit of the addition of ribociclib to everolimus/letrozole was observed in certain subgroups. Ribociclib may help overcome resistance in those who have received prior endocrine therapy (ORR 61.5% vs 0.0%; *p* < 0.001). With respect to our a priori hypothesis examining the benefit of adding ribociclib to everolimus/letrozole in *CTNNB1*_mut_ tumors, there were a number of patients with *CTNNB1*_mut_ tumors who had prolonged PFS and OS outcomes within the experimental arm.

The *CTNNB1* gene encodes β-catenin (key mediator of the WNT/β-catenin pathway). Normally localized at the cell membrane to support adhesion, mutant β-catenin translocates to the nucleus, interacts with transcription factors, and drives cell proliferation and endometrial carcinogenesis^9,19,20^. Representing up to half of patients in the no specific molecular profile (NSMP) category, *CTNNB1*_mut_ tumors are associated with worse prognosis^6,9,10,21^. At the time of trial design, there was not enough preliminary data to justify a *CTNNB1*_mut_ EC-only study. Nonetheless, our hypothesis was that ribociclib could enhance therapeutic efficacy of everolimus/letrozole for tumors with increased cyclin D1 (e.g. *CTNNB1*_mut_ tumors). The mandated protocol-prespecified enrollment of ≥14 patients with *CTNNB1*_mut_ tumors in the experimental arm was crucial as most trials evaluating CDK4/6 inhibitors have been single-arm studies with small numbers of patients with *CTNNB1*_mut_ tumors (e.g. <10), thus limiting mechanistic insight of these agents in this population^16,17,22^. With an EC study population enriched with pathogenic *CTNNB1*_mut_ tumors (32/70; 46%), there are multiple observations that provide support to the clinical benefit of adding ribociclib to everolimus plus letrozole in *CTNNB1*_mut_ tumors. Although not reaching statistical significance, potential difference in ORR favored the experimental arm compared to the control arm among *CTNNB1*_mut_ tumors. Three patients with *CTNNB1*_mut_ tumors in the experimental arm have durable, complete responses and without evidence of recurrence (PFS censored 46.1–60.5 months). Within the experimental arm, there was a near 7-fold improvement in median PFS with median OS not reached in *CTNNB1*_mut_ tumors compared to *CTNNB1*_WT_ tumors. Furthermore, the Kaplan-Meier 3-year and 4-year OS rate estimates were significantly higher among those with *CTNNB1*_mut_ tumors in the experimental arm. Notably within the safety lead-in arm, one patient with a *CTNNB1* mutation had stable disease without disease progression and had a PFS of 60.4 months prior to death unrelated to disease (urosepsis). While *CTNNB1*_mut_ are often associated with low-grade EC, PFS benefit was not solely attributable to tumor grade, as grade 1/2 *CTNNB1*_WT_ tumors showed expected PFS comparable to literature values^10,14,23^. Additionally, clinical benefit was observed in the experimental arm for *CTNNB1*_mut_ grade 1/2 tumors but not in the control arm.

It should be noted that despite this prespecified subgroup analysis of *CTNNB1*_mut_ tumors, the study was not powered to compare the efficacy of the experimental versus control arms in the *CTNNB1*_mut_ tumor population. Furthermore, the study was not powered to determine the impact of *CTNNB1* mutation status on treatment efficacy within the control arm. Although there was no statistically significant difference within the control arm based on *CTNNB1* status, there was trend towards better PFS and OS in among *CTNNB1*_mut_ tumors (Supplementary Fig. 3a, b). In the aforementioned phase II study by Slomovitz et al., all patients (4/4) with *CTNNB1*_mut_ endometrioid EC had response to everolimus/letrozole^13^. Thus, the study may not have been sufficiently powered to detect a beneficial signal that may be less pronounced within the control arm and between arms among *CTNNB1*_mut_ tumors. Future studies should be designed to evaluate these regimens in a larger *CTNNB1*_mut_ tumor population and further evaluate the interaction of *CTNNB1*_mut_ status (e.g., effect modification) on treatment response.

In a phase II randomized trial, Mirza et al. demonstrated that palbociclib plus letrozole had longer PFS than letrozole alone (8.3 vs 3.1 months, respectively); this difference is expected given low response rates of letrozole monotherapy (ORR 9.4%)^18,24^. In other single-arm trials of letrozole plus CDK4/6 inhibitor (e.g., palbociclib, ribociclib, or abemaciclib) in ER-positive EC ( ≥ 1–10% expression), median PFS has ranged from 5.4–9.1 months and median OS has ranged from 15.7–21.6 months^16–18^. Konstantinopoulos et al. reported that abemaciclib plus letrozole was associated with response in *CTNNB1*_mut_ EC tumors (ORR 100%; 3/3) on exploratory analyses^16^. Additionally, in another single-arm study by Konstantinopoulos et al., *CTNNB1*_mut_ EC tumors were associated with clinical benefit with the use of abemaciclib, letrozole, and metformin, with two of three complete responses from tumors harboring a *CTNNB1*_mut_ tumor^25^. In a single-arm phase II trial by Green et al., patients with ER/PR-positive EC were treated with abemaciclib plus fulvestrant (≤1 prior line of endocrine therapy) had a median PFS and OS of 9.0 and 37.8 months, respectively^22^.

In contrast with other EC studies evaluating CDK4/6 inhibitors plus endocrine therapy, this trial included endometrioid/mixed-endometrioid tumors irrespective of ER/PR status positivity. Both treatment arms demonstrated clinical activity in a population that may not be considered low-risk (e.g., 40% had grade 3 tumors) or treatment-naïve (e.g., median number of prior lines of therapy was 2, and 40% had prior endocrine therapy). Activation of the PI3K/AKT/mTOR pathway is a key mechanism of intrinsic and acquired resistance to CDK4/6 inhibitors^26–28^. Preclinical studies have demonstrated that the combination of mTOR and CDK4/6 inhibition resulted in a profound reduction in cyclin D1, RB phosphorylation, E2F-mediated transcription, and tumor size compared with CDK4/6 inhibition alone or with endocrine therapy^26,29^. Thus, a combination of an mTOR inhibitor and CDK4/6 inhibitor may be synergistic, particularly in tumors with increased cyclin D1 expression (e.g., *CTNNB1*_mut_ tumors). Targeting the WNT/β-catenin pathway has been challenging, as early drug development of direct WNT pathway-inactivating agents has been limited by treatment-related toxicity^30^. Thus, the addition of ribociclib to everolimus and letrozole represents a potentially actionable method to target the WNT/β-catenin pathway. These study findings are particularly important given that ascertaining effective treatments for MMR-proficient EC in the NSMP category is becoming an increasing area of need. *CTNNB1*_mut_ tumors represent a significant portion of the NSMP category, and the experimental regimen in this population should be investigated in larger confirmatory studies.

Strengths of this study include its randomized, biomarker-driven design, comprehensive molecular profiling, and inclusion of a diverse population of women (reflecting a more representative recurrent EC population) and nearly half the patients with high-grade tumors. This trial included enrollment at a nearby partner safety-net hospital to reduce barriers to clinical trial participation for medically underserved groups. Approximately half of the study population had moderately high to high SVI and ADI scores. Patients living in areas with high SVI and ADI scores generally have limited access and significant barriers to clinical trial participation^31,32^. The use of a stringent comparator arm is another strength that enables better evaluation of the added benefit of CDK4/6 inhibitors, given that all trials that have evaluated CDK4/6 inhibitors plus endocrine therapy (other than Mirza et al.) have been single-arm studies^16–18,22^.

Limitations include a relatively small study population, which restricts the generalizability of subgroup analyses, particularly for other somatic mutations and molecular characteristics. The subgroup analysis results are hypothesis-generating and future larger studies are needed to validate these findings. Despite recruitment of an overall diverse study population (41% of patients in the study population that were non-White and/or Hispanic compared to 10-20% in the majority of other trials), the enrollment of Black patients was low and greater strides are needed increase enrollment of this group that are most negatively impacted by EC disparities^3^. Improving trial access to underrepresented and vulnerable groups is critical to eliminating the dramatic racial disparities in EC mortality rates and provides a more real-world representation of clinical practice and influences of social determinants of health on tumor biology. Another limitation may be the lack of requirement for ER/PR positivity in trial eligibility criteria and this may have led to a possible decrease in treatment efficacy. The trial inclusion criteria required endometrioid histology which is known to have high prevalence of ER/PR positivity, even in poorly differentiated endometrioid tumors. Thus, the majority (>90%) had ER/PR positivity in the randomized phase portion and the impact of mandating ER/PR stain positivity is likely minimal. Although ER/PR staining is becoming routinely performed in clinical practice for endometrial cancer, it was not routinely performed at trial initiation and may have served as another barrier/delay to trial enrollment, particularly in underserved populations.

In conclusion, we observed significant, durable antitumor activity of everolimus and letrozole with or without ribociclib in advanced/recurrent endometrioid EC. Despite no observed statistical difference in efficacy between overall treatment arms, the addition of ribociclib to everolimus and letrozole demonstrated prolonged response in tumors harboring *CTNNB1* mutations and should be examined in larger, future trials with this molecular profile.

## Methods

### Study design and population

This was an open-label, randomized phase II trial conducted at The University of Texas MD Anderson Cancer Center and Lyndon B. Johnson General Hospital (ClinicalTrials.gov identifier: NCT03008408; date of registration: 8-18-2017. This investigator-initiated study was approved by the MD Anderson and Lyndon B. Johnson General Hospital institutional review boards and adhered to the principles of the Declaration of Helsinki and the International Conference on Harmonization Good Clinical Practice guidelines. All patients provided written informed consent prior to enrollment in the study. The study funding and drugs (everolimus, letrozole, and ribociclib) were provided by Novartis (Basel, Switzerland).

Key eligibility criteria included ≥18 years old, histologically-confirmed endometrioid or mixed endometrioid EC, advanced/recurrent disease not amenable to curative therapy, Eastern Cooperative Oncology Group Performance Status (ECOG PS) 0-1, measurable disease per RECIST v1.1 criteria, and no more than two prior chemotherapeutic regimens in the recurrent setting. Prior exposure to non-chemotherapy systemic regimens (e.g., endocrine therapies, immunotherapy, targeted therapy, antibody-drug conjugates) was permissible and not counted in the maximum number of prior lines. Prior treatment with letrozole was permitted, but those with prior exposure to mTOR inhibitors were excluded. Estrogen/progesterone receptor (ER/PR) positivity was not required. Patients with uterine sarcomas, carcinosarcomas, and pure serous or clear cell carcinomas were excluded. Prespecified-protocol enrollment of ≥14 patients with a *CTNNB1* mutation in the experimental arm was required.

Sex, ethnicity, and race were not prospectively collected from patients as part of their enrollment in the clinical trial. This information was retrospectively collected from self-reported data at the time of new patient registration in each institution.

### Study procedures

The study was divided into two phases: safety lead-in and dose expansion phase. A safety lead-in was performed to identify the recommended phase 2 dose (RP2D) combination of everolimus, letrozole, and ribociclib prior to initiation of randomization. The starting ribociclib and everolimus dosages were chosen based on safety data of dose optimization studies in breast cancer utilizing ribociclib, everolimus, and exemestane^33,34^. Given that ribociclib is a CYP3A4 inhibitor and everolimus is a CYP3A4 substrate, ribociclib could increase everolimus exposure 3.5 fold and thus the starting oral dosages were ribociclib 250 mg daily, everolimus 2.5 mg daily, and letrozole 2.5 mg daily^35,36^. Pharmacokinetic studies have demonstrated that everolimus at 2.5 mg daily and ribociclib 200–250 mg daily have both drugs in the therapeutic window^33,34^. Enrollment into the safety lead-in occurred at six patient increments (up to 24 patients), starting at the starting dose level (DL) 0 (Supplementary Table 1). If ≤1 of 6 patients at DL 0 had a dose-limiting toxicity (DLT), then the regimen was deemed safe for administration in the dose expansion phase. If two or more patients in DL 0 experienced a DLT, then the regimen would be dose-reduced, and 6 additional patients would be enrolled at DL −1. If two or more patients in DL −1 experienced a DLT, then the regimen would be dose-reduced, and 6 additional patients would be enrolled at DL −2, and so forth. If ≤1 of 6 patients experienced a DLT at DL −1, then an additional 6 patients would be enrolled to evaluate the dose increase of everolimus at 5 mg daily (DL −1A), given interim breast cancer trial data had demonstrated safety at various dosages of ribociclib 200–300 mg with everolimus at 5 mg daily^33,34^. If ≤1 of 6 patients experienced a DLT at DL −1A, then DL −1A would be chosen as the RP2D; otherwise, DL −1 would be chosen as the RP2D. Details of the safety lead-in are demonstrated in Fig. 1a and the RP2D for the experimental arm was daily oral everolimus 2.5 mg, letrozole 2.5 mg, and ribociclib 200 mg in 28-day cycles (DL −1).

Once the RP2D was determined, eligible patients were randomly assigned 1:1 in the dose expansion phase, via the OnCore clinical trial management system (Advarra,Inc, Columbia, Maryland) to either the experimental (daily oral everolimus 2.5 mg, letrozole 2.5 mg, and ribociclib 200 mg in 28-day cycles) or the control arm (daily oral everolimus 10 mg and letrozole 2.5 mg in 28-day cycles). All patients commenced treatment within 7 days following randomization, and treatment continued until radiologic disease progression (RECIST v1.1) or unacceptable toxicity.

Tumor assessments were performed at baseline, week 8, week 16, week 24, then every 12 weeks until disease progression. Adverse events (AE) were recorded at every cycle until 30 days after the last dose of treatment and were graded according to National Cancer Institute Common Terminology Criteria for Adverse Events (CTCAE) version 4.0.

### Biomarker evaluation

All patients underwent a pre-treatment tumor biopsy but were exempt if there was archival formalin-fixed paraffin-embedded tumor tissue from a biopsy within 3 months of the planned treatment start. When feasible, somatic tumor mutation testing using next-generation panel sequencing (NGS) was performed on pre-treatment tumor biopsies (archival formalin-fixed, paraffin-embedded tissue was used if a biopsy was performed within 3 months of planned treatment start). The default preference was made to utilize the MD Anderson institutional NGS panel testing (Supplementary Table 2). Given the evolving institutional NGS platform/technology, the most up-to-date panel version was utilized as patients were enrolled in the study. If the MD Anderson NGS panel could not be utilized, alternative commercially available NGS panels were utilized. Additionally, immunohistochemistry staining on pre-treatment tumor biopsies was performed for p53, mismatch repair proteins (MMR), and ER/PR; these were all centrally reviewed by two gynecologic oncology study pathologists. A tumor was considered ER/PR positive if there was at least 1% expression.

### Study endpoints

The primary endpoint was PFS (time from treatment allocation to disease progression [RECIST v1.1] or death). Patients were censored at the last visit evaluating for disease status if they did not have disease progression at the time of data cutoff. If they had discontinued treatment prior to disease progression due to other reasons (e.g., toxicity or withdrawal of consent), PFS was evaluated until disease progression and censored at the start date of a new anticancer therapy if it was initiated prior to disease progression. Secondary endpoints included overall survival (OS), objective response rate (ORR), clinical benefit rate (CBR), toxicity, and the presence of a somatic *CTNNB1* mutation and its association with treatment response. OS was defined as time from treatment allocation to death (censored at the last known date alive). CBR was defined as having prolonged stable disease (SD; ≥16 weeks) or partial (PR) or complete (CR) response by RECIST v1.1. Exploratory endpoints included correlation between efficacy endpoints and other somatic mutations. Post-hoc analyses included correlation between efficacy endpoints and other molecular features (e.g., p53, MMR, and ER/PR) or clinicopathologic characteristics. All analyses were preplanned, unless specified. Efficacy and safety analyses were performed on all patients who received at least one dose of study drug.

### Statistics and reproducibility

Descriptive statistics were used to summarize study population characteristics. Social Vulnerability Index (SVI) and Area Deprivation Index (ADI) scores were calculated to describe neighborhood-level social determinants of health using United States Census Bureau-level patient data based on previously described methods^37–41^.

PFS and OS were estimated with the Kaplan-Meier product-limit estimator and compared between treatment arms with the log-rank test. Cox proportional hazards model was used to assess the treatment arm difference (HR and 95% CI). ORR and CBR were estimated using exact 95% binomial confidence intervals with the Clopper-Pearson method. The relationship between treatment response and tumor molecular features was assessed using two-tailed Fisher exact tests or the Cox proportional hazards model as appropriate. Available case analysis was used to handle missing data.

A sample size of 35 evaluable patients per arm would provide 80% power to detect an improvement in median PFS from three to six months (HR of 0.5, with alpha level of 0.05). To evaluate the effect of *CTNNB1* mutations on treatment response, we prespecified enrollment ≥14 patients with a *CTNNB1* mutation in the experimental arm. All patients underwent somatic tumor NGS testing and tracking for enrollment of patients with *CTNNB1* mutations was reviewed monthly at the scheduled trial team meetings. We used Fisher’s exact test (or log-rank test if appropriate) to compare patients with or without *CTNNB1* mutation with respect to CBR, ORR, PFS, and OS. With 14 of patients with *CTNNB1* mutation in the experimental arm, there was 80% power with a one-sided significance level of 0.10 to detect a difference in CBR rates of 0.33 for patients without *CTNNB1* mutation and 0.70 for patients with *CTNNB1* mutation.

Futility monitoring for PFS and toxicity monitoring in the dose expansion phase was performed using a Bayesian optimal phase 2 (BOP2) design (see protocol for details)^42^. The investigators were not blinded to allocation during treatment and outcome assessment.

### Reporting summary

Further information on research design is available in the Nature Portfolio Reporting Summary linked to this article.

## Supplementary information

Supplementary Information

Reporting Summary

Transparent Peer Review file

## Source data

Source data

Source Data

## Data Availability

The study protocol (with the statistical analysis plan) is available in the Supplementary Information file. Source data are provided with this paper. Requests for additional de-identified clinical data not included in the source data file should be directed to the corresponding author and will be evaluated on a case-by-case basis. The reason for access restrictions is our Institutional Review Board (IRB) policy. Requests should be made to the corresponding author beginning 3 months and ending 5 years after the publication of the article. The expected timeframe for response to access requests is within 30 business days. Once access has been granted, the data will be available without restriction for general research use and indefinitely. Source data are provided with this paper.
