# Supplementary material for: Efficacy of everolimus and letrozole with or without ribociclib in recurrent endometrial cancer: a phase 2 randomized clinical trial
Source: Nat Commun. 2026 Sep 1;17:10413. doi: 10.1038/s41467-026-77049-3 (PMC13631232; doi:10.1038/s41467-026-77049-3)
Supplement: Supplementary file 1 [file 41467_2026_77049_MOESM1_ESM.pdf]

Supplement data

Supplementary Table 1 – Dose levels for everolimus, letrozole, and ribociclib in safety lead-in phase
Supplementary Table 2 – Details of next-generation sequencing panel testing
Supplementary Table 3 – Molecular and biomarker profile of the study population
Supplementary Table 4 – Treatment response per RECIST v1.1 criteria
Supplementary Table 5 – *CTNNB1* mutation (DNA and protein alterations)
Supplementary Table 6 – CBR based on clinicopathologic characteristics and molecular status
Supplementary Figure 1 – Spiderplot for tumor change by RECIST v1.1
Supplementary Figure 2 – PFS and OS by *CTNNB1* status in the control arm
Supplementary Figure 3 – Subgroup analyses for PFS and OS

Supplementary Table 1 – Dose levels for everolimus, letrozole, and ribociclib in the safety lead-in phase

DL	Everolimus dose	Letrozole dose	Ribociclib dose
0	2.5 mg daily	2.5 mg daily	250 mg daily
-1A	5 mg daily	2.5 mg daily	200 mg daily
-1	2.5 mg daily	2.5 mg daily	200 mg daily
-2	2.5 mg daily	2.5 mg daily	250 mg daily<br>(21 days on and 7 days off)
-3	2.5 mg daily	5 mg daily	200 mg daily<br>(21 days on and 7 days off)

DL = Dose level.

Supplementary Table 2 – Details of next-generation sequencing panel testing

NGS panel	N
MD Anderson institutional NGS panels	
Solid Tumor Genomic Assay v2[a]	52
Mutation Analysis Precision Panel[b]	21
Solid Tumor Genomics Assay v1[c]	3
50-Gene Somatic Mutation Analysis Panel[d]	2
Commercially available NGS panels	
Tempus xT (648 gene panel)	4
FOUNDATIONONE	1
Caris (MIPROFILE)	1
Myriad Genetics Precise Tumor Molecular Profile Test	1
Unable to perform somatic mutation testing	2

NGS = next-generation sequencing.

[a]Solid Tumor Genomic Assay v2: A next generation sequencing (NGS)-based analysis for the detection of somatic mutations in the coding sequence of 134 genes and selected copy number variations (amplifications) in 47 genes (overlap: 146 genes total) was performed on the DNA extracted from the sample in a CLIA-certified molecular diagnostics laboratory.

[b]The MD Anderson Mutation Analysis Precision Panel™ (MDA MAPP) assay is a custom high-throughput next generation sequencing-based CLIA assay that uses targeted hybridization-based capture technology for detection of sequence variants/mutations in 610 genes (single nucleotide variants [SNVs] and insertion/deletion alterations [indels]), copy number variants (CNVs) in 583 genes, select gene rearrangements in 34 genes (Fusions), and selected genomic immuno-oncology signatures including microsatellite instability (MSI) and tumor mutational burden (TMB) in DNA isolated from formalin-fixed paraffin embedded (FFPE) tumor tissue and cytology specimens.

[c]Solid Tumor Genomics Assay v1: A next generation sequencing (NGS)-based analysis for the detection of somatic mutations in the coding sequence of 128 genes and selected copy number variations (amplifications) in 49 genes (overlap: 134 genes total) was performed on the DNA extracted from the sample in a CLIA-certified molecular diagnostics laboratory.

[d]50-Gene Somatic Mutation Analysis Panel: A next generation sequencing (NGS)-based analysis for the detection of somatic mutations in the coding sequence of a total of 50 genes was performed on the DNA extracted from the sample in a CLIA-certified molecular diagnostics laboratory. This panel was performed when there was a lack of a NGS blood control.

Supplementary Table 3 – Molecular and biomarker profile of the study population

	No. (%)		
	Safety lead-in (n=17)	Control Arm (n=35)	Experimental Arm (n=35)
P53 aberrant[a]			
Wildtype	12 (71)	29 (83)	30 (88)
Aberrant	5 (29)	6 (17)	4 (12)
Unknown	0	0	1
CTNNB1 mutation			
Wildtype	10 (59)	20 (57)	16 (48)
Mutant	7 (41)	15 (43)	17 (52)
Unknown	0	0	2
PTEN mutation			
Wildtype	7 (41)	6 (17)	10 (30)
Mutant	10 (59)	29 (83)	23 (70)
Unknown	0	0	2
PIK3CA mutation			
Wildtype	10 (59)	23 (66)	19 (58)
Mutant	7 (41)	12 (34)	14 (42)
Unknown	0	0	2
mTOR mutation			
Wildtype	15 (100)	34 (97)	30 (91)
Mutant	0 (0)	1 (3)	3 (9)
Unknown	2	0	2
AKT1 mutation			
Wildtype	16 (94)	31 (89)	32 (97)
Mutant	1 (6)	4 (11)	1 (3)
Unknown	0	0	2
PI3K pathway aberrations[b]			
Wildtype	2 (12)	1 (3)	4 (12)
Aberrant	15 (88)	34 (97)	29 (88)
Unknown	0	0	2
KRAS mutation			
Wildtype	12 (71)	29 (83)	26 (79)
Mutant	5 (29)	6 (17)	7 (21)
Unknown	0	0	2
MMR status			
Proficient	14 (82)	24 (71)	29 (83)
Deficient	3 (18)	10 (29)	6 (17)
Unknown	0	1	0
ER/PR			
Negative	2 (12)	3 (9)	1 (3)
Any positivity	15 (88)	31 (91)	34 (97)
Unknown	0	1	0
Molecular category			
POLE	0 (0)	0 (0)	0 (0)
MMRd	2 (17)	10 (29)	4 (12)
P53 aberrant	3 (25)	6 (18)	3 (9)
NSMP	7 (58)	18 (53)	26 (79)
Unclassified[c]	5	1	2

ER/PR = estrogen receptor/progesterone receptor. NSMP = no specific molecular profile. MMR = mismatch repair. [a]p53 aberrancy by immunohistochemistry or *TP53* mutant. [b]PI3K pathway alterations defined by at least one mutation in the following: *PTEN*, *PIK3CA*, *AKT1*, or *mTOR*. [c]Unclassified due to 1

or more missing tests (*POLE* mutation testing, p53 IHC/somatic testing, or MMR IHC testing). Source data are provided as a Source Data file.

Supplementary Table 4 – Treatment response per RECIST v1.1 criteria

	Safety lead-in (n=17)	Control Arm (n=35)	Experimental Arm (n=35)	p[a]
BOR				0.14
CR	0% (0/14)	2.9% (1/35)	8.8% (3/34)	
PR	21.4% (3/14)	22.9% (8/35)	35.3% (12/34)	
SD	57.4%(8/14)	62.9% (22/35)	35.3% (12/34)	
PD	21.4% (3/14)	11.4% (4/35)	20.6% (7/34)	
NE	3	0	1	
ORR	21.4% (3/14)	25.7% (9/35)	44.1% (15/34)	0.11
CBR	64.3% (9/14)	65.7% (23/35)	67.7% (23/34)	0.93
DOR[b]	16.3 months (13.2 – 30.9)	16.1 months (2.6 – 47.3)	20.1 months (2.2 – 58.7)	0.31

BOR = best objective response. CBR = clinical benefit rate (PR + CR + SD ≥16 weeks). CR = complete response. DOR = duration of response. NE = not evaluable for response. ORR = objective response rate (PR + CR). PR = partial response, PD = disease progression, SD = stable disease.[a]p-values were calculated using Chi-squared or Fisher's exact test when appropriate; two-sided p-values were obtained. Comparisons were made between the experimental and control arms. [b]Duration of response among patients with response by RECIST v1.1 criteria (e.g. PR or CR) summarized in median (range). Source data are provided as a Source Data file.

Supplementary Table 5 – *CTNNB1* mutation (DNA and protein alterations)

Subject ID	DNA	Protein	Location	Type
4	c.101G>T	p.G34V	Exon 3	SNV- Missense
5	c.121A>G	p.T41A	Exon 3	SNV- Missense
8	c.100G>A	p.G34R	Exon 3	SNV- Missense
9	c.97T>C	p.S33P	Exon 3	SNV - Missense
14	c110>T	p.S37F	Exon 3	SNV - Missense
17	c.98C>T	p.S33F	Exon 3	SNV - Missense
19	c.98C>G	p.S33C	Exon 3	SNV - Missense
23	c.100G>C	p.G34R	Exon 3	SNV - Missense
29	c.94G>C	p.D32H	Exon 3	SNV - Missense
33	c.122C>T	p.T41I	Exon 3	SNV - Missense
40	c.110C>T	p.S37F	Exon 3	SNV - Missense
45	c.110C>T	p.S37F	Exon 3	SNV - Missense
46	c.94G>T	p.D32Y	Exon 3	SNV - Missense
47	c.134C>T	p.S45F	Exon 3	SNV - Missense
49	c.94G>T	p.D32Y	Exon 3	SNV - Missense
52	c.110C>T	p.S37F	Exon 3	SNV - Missense
53	c.94G>C	p.D32H	Exon 3	SNV - Missense
55	c.98C>A	p.S33Y	Exon 3	SNV - Missense
58	c.101G>A	p.G34E	Exon 3	SNV - Missense
59	c.98C>A	p.S33Y	Exon 3	SNV - Missense
60	c.94G>T	p.D32Y	Exon 3	SNV - Missense
68	c.98C>G	p.S33C	Exon 3	SNV - Missense
69	c.122C>T	p.T41I	Exon 3	SNV - Missense
70	c.94G>T	p.D32Y	Exon 3	SNV - Missense
73	c.122C>T	p.T41I	Exon 3	SNV - Missense
78	c.100G>A	p.G34R	Exon 3	SNV - Missense
79	c.101G>T	p.G34V	Exon 3	SNV - Missense
85	c.121A>G	p.T41A	Exon 3	SNV - Missense
87	c.101G>T	pG34V	Exon 3	SNV - Missense
92	c.100G>A	p.G34R	Exon 3	SNV - Missense
93	c.122C>T	p.T41I	Exon 3	SNV - Missense
95	c.110C>G	p.S37C	Exon 3	SNV - Missense
97	c.110C>G	p.S37C	Exon 3	SNV - Missense
100	c.101G>A	p.G34E	Exon 3	SNV - Missense
102	c.109T>G	p.S37A	Exon 3	SNV - Missense
104	c.98C>G	p.533C	Exon 3	SNV - Missense
106	c.104T>G	p.I35S	Exon 3	SNV - Missense
107	c.121A>G	pT41A	Exon 3	SNV - Missense
109	c.133_135del	p.S45del	Exon 3	deletion

SNV=single nucleotide variant. Source data are provided as a Source Data file.

Supplementary Table 6 – CBR based on clinicopathologic characteristics and molecular status

	Control Arm	Experimental Arm	p[a]
Grade			
1 or 2	81.8% (18/22)	72.7% (16/22)	0.72
3	46.2% (6/13)	58.3% (7/12)	0.54
Histology			
Endometrioid	75.0% (24/32)	33.3% (20/30)	0.47
Mixed	0.0% (0/3)	75.0% (3/4)	0.14
Prior endocrine therapy			
No	68.4% (13/19)	61.9% (13/21)	0.67
Yes	68.8% (11/16)	76.9% (10/13)	0.70
Prior aromatase inhibitor			
No	64.3% (18/28)	64.3% (18/28)	1.0
Yes	85.7% (6/7)	83.3% (5/6)	1.0
Prior radiation therapy			
No	70.6% (12/17)	61.1% (11/18)	0.56
Yes	66.7% (12/18)	75.0% (12/16)	0.72
Prior lines of systemic therapy			
0 or 1	64.7% (11/17)	64.7% (11/17)	1.0
≥2	72.2% (13/18)	70.6% (12/17)	0.92
CTNNB1			
Wildtype	50.0% (10/20)	46.7% (7/15)	0.85
Mutant	93.3% (14/15)	82.4% (14/17)	0.60
P53 aberrant			
Wildtype	72.4% (21/29)	70.0% (21/30)	0.84
Aberrant	50.0% (3/6)	33.3% (1/3)	1.0
PTEN			
Wildtype	83.3% (5/6)	66.7% (6/9)	0.60
Mutant	65.5% (19/29)	65.2% (15/23)	0.98
PI3KCA			
Wildtype	65.2% (15/23)	66.7% (12/18)	0.92
Mutant	75.0% (9/12)	64.3% (9/14)	0.68
AKT1			
Wildtype	67.7% (21/31)	67.7% (21/31)	0.79
Mutant	100.0% (4/4)	0.0% (0/1)	0.20
mTOR			
Wildtype	67.6% (23/34)	62.1% (18/29)	0.64
Mutant	100.0% (1/1)	100.0% (3/3)	NE
PI3K pathway mutation[b]			
Wildtype	0.0% (0/1)	0.0% (0/3)	NE
Aberrant	70.6% (24/34)	72.4% (21/29)	0.87
KRAS			
Wildtype	69.0% (20/29)	72.0% (18/25)	0.81
Mutant	66.7% (4/6)	42.9% (3/7)	0.59
MMR status			
Proficient	70.8% (17/24)	71.4% (20/28)	0.72
Deficient	60.0% (6/10)	50.0% (3/6)	1.0
ER/PR			
Negative	0.0% (0/3)	0.0% (0/1)	NE
Any positivity	74.2% (23/31)	69.7% (23/33)	0.78

ER/PR = estrogen receptor/progesterone receptor. NE = not evaluable given too few wildtype or mutant/aberrant cases. MMR = mismatch repair. [a]Chi-squared test or two-sided fisher test was performed as appropriate. Adjustments for multiple comparisons were not performed. [b]PI3K pathway alterations

defined by at least one mutation in the following: *PTEN*, *PIK3CA*, *AKT*, or *mTOR.* Source data are provided as a Source Data file.

Supplementary Figure 1 – Spiderplot for tumor change by RECIST v1.1

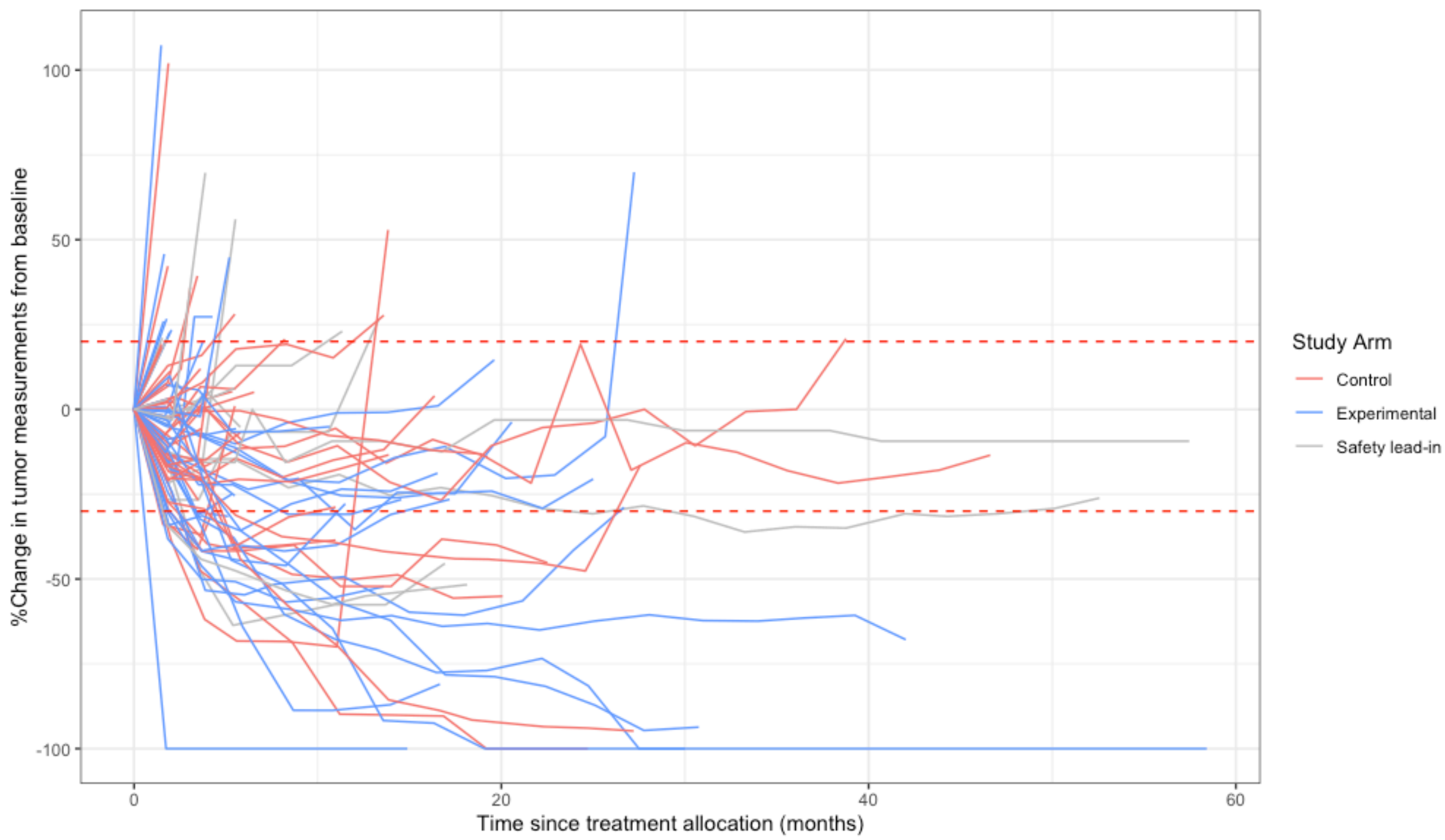

Legend: Please note that subject 78 on the experimental arm is not shown after 10.6 months given that disease was not evaluable by RECIST criteria after cycle 13 (target lesions obscured by bronchiectasis and atelectasis/fibrosis). Subject 78 had otherwise stable disease (-5.11% from baseline) and continued treatment until withdrawal of consent at cycle 43 (38 months). At the time of data cutoff, she has not had disease progression (PFS censored at 41.92 months) since stopping treatment. Source data are provided as a Source Data file.

Supplementary Figure 2 – PFS and OS by *CTNNB1* status in the control arm

a

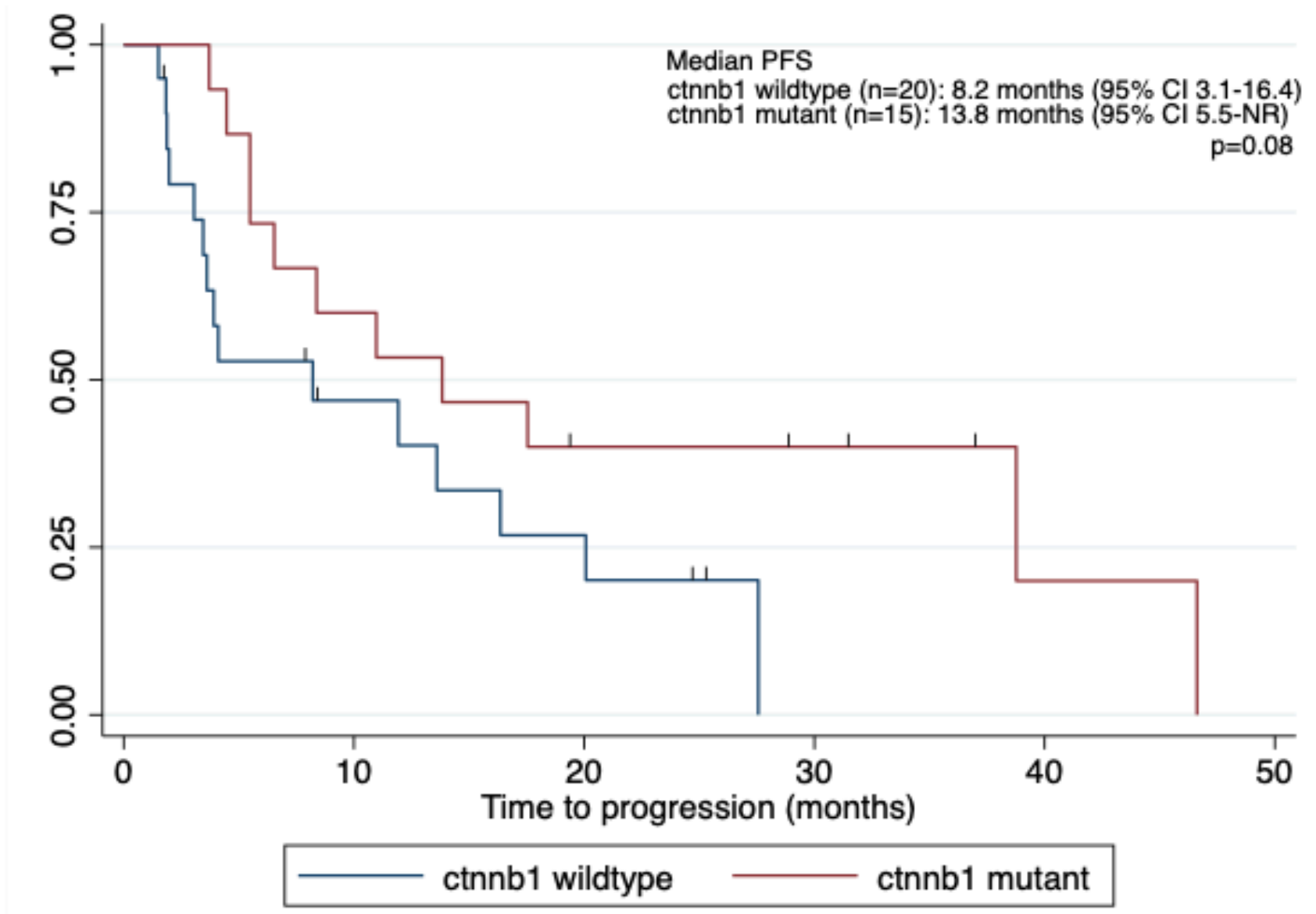

Number at risk

Control arm (ctnnb1 wildtype)	20	4	0	0
Control arm (ctnnb1 mutant)	15	5	1	0

b

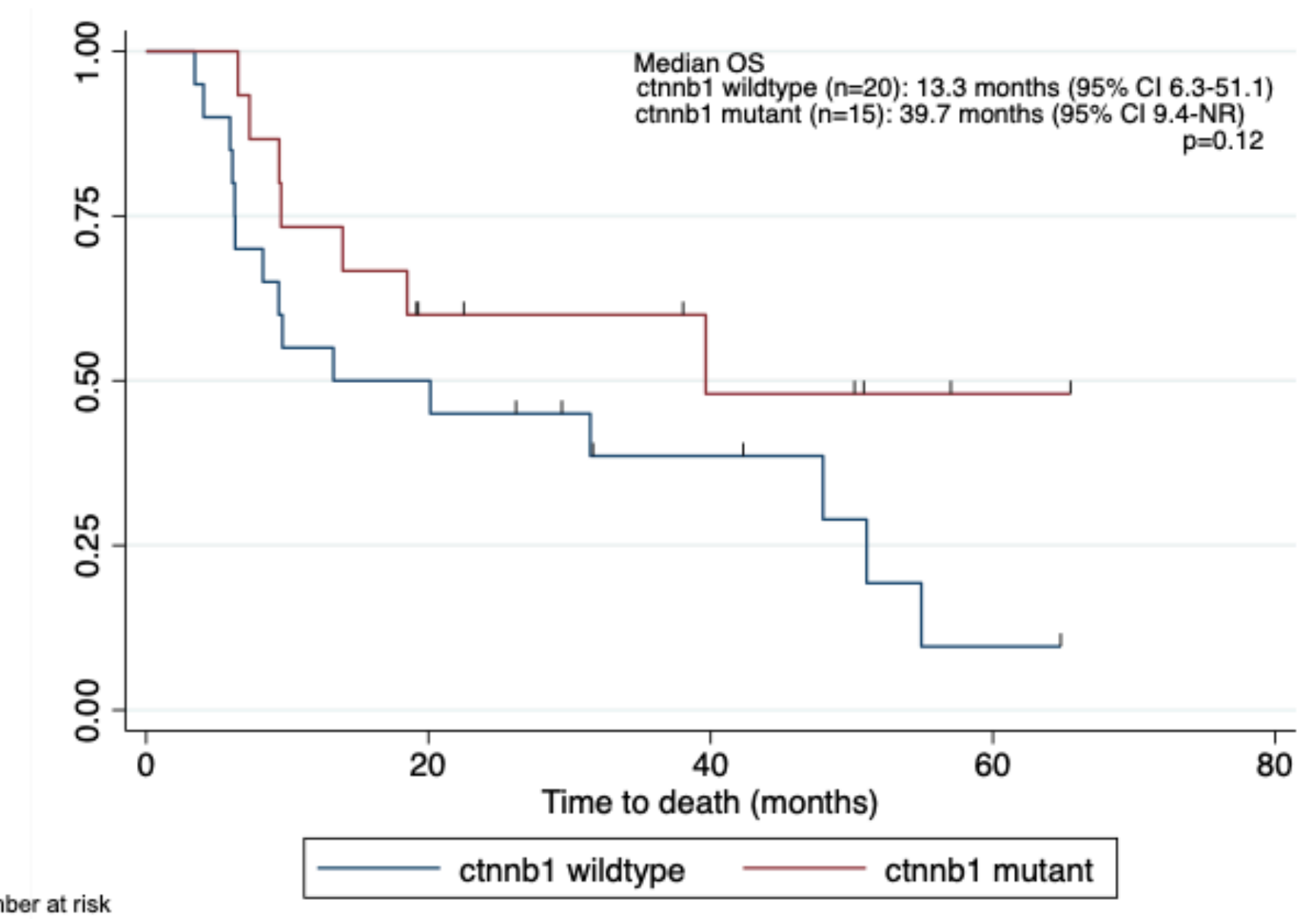

Number at risk

Control arm (ctnnb1 wildtype)	20	19	5	1	0
Control arm (ctnnb1 mutant)	15	7	4	1	0

Legend: a) PFS by *CTNNB1* status in the control arm b) OS by *CTNNB1* status in the control arm. NR = not reached. PFS = progression-free survival. OS = overall survival. The Kaplan-Meier estimator was used to estimate PFS And OS Kaplan-Meier curves. The Cox proportional hazards model was used to estimate the HR and the log-rank test was performed with two-sided p-values. Adjustments were not made for multiple comparisons.

Supplementary Figure 3 – Subgroup analyses for PFS and OS

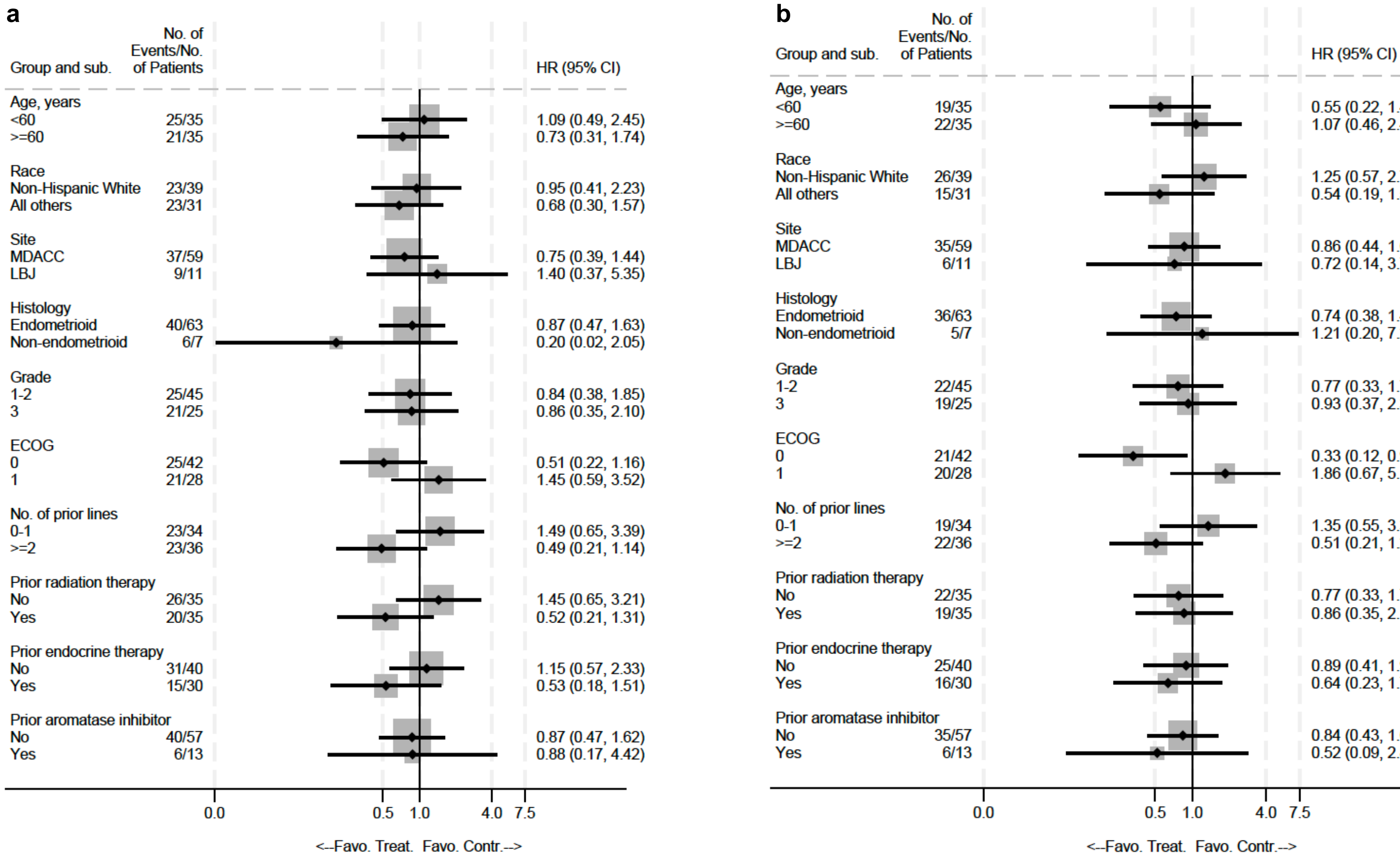

Legend: a) Subgroup analyses for PFS b) Subgroup analyses for OS. ECOG = Eastern Cooperative Oncology Group. LBJ = Lyndon B. Johnson Hospital. MDACC = MD Anderson Cancer Center. The Cox proportional hazards model was used to estimate the HR. Adjustments were not made for multiple comparisons. Source data are provided as a Source Data file.

A Phase II, Randomized Two-Arm Study of Everolimus and Letrozole, +/- Ribociclib (LEE011) in Patients with Advanced or Recurrent Endometrial Carcinoma

Sponsor: The University of Texas MD Anderson Cancer Center (MDACC)

MDACC Protocol Number: 2015-0961

Supporter: Novartis

Novartis Protocol Number: CLEE011XUS22T

Primary Site: The University of Texas, MD Anderson Cancer Center, Houston, TX

Study Principal Investigator
Pamela T. Soliman, MD, MPH
MD Anderson Cancer Center
Department of Gynecologic Oncology and Reproductive Medicine, Unit 1362
1155 Pressler Drive, CPB6.3244
Houston, TX 77030
Phone: (713) 745-2352
Fax: (713) 792-7586
Email: psoliman@mdanderson.org

Shannon Westin, MD, MPH
MD Anderson Cancer Center
Department of Gynecologic Oncology and Reproductive Medicine, Unit 1362
1155 Pressler Drive CPB6.3274
Houston, TX 77030
Phone: (713) 794-4314
Fax: (713) 792-7586
Email: swestin@mdanderson.org

Study Statistician
Ying Yuan, PhD
MD Anderson Cancer Center
Department of Biostatistics, Unit 1411
1515 Holcombe Blvd. FCT4.6034
Houston, TX 77030
Phone: (713) 563-4271
Fax: (713) 563-4243
Email:yyuan@mdanderson.org

Study Co-Investigators
Jeffrey How, MD
MD Anderson Cancer Center
Department of Gynecologic Oncology and Reproductive Medicine, Unit 1362
1155 Pressler Drive, CPB6.3231
Houston, TX 77030
Phone: (832) 207-0738
Fax: (713) 792-7586
Email: jahow@mdanderson.org

Pathology Collaborator
Barrett C. Lawson, MD
MD Anderson Cancer Center
Department of Pathology, Anatomical
1515 Holcombe Blvd.
Houston, TX 77030
Phone: (713) 563-9432
Fax: (713) 745-0789
Email: bclawson@mdanderson.org

Participating Site: The Harris Health System (LBJ), Houston, TX

Site Principal Investigator
Michaela Onstad Grinsfelder, MD
MD Anderson Cancer Center/The Harris Health System (LBJ)
Department of Gynecologic Oncology and Reproductive Medicine, Unit 1362
1155 Pressler Drive, CPB6.3554
Houston, TX 77030
Phone: (713) 745-7418
Fax: (713) 792-7586
Email: maonstad@mdanderson.org

TABLE OF CONTENTS

GLOSSARY OF TERMS **8**

1 INTRODUCTION **9**

1.1 ENDOMETRIAL CANCER 9
1.2 INVESTIGATIONAL AGENTS 11
1.3 STUDY RATIONALE AND PURPOSE 15

2 STUDY OBJECTIVES **20**

2.1 OVERVIEW 20
2.2 PRIMARY OBJECTIVE 20
2.3 SECONDARY OBJECTIVES 20
2.4 EXPLORATORY OBJECTIVE 21
2.5 PRIMARY ENDPOINT 21
2.6 SECONDARY ENDPOINTS 21

3 INVESTIGATIONAL PLAN **21**

3.1 OVERALL STUDY DESIGN 21
3.2 SCHEMA 25
3.3 PATIENT POPULATION 26
3.4 TREATMENTS 29
3.5 CORRELATIVE STUDIES 38

4 CONCOMITANT THERAPY **39**

4.1 INHIBITORS OF CYP3A4 AND/OR PGP 40
4.2 INDUCERS OF CYP3A4 AND/OR PGP 40

5 DISCONTINUATION, INTERRUPTIONS AND DOSE ADJUSTMENTS **40**

5.1 DISCONTINUATION OF TREATMENT 40
5.2 DOSE ADJUSTMENTS: 41

6 ASSESSMENTS **52**

6.1 HBV/HCV ASSESSMENTS 52
6.2 EFFICACY ASSESSMENTS 53

7 SAFETY ASSESSMENTS **58**

7.1 ADVERSE EVENTS 58
7.2 TREATMENT COMPLIANCE 59
7.3 SAFETY MONITORING 59

8 ALLOWANCES FOR REMOTE PROCEDURES **62**

9 PROTOCOL AMENDMENTS, OR CHANGES IN STUDY CONDUCT **62**

10 DATA MANAGEMENT **63**

10.1 DATA COLLECTION 63

11 STATISTICAL METHODS **63**

11.1 DEFINITION OF PRIMARY ENDPOINT 63
11.2 DEFINITION OF SECONDARY ENDPOINTS 64
11.3 ANALYTIC PLAN FOR PRIMARY OBJECTIVE 64
11.4 ANALYTIC PLAN FOR SECONDARY OBJECTIVES 64
11.5 DEFINITION OF POPULATIONS FOR ANALYSES 64
11.6 SAMPLE SIZE JUSTIFICATION 64
11.7 TOXICITY MONITORING 65

12 PROCEDURES AND INSTRUCTIONS 66

12.1 PUBLICATION OF RESULTS 66
12.2 DISCLOSURE AND CONFIDENTIALITY 66
12.3 DISCONTINUATION OF STUDY 66
12.4 ETHICS AND GOOD CLINICAL PRACTICE 66
12.5 INSTITUTIONAL REVIEW BOARD/INDEPENDENT ETHICS COMMITTEE 67
12.6 PROTOCOL REVIEW AND MONITORING 67
12.7 INFORMED CONSENT 67
12.8 DECLARATION OF HELSINKI 67

13 REFERENCES 69

APPENDIX A – GOG PERFORMANCE STATUS 73

APPENDIX B - LIST OF PROHIBITED MEDICATIONS AND PERMITTED MEDICATIONS TO BE USED WITH CAUTION DURING EVEROLIMUS + LETROZOLE + RIBOCICLIB 74

Figure 1. Chemical Structure of Everolimus.... 12

Figure 2. LEE011, and everolimus in MCF 7 xenograft.... 17

Figure 3. Triplet combination of LEE11, everolimus and fulvestrant in ZR and KPL -1 xenograft models 18

Figure 4. Proposed model of CTNNB1 mutation in endometrial endometrioid adenocarcinoma. ... 20

Figure 5. Safety Lead-In.... 25

Figure 6. Expansion 25

LIST OF ABBREVIATIONS

4E-BP1	4E-binding protein
Ab	Antibodies
ADR	Adverse Drug Reaction
AE	adverse event
ANC	Absolute neutrophil count
ALT/SGPT	alanine aminotransferase/glutamic pyruvic transaminase/Serum glutamic-pyruvic transaminase
AST/SGOT	aspartate aminotransferase/glutamic oxaloacetic transaminase/Serum glutamic-oxaloacetic transaminase
ATC	Anatomical Therapeutic Chemical classification system
AUC	Area under the plasma-concentration time curve
BAC	Bronchoalveolar carcinoma
BUN	Blood urea nitrogen
Cmax	Maximum plasma concentration
CR	Complete Response/Complete Remission
CRF	Case report/Record form
CRO	Contract Research Organization
CT	Computer tomography
CTC	Common toxicity criteria
CV	Coefficient of Variation
CYP3A4	CytochromeP450 3A4 isoenzyme
DNA	Deoxyribonucleic Acid
DS&E	Drug Safety and Epidemiology
DLT	Dose limiting toxicity
ECG	Electrocardiogram
EGF	Epidermal growth factor
EGFR	Epidermal growth factor receptor
eIF-4E	Eucariotic Initiation Factor 4E
EPR	Early progression rate
EU	European Union
FDG-PET	Fluorine-18-2-fluoro-Deoxy-D-Glucose Positron Emission Tomography
FKBP-12	FK506-binding protein 12
GF	Growth factor
HBV	Hepatitis B virus
HBcAb	Hepatitis B core antibodies
HBs Ab	Hepatitis B surface antibodies

HBsAg	Hepatitis B surface antigen
HCV	Hepatitis C virus
HDL	High-density lipoprotein
IB	Investigator's Brochure
ICH	International Conference on Harmonisation
IEC	Independent Ethics Committee
INR	International Normalized Ratio
IRB	Institutional Review Board
LDL	Low-density lypoproteins
LFTs	liver function tests
LLOQ	Lower limit of quantification
MAPK	Mitogen Activated Protein Kinase
mRNA	messenger Ribonucleic acid
MRT	Malignant rhabdoid tumors
mTOR	mammalian Target of Rapamycin
NIH/NCI	National Institutes of Health/National Cancer Institute
nM	nano-molar
NSCLC	Non-small cell lung cancer
OS	overall survival
P-AKT	phosphor-AKT
PCR	Polymerase Chain Reaction
PD	Pharmacodynamics
PET	Proton emission tomography
PFS	progression free survival
P-gp	P-glycoprotein
PI3K	Phosphoinositide 3-kinase
PK	Pharmacokinetics
PK/PD model	Pharmacokinetic/pharmacodynamic model
PT/PTT	prothrombin time
PTEN	Phosphatase and Tensin homolog deleted on chromosome 10
RBC	red blood cell count
REB	Research Ethics Board
RNA	Ribonucleic acid
RR	response rate
RT	Radiation therapy
S6K1	S6 kinase 1
SAE	serious adverse event

s.c.	subcutaneous
SCLC	Small cell lung cancer
STAT3	Signal Transducer and Activator of Transcription 3
TK	Tyrosine kinase
TSC2	Tuberous Sclerosis Complex 2
TUNNEL	Terminal deoxynucleotidyl transferase (TdT)-mediated dUTP-biotin Nick End Labeling
ULN	Upper limit of normal
VEGF	Vascular Endothelial Growth Factor
WBC	total white blood cell count
WHO	World Health Organization

GLOSSARY OF TERMS

Assessment	A procedure used to generate data required by the study
Baseline	For efficacy evaluations, the baseline assessment will be the last available assessment before or on the date of randomization.<br>For safety evaluations (i.e. laboratory assessments and vital signs), the baseline assessment will be the last available assessment before or on the start date of study treatment.<br>The value obtained at baseline assessments, referred to as "baseline value" will be used as reference for the patient.
Control drug	A study treatment used as a comparator to reduce assessment bias, preserve blinding of investigational drug, assess internal study validity, and/or evaluate comparative effects of the investigational drug
Dose level	The dose of drug given to the patient (total daily or weekly etc.)
Enrollment	Point/time of patient entry into the study; the point at which informed consent must be obtained (i.e. prior to starting any of the procedures described in the protocol)
Investigational drug	The study treatment whose properties are being tested in the study; this definition is consistent with US CFR 21 Section 312.3 and is synonymous with "investigational new drug."
Investigational treatment	Drug whose properties are being tested in the study as well as their associated placebo and active treatment controls (when applicable). This also includes approved drugs used outside of their indication/approved dosage, or that are tested in a fixed combination. Investigational treatment generally does not include other study treatments administered as concomitant background therapy required or allowed by the protocol when used in within approved indication/dosage
Medication number	A unique identifier on the label of each study treatment package which is linked to one of the treatment groups of a study
Other study treatment	Any drug administered to the patient as part of the required study procedures that was not included in the investigational treatment
Patient	A unique identifying number assigned to each patient/subject/healthy volunteer who enrolls in the study

Period	A subdivision of the study timeline; divides stages into smaller functional segments such as screening, baseline, titration, washout, etc.
Premature<br>patient withdrawal	Point/time when the patient exits from the study prior to the planned completion of all study treatment administration and/or assessments; at this time all study treatment administration is discontinued and no further assessments are planned, unless the patient will be followed for progression and/or survival
Stop study participation	Point/time at which the patient came in for a final evaluation visit or when study treatment was discontinued whichever is later
Study treatment	Includes any drug or combination of drugs in any study arm administered to the patient (subject) as part of the required study procedures, including placebo and active drug run-ins.<br>In specific examples, it is important to judge investigational treatment component relationship relative to a study treatment combination; study treatment in this case refers to the investigational and non-investigational treatments in combination.
Study treatment discontinuation	Point/time when patient permanently stops taking study treatment for any reason; may or may not also be the point/time of premature patient withdrawal
Supportive treatment	Refers to any treatment required by the exposure to a study treatment, e.g. premedication of vitamin supplementation and corticosteroid for pemetrexed disodium.
Variable	Identifier used in the data analysis; derived directly or indirectly from data collected using specified assessments at specified time points

1 INTRODUCTION

1.1 ENDOMETRIAL CANCER

1.1.1 EPIDEMIOLOGY

Endometrial cancer is the most common gynecologic malignancy in the United States. An estimated 46,470 women will have been diagnosed with uterine cancer in 2011, and it is estimated that 8,120 of these women will die of the disease (Siegel, et al. 2011). Endometrial adenocarcinoma is most frequently diagnosed in women between the ages of 50 and 65 years. Women have an overall lifetime risk of 2.53% (1 in 40) of developing endometrial cancer (Jemal, et al. 2010). Of the endometrial carcinomas, 80% are endometrioid adenocarcinomas and 15-20% are rarer subtypes: papillary serous, clear cell, mucinous, or mixed carcinomas (Prat, 2004). While the majority of women are diagnosed at an early stage and are often cured, patients with advanced or recurrent disease continue to have a poor long-term prognosis.

1.1.2 BIOLOGY

Based on epidemiologic, molecular, and prognostic factors, endometrial cancer can be subdivided broadly into two types. Type I carcinomas account for approximately 80% of cases and are classically of endometrioid histology. Type II carcinomas are often of non-endometrioid histology (serous and clear cell) and typically arise in a background of atrophic endometrium. These lesions appear to be unrelated to estrogen stimulation and have a worse prognosis.

Type I and type II lesions exhibit distinct molecular alterations. The most frequent genetic alteration associated with type I lesions is a loss of function of the tumor suppressor PTEN. In contrast, PTEN inactivation is uncommon in Type II lesions. PTEN loss of function can be seen in up to 83% of endometrioid carcinomas and 55% of precancerous lesions (Mutter, et al. 2000). Mutations in PTEN have been documented in endometrial hyperplasia with and without atypia and, thus, have been postulated to be an early event in the endometrial tumorigenesis process (Maxwell, et al. 1998). In addition to PTEN gene mutations, PTEN inactivation may also occur as a result of epigenetic silencing through promoter hypermethylation. PTEN most notably plays a role in the regulation of the phosphatidylinositol 3-kinase (PI3K)/AKT pathway by inhibiting the downstream phosphorylation of AKT. Recent studies have identified AKT as a potential mediator of tumorigenesis in endometrial cancer cell lines through the upregulation of signaling through mammalian target of rapamycin (mTOR) which is downstream of AKT and is involved in cellular proliferation and mRNA translation. Thus, the PI3K-AKT-mTOR pathway is a logical target in the treatment of endometrial cancer.

1.1.3 RATIONALE FOR CURRENT STUDY

Current treatment standards for advanced and recurrent endometrial cancer are limited to chemotherapy (none of which have an FDA-approved indication) with diminishing returns on use particularly in second or further lines of therapy. An extensive exploratory queue within the GOG identified 3 agents with significant activity, doxorubicin, paclitaxel and platinum (cisplatin and carboplatin). These three agents have been the focus of phase III investigation for more than 2 decades, settling with a recent non-inferiority trial demonstrating that paclitaxel and carboplatin is the de facto clinical standard. Response rates following this regimen, however, fall to 20% (or less in nearly all cytotoxic agents, including anthracyclines, such as, pegylated liposomal doxorubicin). Effective treatment options at this point are extremely limited.

Several classes of drugs have been developed that target specific aberrations in tumorigenic pathways for endometrial carcinoma. One promising target that we have investigated has been the mTOR pathway through the use of rapamycin analogs. Several "in class" agents have been developed and studied in endometrial cancer, including RAD001 (everolimus). In a phase II study conducted by our group of 28 patients with advanced or recurrent Type I endometrial carcinoma. RAD001 produced no complete or partial responses, but did demonstrate prolonged stable disease in 12 patients (43%) at 8 weeks evaluation and in 6 patients (21%) at 20 weeks evaluation. We recently completed a follow up study interrogating both the mTOR and the anti-estrogen pathway. This was a phase II study of everolimus and letrozole in a similar population of patients. A total of 38 patients were enrolled within an 18 month time period. Of the 35 evaluable patients, 15 (40%) had at least stable disease. More importantly, the overall response rate was 32% including 9 patients that had a complete response.

Based on our previous experience, our ability to accrue and completion of similar studies, as well as some new pre-clinical data we feel the current proposed study of ribociclib, everolimus and letrozole for the

treatment of recurrent or advanced endometrial cancer would be a rational follow up study. In breast cancer, ribociclib has been shown to have anti-tumor activity in HR-positive pre-clinical tumor xenograft models. In addition, ribociclib combined with letrozole has synergistic effects with sustained tumor control in PIK3CA-wildtype, ER-positive breast cancer models. There are ongoing trials with everolimus, ribociclib, and exemestane in breast cancer in which a dose has been established. Therefore, it makes sense to study this combination in recurrent endometrioid endometrial cancer which has a similar molecular profile. In addition, some of our pre-clinical work suggests that we may be able to identify a subgroup of women with recurrent endometrial cancer that may be more likely to respond

1.2 INVESTIGATIONAL AGENTS

1.2.1 EVEROLIMUS (RAD001)

Everolimus (Afinitor®/Votubia®; RAD001) is a signal transduction inhibitor (STI) targeting mTOR (mammalian target of rapamycin), or more specifically, mTORC1 (mammalian 'target of rapamycin' complex 1). mTOR is a key serine-threonine kinase playing a central role in the regulation of cell growth, proliferation and survival. The regulation of mTORC1 signaling is complex, being modulated by mitogens, growth factors, energy and nutrient availability. mTORC1 is an essential regulator of global protein synthesis downstream on the PI3K/AKT/mTOR pathway, which is dysregulated in the majority of human cancers (Boulay and Lane 2007). Consistent with the known activity of mTORC1, its inhibition by everolimus has been shown to reduce cell proliferation, glycolysis and angiogenesis in solid tumors in vivo, both through direct antitumor cell activity and inhibition of the tumor stromal compartment.

Everolimus is being investigated as an anticancer agent based on its potential to act:

- Directly on the tumor cells by inhibiting tumor cell growth and proliferation
- Indirectly by inhibiting angiogenesis leading to reduced tumor vascularity (via potent inhibition of tumor cell HIF-1 activity, VEGF production and VEGF-induced proliferation of endothelial cells). The role of angiogenesis in the maintenance of solid tumor growth is well established, and the mTOR pathway has been implicated in the regulation of tumor production of proangiogenic factors as well as modulation of VEGFR signaling in endothelial cells.

At weekly and daily schedules and at various doses explored, RAD001 is generally well tolerated. The most frequent adverse events (rash, mucositis, fatigue and headache) associated with everolimus therapy are manageable. Non-infectious pneumonitis has been reported with mTOR inhibitors but is commonly low-grade and reversible. Preclinical data and safety information is provided in the IB (everolimus IB).

See Table 1 and Figure 1 for more information.

Table 1. Everolimus – Drug Substance

Chemical name	(1R,9S,12S,15R,16E,18R,19R,21R,23S,24E,26E,28E,30S,32S,35R)-1,18-dihydroxy-12-{(1R)-2-[(1S,3R,4R)-4-(2-hydroxyethoxy)-3-methoxycyclohexyl]-1-methylethyl}-19,30-dimethoxy-15,17,21,23,29,35-hexamethyl-11,36-dioxa-4-aza-tricyclo[30.3.1.0^{4,9}]hexatriaconta-16,24, 26,28-tetraene-2,3,10,14,20-pentaone
International non-proprietary name	Everolimus

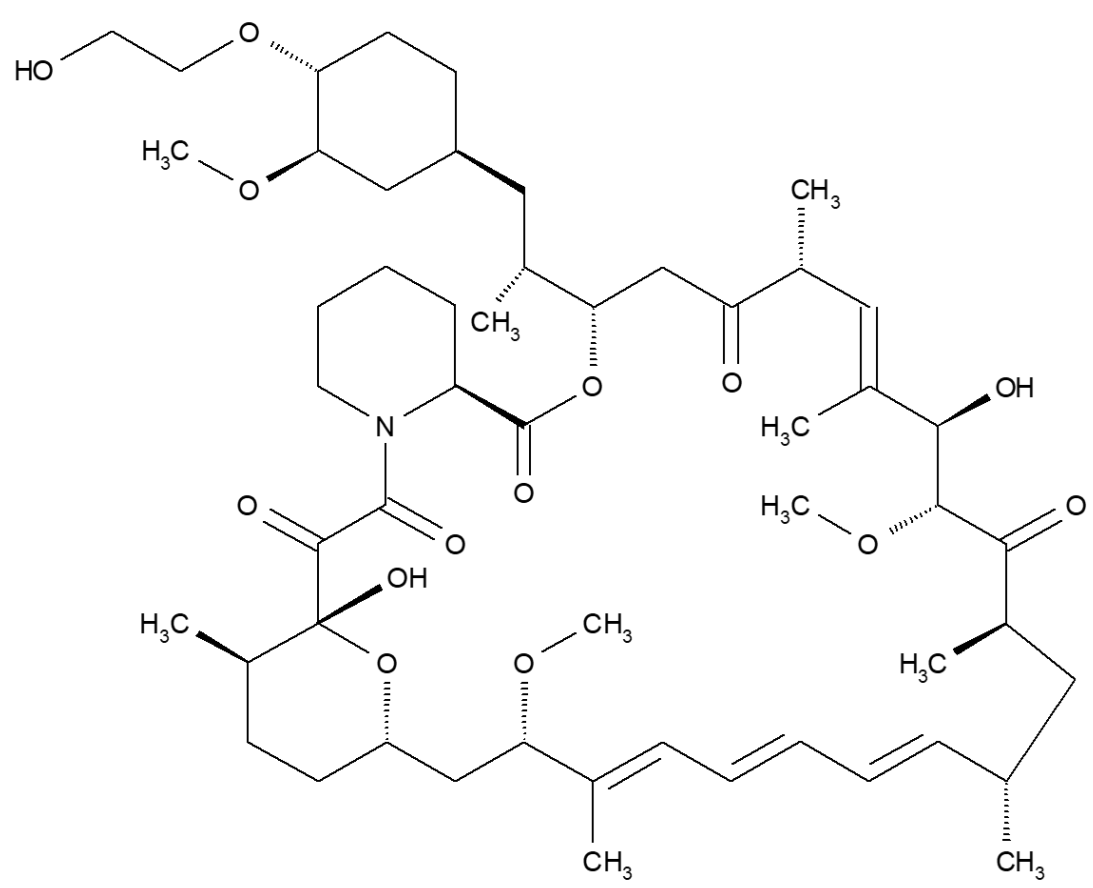

Figure 1. Chemical Structure of Everolimus.

1.2.1.1 MTOR PATHWAY AND MECHANISM OF ACTION

The target of everolimus, mTOR (mammalian target of rapamycin), is a serine-threonine kinase implicated in the PI3/AKT pathway and is known to be active in numerous neoplastic conditions.

The main known functions of mTOR include the following (Bjornsti and Houghton, 2004; Boulay and Lane, 2007):

2

mTOR functions as a sensor of mitogens, growth factors and energy and nutrient levels, facilitating cell-cycle progression from G1 to S phase in appropriate growth conditions.

- The PI3K-mTOR pathway itself is frequently activated in many human cancers, and oncogenic transformation may sensitize tumor cells to mTOR inhibitors.
- Through inactivating eukaryotic initiation factor 4E binding proteins (4E-BP1) and activating the 40S ribosomal S6 kinases (i.e., p70S6K1), mTOR regulates translation of important massages, including those encoding the HIF-1 proteins, c-myc, ornithine decarboxylase, and cyclin D1, as well as ribosomal proteins themselves.
- The activation of mTOR pathway leads to the increased production of pro-angiogenic factors (i.e., VEGF) in tumors and to tumor, endothelial and smooth muscle cell growth and proliferation.
- The regulation of mTOR signaling is complex and involves positive regulators, such as AKT that phosphorylate and inactivate negative regulators such as the Tuberous Sclerosis Complex (TSC1/TSC2).

1.2.1.2 CLINICAL EXPERIENCE

In oncology, everolimus has been in clinical development since 2002 for patients with various hematologic and non-hematologic malignancies as a single agent or in combination with antitumor agents. Please note that safety pharmacology and toxicology studies as well as some human pharmacology studies which have been conducted in support of the transplant indication, are described in the oncology IB due to the relevance of these data for the oncology indication.

Everolimus was approved by the United States (US) Food and Drug Administration (FDA) under the trade name Afinitor® ® for the treatment of patients with advanced renal cell carcinoma (RCC) after failure of treatment with sunitinib or sorafenib, and for the treatment of progressive neuroendocrine tumors of pancreatic origin (PNET) in patients with unresectable, locally advanced or metastatic disease. The European Commission approved Afinitor® ® for the treatment of patients with advanced RCC, whose disease has progressed on or after treatment with vascular endothelial growth factor-targeted therapy, and for the treatment of unresectable or metastatic, well- or moderately-differentiated neuroendocrine tumors (NET) of pancreatic origin in adults with progressive disease.

A randomized Phase II, double-blind, placebo-controlled study (the BOLERO-2 Study) demonstrated very significant improvements in treatment of HR + breast cancer that had recurred or progressed on letrozole or anastrozole. Response rate, progression-free survival (PFS), and clinical benefit rate were all significantly improved relative to exemestane (HR = 0.45; 95% CI: 0.38-0.54; p<.0001). Overall response rate (12.6% vs 1.7%; p<.0001) and clinical benefit rate (51.3% vs 26.4%;<.0001) were superior in the everolimus + exemestane arm versus exemestane + placebo. Analysis by central assessment showed a median progression –free survival of 11 months with everolimus versus 4.1 months with placebo (HR = 0.38; 95% CI:0.31 – 0.48; p<.0001) confirming the results of the primary PFS analysis (Piccart 2012, Baselga 2012).

1.2.1.3 PHARMACOKINETICS

Everolimus is rapidly absorbed with a median t-max of 1-2 hours. The steady-state AUC0-τ is dose-proportional over the dose range between 5 to 70 mg in the weekly regimen and 5 and 10 mg in the daily regimen. Steady-state was achieved within two weeks with the daily dosing regimen. Cmax is dose-proportional between 5 and 10 mg for both the weekly and daily regimens. At doses of 20 mg/week and higher, the increase in Cmax is less than dose proportional.

In healthy subjects, high fat meals reduced systemic exposure to everolimus 10 mg (as measured by AUC) by 22% and the peak plasma concentration Cmax by 54%. Light fat meals reduced AUC by 32% and Cmax by 42%. Food, however, had no apparent effect on the post absorption phase concentration-time profile.

The blood-to-plasma ratio of everolimus, which is concentration-dependent over the range of 5 to 5,000 ng/mL, is 17% to 73%. The amount of everolimus confined to the plasma is approximately 20% at blood concentrations observed in cancer patients given everolimus 10 mg/day. Plasma protein binding is approximately 74% both in healthy subjects and in patients with moderate hepatic impairment.

Everolimus is a substrate of CYP3A4 and a substrate and moderate inhibitor of PgP. Following oral administration, everolimus is the main circulating component in human blood and is considered to contribute the majority of the overall pharmacologic activity. No specific excretion studies have been undertaken in cancer patients; however, data available from the transplantation setting found the drug to be mainly eliminated through the feces.

There was a significant correlation between AUC0-τ and pre-dose trough concentration at steady-state on the daily regimen. The mean elimination half-life of everolimus is approximately 30 hours.

Please refer to Appendix B for examples of clinically relevant CYP3A inhibitors and inducers andfor more information on the concomitant use of CYP3A4 inhibitors/inducers and other medications.

More information on everolimus pharmacokinetics is provided in the everolimus IB.

1.2.1.4 PHARMACODYNAMICS

Pharmacokinetic/pharmacodynamic modeling based on inhibition of the biomarker p70S6 kinase 1 [S6K1] in peripheral blood mononuclear cells [PBMC]) suggests that 5-10 mg daily should be an adequate dose to produce a high-degree of sustained target inhibition ([Study C2101] / [Study 2102], Lane, et al 2003). Molecular changes in tumors were evaluated through serial biopsying before and during treatment. Biopsying on treatment took place in week 4 (pharmacokinetic steady-state). All patients underwent a 24-hour post-dose biopsy. Patients following the weekly regimen had a further biopsy on Day 4 or 5 of the same week. Molecular activity was measured by immunohistochemistry. In the absence of a reliable technique for measuring mTOR phosphorylation, the phosphorylation status of downstream markers S6 and eIF4G, for which reliable antibodies exist, was selected as reflecting the immediate pharmacodynamic effect of everolimus. Also measured were changes in the phosphorylation status of upstream AKT and the proliferation index Ki67. Fifty-five patients were treated and the results revealed a dose and schedule dependent inhibition of the mTOR pathway with a near complete inhibition of pS6 and pelF-4G at the 10 mg/day and 50 mg/wk schedules. In addition, pAKT was upregulated in 50% of the treated tumors. In the daily schedule, there was a correlation between everolimus plasma trough concentrations and inhibition of peIF4G and p4E-BP1. There was good concordance of mTOR pathway inhibition between skin and tumor. ([Study C2107], Tabernero, 2008).More information is provided in the everolimus IB

1.2.2 LETROZOLE (FEMARA)

Letrozole is a highly potent, orally active non-steroidal competitive inhibitor of the aromatase enzyme system. It effectively inhibits the conversion of androgens to estrogens both *in vitro* and *in vivo*. It is indicated both as first-line treatment of postmenopausal women with hormone receptor positive or hormone receptor unknown locally advanced or metastatic breast cancer as well as for the treatment of advanced breast cancer in postmenopausal women with disease progression following antiestrogen therapy.

Letrozole was initially studied at doses of 0.1mg to 5.0mg daily in six non-comparative Phase I/II trials in 181 postmenopausal estrogen/progesterone receptor positive or unknown advanced breast cancer patients previously treated with at least antiestrogen therapy. Patients had received other hormonal therapies and also may have received cytotoxic therapy. Eight (20%) of forty patients treated with letrozole 2.5mg daily in Phase I/II trials achieved objective tumor response (complete or partial response).

A randomized, double-blinded, multinational trial compared Letrozole 2.5mg with tamoxifen 20mg in 916 postmenopausal patients with locally advanced (Stage IIIB or loco-regional recurrence not amenable to treatment with surgery or radiation) or metastatic breast cancer. Time to progression (TTP) was the primary endpoint of the trial. Letrozole was superior to tamoxifen in TTP and rate of objective tumor response.

Letrozole has been investigated to a limited degree in patients with advanced/recurrent endometrial cancer. In one study of 32 patients not previously exposed to chemotherapy (28 evaluable) 1 CR and 2 PR's were recorded (Ma, et al. 2004). The median PFS and OS was 3.9 and 8.8 months, respectively.

1.2.3 Ribociclib (LEE011)

In the mammalian cell cycle, entry into S phase is achieved by cyclin-dependent kinases 4 and 6 (CDK4/6). Ribociclib (formerly LEE011) is an orally bioavailable, highly selective small molecule inhibitor of CDK4/6 that induces G1 arrest at sub-micromolar concentrations in a variety of retinoblastoma protein (pRb)-positive cancer cells *in vitro*. Ribociclib has proven efficacious when combined with other targeted therapies *in vitro* and *in vivo* in cancers driven by a variety of oncogenic signaling pathways. Ribociclib may therefore be an effective anti-cancer agent in a variety of pRb-positive human neoplasms, especially in those that contain an activated CDK4/6-pRb pathway. Ribociclib is currently being developed in phase III clinical trials for the treatment of hormone receptor positive (HR+) breast cancer patients; several other phase I or II clinical studies are being conducted and presented in the investigator brochure.

Preclinical Studies in combination with everolimus

Ribociclib, when combined with the mTOR inhibitor, everolimus (RAD001; Afinitor), induced synergistic growth inhibitions in multiple tumor models, including cell lines derived from MCL, ER+ breast cancer, and MRTs (RD-2012-50383). The combination of ribociclib with the PI3K inhibitor, alpelisib (BYL719), showed similar synergistic effects in ER+ breast cancer, neuroblastoma and colorectal cancer cell lines (RD-2013-50144).

1.3 STUDY RATIONALE AND PURPOSE

1.3.1 RATIONALE FOR USE OF RAD001 IN ENDOMETRIAL CANCER

Given the frequency of PTEN loss of function and other aberrations of the PI3K/AKT/mTOR pathway in Type I endometrial tumors, the discovery of rapamycin and its analogs, which target and inhibit mTOR, have drawn interest as potential therapeutic agents in endometrial cancer. Preclinical data using RAD001 and other mTOR inhibitors in endometrial cancer have been compelling. In a preclinical, PTEN heterozygous transgenic mouse model, RAD001 decreased the progression of endometrial hyperplasia by increasing the rate of apoptosis and decreasing cell proliferation (Milam, et al. 2007). The decrease in phospho-p70 S6 kinase in the treated group compared to the untreated group also suggested a target specific down regulation of the mTOR pathway with the treatment of RAD001.

In endometrial cancer cell lines, rapamycin demonstrated a growth-inhibitory effect through induction of cell cycle arrest (Zhou, et al. 2003). This effect, however, was independent of PTEN status because PTEN-positive cells were as sensitive to rapamycin as PTEN-null cells. This suggested that rapamycin may be effective against a broad range of endometrial cancers. In addition, rapamycin inhibited phosphorylation of downstream targets of mTOR (S6K and 4E-BP1). In a mouse model, tumors from PTEN deficient mice had elevated levels of phosphorylated AKT and S6K (Podsypanina, et al. 2001). In this model, inactivation of mTOR using an mTOR inhibitor reduced neoplastic proliferation, tumor size, and S6K activity, but did not alter the status of AKT.

Our group has previously found a relatively high rate of activated mTOR and its downstream effector protein, S6 kinase in a series of endometrial cancers. These data support the current investigations of mTOR inhibition for the treatment of patients with this disease. Future studies will further evaluate markers in this pathway and possibly identify molecular factors that may predict response to therapy.

Clinical trials of mTOR inhibitors in various cancers are in progress. CCI-779, an intravenous analog of rapamycin, has been evaluated in a phase II trial in patients with metastatic or recurrent endometrial cancer. In this study there was a 25% partial response rate (median duration 5.1 months) and a 57% stable

disease rate (median duration 8.7 months). None of these patients received prior chemotherapy (Oza, et al. 2006).

As described above we completed a phase II study of 28 patients with advanced or recurrent Type I endometrial carcinoma who were treated the rapamycin analog RAD001 (Slomovitz, et al. 2010). While no complete or partial responses were produced, treatment with RAD001 alone did result in prolonged stable disease in 12 patients (43%) at 8 weeks evaluation and in 6 patients (21%) at 20 weeks evaluation. The median duration of stable disease observed was 4 cycles (months). Immunohistochemistry translational endpoints demonstrated 76% overexpression of mTOR and 41% overexpression of phospho-mTOR. No relationship of CBR was seen with PTEN mutation (66% of samples).

As expected based on studies of other targeted agents in the setting of recurrent endometrial cancer, measurable clinical responses were uncommon; however, the stable disease rate seen in this study was promising. Although toxicity (mostly hyperlipidemia) has been seen, clinically significant side effects were not problematic. In a preliminary analysis of translational endpoints and response, patients who had tumors harboring PTEN mutations were more likely to experience a clinical benefit (i.e., stable disease) than patients whose tumors had wild-type PTEN. We believe that this single agent activity in pretreated patients suggest that RAD001 will have clinical applications in the treatment of this disease. However, we felt that combination therapies would be needed to improve response rates.

1.3.2 RATIONALE FOR COMBINATION OF RAD001 WITH LETROZOLE IN ENDOMETRIAL CANCER

The combination of RAD001 and letrozole was first evaluated as part of a neoadjuvant breast cancer trial (Baselga, et al. 2009). The biologic rationale for the combination arose from preclinical observations that treatment with RAD001 caused reduced progesterone receptor levels resulting in loss of the antiproliferative effects associated with progesterone. Addition of aromatase inhibitors was suggested to counter the pro-proliferative effects of estrogen by reducing serum estrogen levels. In this phase II trial, RAD001 was found to significantly increase the efficacy of letrozole (ORR 68%) in patients with estrogen receptor-positive breast cancer compared to letrozole alone (ORR 59%). Furthermore, the safety profile of the combination was consistent with historical results of RAD001 used as monotherapy. The frequent expression of hormonal receptors in endometrial cancer, particularly in low grade lesions, was felt to be another setting in which this combination therapy may be effective.

To answer this question, a single-arm, open-label phase II trial of RAD001 (10 mg daily) in combination with letrozole (2.5 mg daily) was initiated. This regimen was given to 35 patients and demonstrated a clinical benefit rate of 48.6% and an overall response rate of 20.0% (including 4 complete responses). The median duration of response was 8 months (range, 3-17+). While these results are still preliminary and patient follow-up continues, the responses observed in this trial show significant promise to combined therapy with RAD001 and letrozole. Furthermore, this combination was well tolerated with the most common grade 3 toxicities being: fatigue (n=3), diarrhea (n=2), and thrombocytopenia (n=2). Interestingly, as mentioned above several patients (n=9) were either on metformin upon study entry for management of diabetes or metformin was added during the trial following the development of hyperglycemia. These patients who were treated with metformin while on trial were noted to have an increased clinical benefit rate (77.8%) compared to metformin non-users (CBR 38.5%). Of the 4 complete responses observed, 3 have been in patients also treated with metformin. Based on the

preliminary findings of this phase II trial with RAD001 and letrozole, we believe that the addition of metformin to this regimen may provide some added benefit.

1.3.3 RATIONALE FOR COMBINATION OF RAD001, LETROZOLE, AND RIBOCICLIB (LEE011) IN ENDOMETRIAL CANCER

In breast cancer, ribociclib has been shown to have anti-tumor activity in HR-positive pre-clinical tumor xenograft models. In addition, ribociclib combined with letrozole has synergistic effects with sustained tumor control in PIK3CA-wildtype, ER-positive breast cancer models. There are ongoing trials with everolimus, ribociclib, and exemestane in breast cancer in which a dose has been established. Therefore, it makes sense to study this combination in recurrent endometrioid endometrial cancer which has a similar molecular profile.

The effect of combining ribociclib with the mTORC1-inhibitor RAD001 (everolimus; Afinitor®) in the ER+, PIK3CA-mutant MCF-7 xenograft model was examined. As demonstrated in the figure below single agent treatments with ribociclib and RAD001 resulted in modest tumor regressions of -9% and -21%, respectively. When ribociclib was combined with RAD001 tumor regression increased to -49%. In addition, the triplet combination of ribociclib plus everolimus plus the endocrine therapy, fulvestrant, was evaluated in the ER+, PIK3CA-mutant KPL-1 and PTEN-null XR751 xenograft models. The triplet combination demonstrated superior tumor regression compared to single agent LEE011 or LEE011 plus fulvestrant (see Fig below) or compared to single agent everolimus or everolimus combined with fulvestrant. These data suggest that combining inhibitors that target the mTOR pathway with LEE011 may be an effective therapeutic strategy in ER+/PIK3CA-mutant tumors.

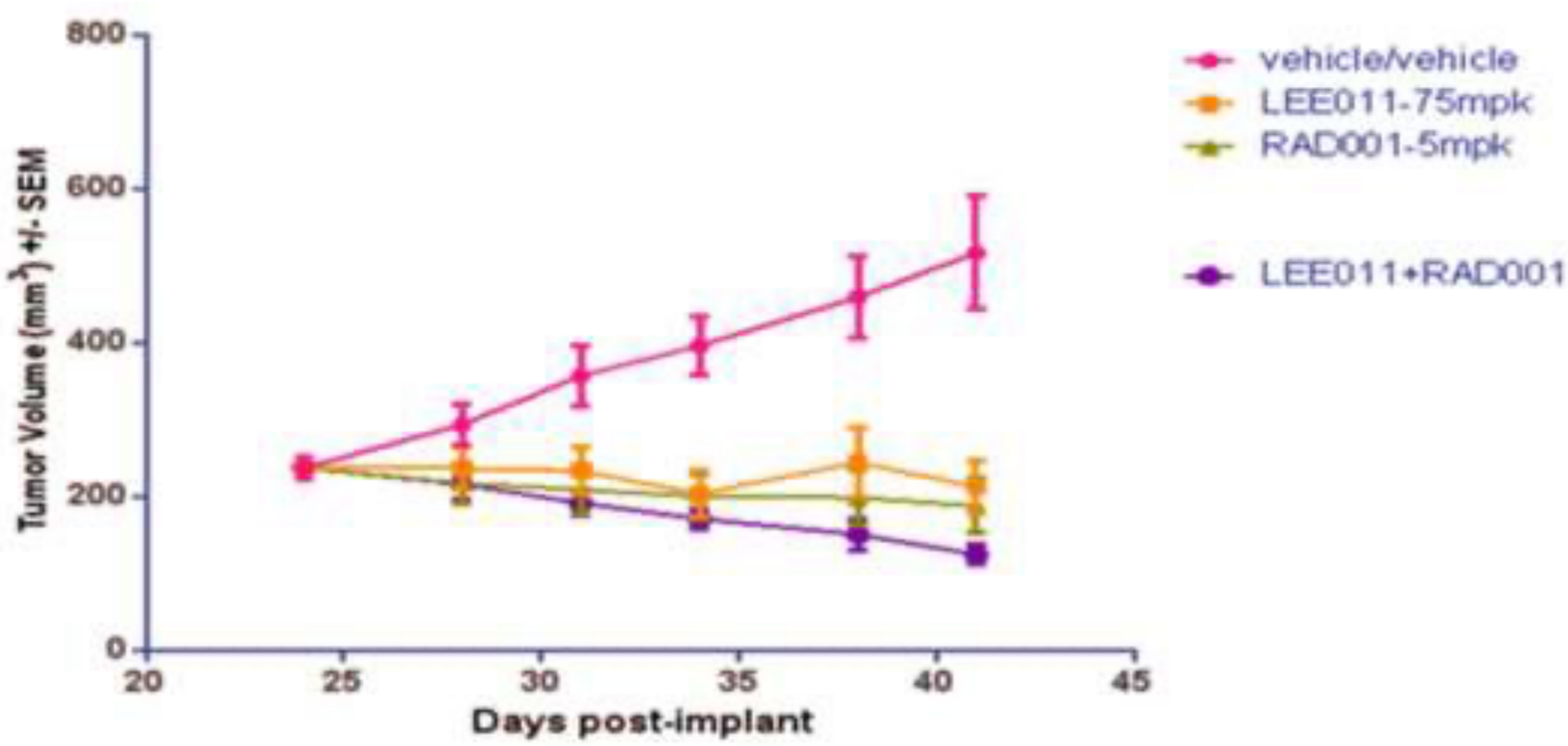

Figure 2. LEE011, and everolimus in MCF 7 xenograft

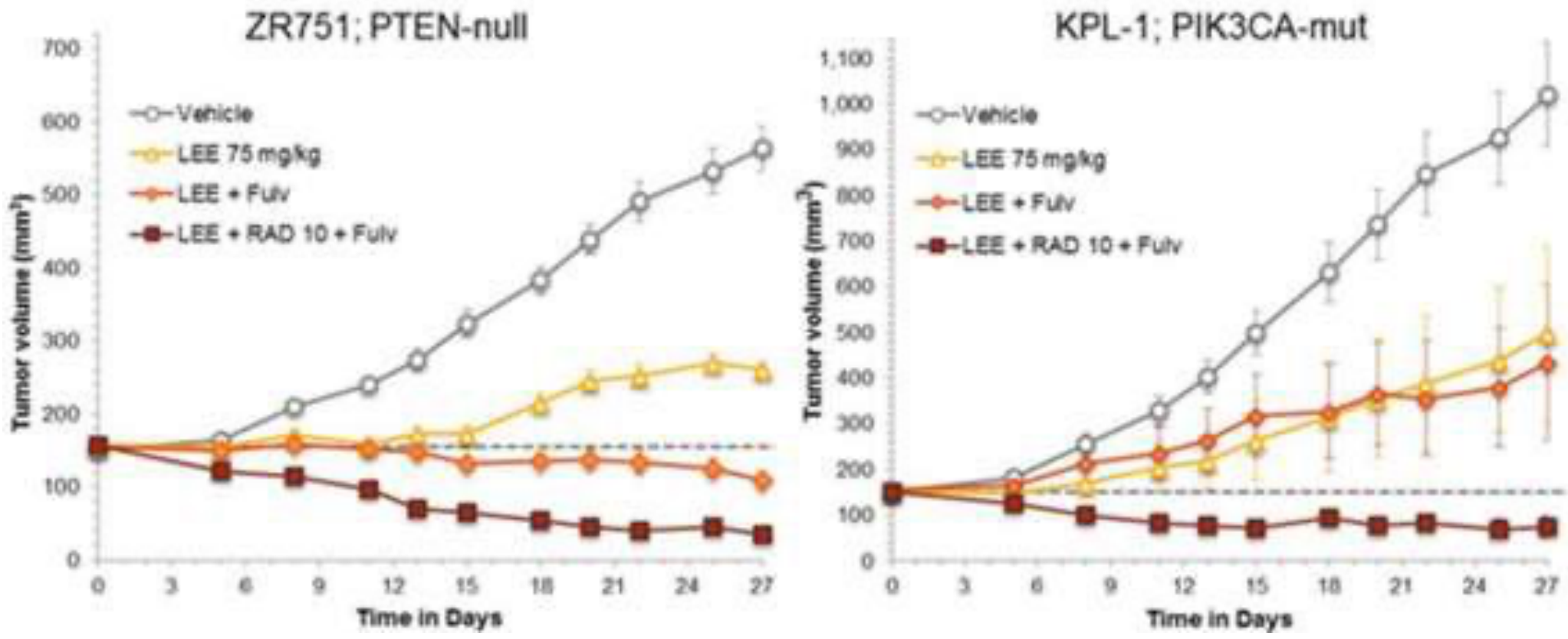

Figure 3. Triplet combination of LEE11, everolimus and fulvestrant in ZR and KPL -1 xenograft models

Cyclin-dependent kinase (CDK) 4/6 inhibitors have shown clinical activity in combination with endocrine therapy and could potentially overcome phosphatidylinositol 3-kinase (PI3K)/mammalian target of rapamycin (mTOR) inhibitor resistance by acting downstream of the PI3K pathway. In an ongoing Phase Ib/II study, postmenopausal women with anastrozole- or letrozole-resistant HR+/HER2– aBC received escalating doses of the CDK4/6 inhibitor ribociclib (LEE011 [LEE]; once daily [QD], days 1–21 of 28-day cycles) with EVE (1–5 mg QD) and EXE (25 mg QD). Everolimus (EVE; 10 mg) and exemestane (EXE; 25 mg) doublet therapy improved progression-free survival in women with hormone receptor-positive (HR+)/ HER2– advanced breast cancer (aBC; BOLERO-2), but was associated with significant mucositis as well as eventual disease progression.

At the cut-off date March 2, 2015, 84 patients (pts) had been treated; presented here are results from the 70 pts in the triplet arm treated with LEE (200–350 mg), EVE (most at 2.5 mg), and EXE. The median number of prior regimens was 5, and 18 (25.7%) pts had received prior PI3K/AKT/mTOR or CDK4/6 inhibitors for metastatic disease. Grade 3/4 treatment-related adverse events (AEs; ≥5% pts) were neutropenia (45.7%), leukopenia (8.6%), and thrombocytopenia (5.7%). Two (2.9%) pts discontinued due to AEs. Grade 3 dose-limiting toxicities were reported in 6 pts treated with LEE (300 mg) and EVE (2.5 mg): increased alanine aminotransferase/aspartate aminotransferase (2 pts), febrile neutropenia and hypophosphatemia (1 pt), oral mucositis (1 pt), rash and thrombocytopenia (1 pt), and thrombocytopenia with bleeding (1 pt). The recommended Phase II dose will be reported at the meeting. Among 55 pts evaluable for best overall response, there was 1 (1.8%) complete response (CR), 2 (3.6%) confirmed and 3 (5.5%) unconfirmed partial responses (PR), 7 (12.7%) non-CR, non-progressive disease (NCRNPD), and 26 (47.3%) stable disease (SD). Disease control rate (CR+PR+SD+NCRNPD) was 70.9%. One pt received treatment for ≥14 months, and 23 (32.9%) pts for ≥4 months. There was a trend towards longer duration of treatment in theCCND1 amplified group (n=10; median 166 days) than in the non-amplified group (n=22; median 60 days). A retrospective analysis of BOLERO-2 showed no differential effect ofCCND1 amplification on PFS.

In conclusion, triplet combination of endocrine therapy with mTOR and CDK4/6 inhibition is feasible, permits lower dosing of EVE (resulting in better tolerability), and shows encouraging signs of clinical activity, including in some pts with prior exposure to PI3K/AKT/mTOR or CDK4/6 inhibitors. Duration of treatment was longer in pts with cyclin D amplification, possibly due to inclusion of a CDK4/6

inhibitor. These results suggest that that triplet therapy might be beneficial for pts who progress on doublets or who have cyclin D amplification.

1.3.4 BACKGROUND AND RATIONALE FOR CORRELATIVE STUDIES

We recently completed an analysis of 66 endometrial cancer patients with advanced/recurrent disease who have had clinical next generation sequencing performed (n=31 pure endometrioid; n=35 non-endometrioid). None of the patients had an *RB1* mutation. Twenty-six per cent of the advanced/recurrent endometrioid patients had *CTNNB1* mutation; this mutation is not found in any of the non-endometrioid patients. From our recent analysis of TCGA-derived endometrial carcinoma data, we have shown that endometrioid-type endometrial carcinoma can be sub-divided into 4 different clusters. Cluster 2 is characterized by low grade (grades 1 or 2), low stage (stages I and II), and a preponderance of *CTNNB1* mutations. Interestingly, despite the fact that these patients have low grade and low stage at the time of diagnosis, their survival is significantly worse than that of patients in Cluster 1 who have low grade, low stage carcinomas characterized by higher expression of estrogen and progesterone receptors and estrogen-induced genes. *CTNNB1* mutation is classically associated with activation of the WNT signaling pathway. High expression of *Cyclin D1* and *WNT5A*, two genes known to be induced by *CTNNB1* mutation and WNT pathway activation, is also associated with decreased survival in this patient cohort. These results have been summarized in a recent publication. Cyclin D1 protein interacts with CDK4/6 to promote progression through the cell cycle. **We hypothesize that the patients with carcinomas with *CTNNB1* mutation will have higher expression of *Cyclin D1* and therefore be more responsive to treatment with the combination of Letrozole, Everolimus and LEE011 (**Figure 4**).**

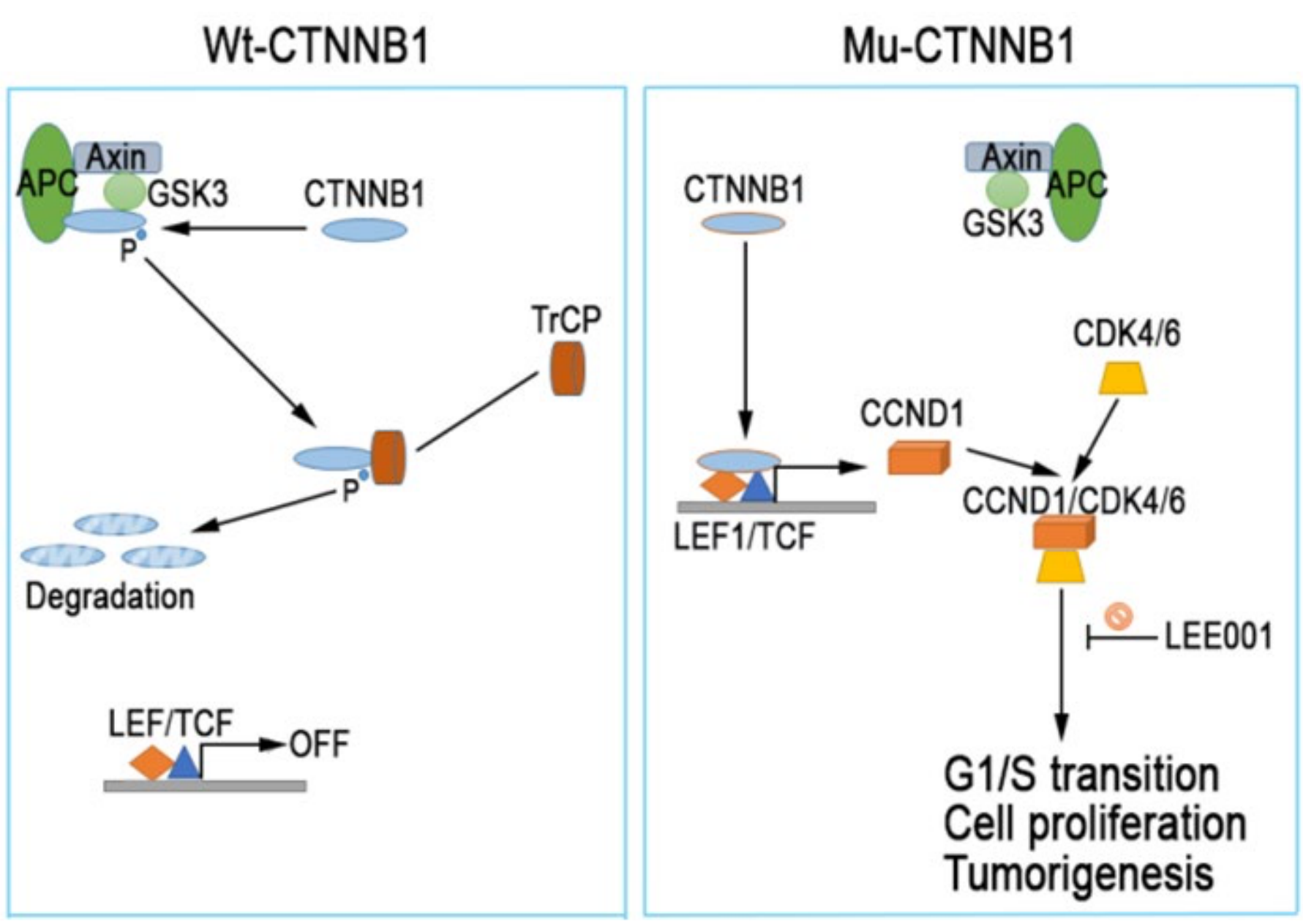

Figure 4. Proposed model of CTNNB1 mutation in endometrial endometrioid adenocarcinoma.

Activation of the CCND1/CDK4/6 complex in CTNNB1 mutated cells drives cell proliferation signals. These mutated cells should be vulnerable to LEE001, a CDK4/6 inhibitor.

2 STUDY OBJECTIVES

2.1 OVERVIEW

This is an open-label, phase II, randomized trial to evaluate the clinical efficacy of the combination of everolimus, letrozole, and ribociclib (LEE011) compared to everolimus and letrozole in the treatment of advanced or recurrent endometrial carcinoma. Pre-clinical data has demonstrated that the combination of letrozole and everolimus produces a decrease in endometrial cancer cell proliferation that is greater than either agent alone. This may be the result of targeting different pro-proliferative pathways to produce an additive effect. As patients with recurrent endometrial cancer often have limited effective treatment options, development and evaluation of novel therapies is needed. We hypothesize that addition of Ribociclib (LEE011) to everolimus and letrozole will be more effective in the treatment of patients with advanced or recurrent endometrial carcinoma than everolimus and letrozole alone.

This study is a multi-center open-label, phase II, randomized two-arm trial to evaluate the clinical efficacy of the combination of Ribociclib (LEE011), Everolimus (RAD001), and letrozole in the treatment of advanced or recurrent endometrial carcinoma. Eligible patients at MD Anderson Cancer Center (MDACC) and Harris Health System (LBJ) will be enrolled.

Throughout this protocol, references are made to the "**study Principal Investigator" (PI)**, "**site PI**," or "**investigator**." "Study PI" refers to either the study PI or the study Co-PIs as noted on page 1; "site PI" refers to the PI at LBJ as noted on page 2; and "investigator" refers to any investigator who has been delegated as such at any site.

The **sponsor** for this study is the MDACC IND office, and Novartis is a **supporter**.

2.2 PRIMARY OBJECTIVE

To determine if the addition of ribociclib (LEE011) to everolimus and letrozole improves progression free survival in patients with advanced or recurrent endometrial carcinoma.

2.3 SECONDARY OBJECTIVES

1. To determine clinical benefit and the median duration of progression-free survival (PFS) and overall survival (OS) in patients with advanced or recurrent endometrial carcinoma treated with ribociclib (LEE011), everolimus and letrozole versus everolimus and letrozole alone.
2. To determine the frequency and severity of toxicities associated with ribociclib (LEE011), everolimus, and letrozole in this cohort of patients.
3. To determine the presence of a *CTNNB1* mutation is associated with response to ribociclib (LEE011), everolimus, and letrozole.
4. To determine if liquid biopsies at baseline and 8 weeks correlate with presence of a tissue mutation or response to therapy.

2.4 EXPLORATORY OBJECTIVE

To examine pharmacodynamic effects of ribociclib in patients with advanced or recurrent endometrial carcinoma.

2.5 PRIMARY ENDPOINT

Progression free survival (PFS) is defined as the time from the date of randomization to progression or death due to any causes, whichever is earlier.

2.6 SECONDARY ENDPOINTS

1. Clinical benefit rate (CBR), defined as the rate of complete response + partial response + stable disease based on RECIST v1.1.
2. Overall survival (OS) is defined as the time from the date of randomization to death.
3. Frequency and severity of toxicities as assessed by CTCAE v4.0.
4. Correlative endpoints: Binary outcomes (present vs. absent) of *CTNNB1* or other gene mutations in the most recent tumor biopsy using the Solid Tumor Genomic Assay will be correlated with response to Ribociclib (LEE011), everolimus (RAD001), and letrozole. Biomarkers (PTEN, mTOR, AKT, p70S6K, and ERK1/2) will be also evaluated in pre-treatment samples. Correlation of liquid biopsy results with molecular tissue evaluation at baseline and response to therapy.

3 INVESTIGATIONAL PLAN

3.1 OVERALL STUDY DESIGN

This is an open-label, Phase II, randomized trial comparing ribociclib, everolimus, and letrozole versus everolimus and letrozole (with a safety lead-in of 6 patients to confirm doses of everolimus, letrozole, and ribociclib) to evaluate the clinical efficacy of the addition of ribociclib to everolimus and letrozole in advanced or recurrent endometrial carcinoma. After identification of a safe dose of ribociclib, everolimus, and letrozole in the safety lead in, patients will be randomized 1:1 to the 2 arms. Patients with stable disease, partial response, or complete response after 2 cycles will continue therapy until disease progression or inability to tolerate side effects. We will enroll a total of 35 evaluable patients per arm at a rate of 2 patients per month. Due to screen failures and lost to follow ups we will enroll 99 patients to have 35 patients evaluable per arm.

3.1.1 SAFETY LEAD-IN AND RATIONALE FOR CONTINUOUS DOSING OF RIBOCICLIB

While many of the early studies in ribociclib have used an intermittent dosing for ribociclib both single agent (600 mg 3 weeks on/ 1 week off) and in combination (i.e. 300mg daily for 3 weeks on/one week off when used in combination with everolimus 2.5 mg and examestane), there is published data that the combination of everolimus 10 mg po daily and letrozole 2.5 mg po daily is active in the treatment of endometrial cancer (Slomovitz, JCO). When combined with ribociclib the doses of both everolimus and ribociclib are given at a reduced dose as they are both CYP3A4 inducers. In the current study we are hoping to show that the addition of ribociclib may add benefit to the already proven combination of everolimus and letrozole. Ideally we would like a steady state of dosing for the everolimus to ensure patient are getting the known benefit of everolimus. If the ribociclib is given intermittently, there is concern that the everolimus may be under therapeutic dose during the ribociclib off week. The doses

were chosen based on preclinical studies and early doses found to be safe in the ongoing Phase I/II study in patients with breast cancer. (A Phase I/II, Single Arm, Open-label Study of Ribociclib in Combination With Everolimus + Exemestane in the Treatment of Men and Postmenopausal Women With HR+, HER2- Locally Advanced or Metastatic Breast Cancer Following Progression on a CDK 4/6 Inhibitor, please see link below).

The safety lead in will allow us to explore the safety of continuous dosing prior to expanding to the majority of patients on trial. A review of safety will occur after enrollment of the first 6 evaluable patients have completed at least one cycle (28 days) of treatment. The starting dose for the safety lead-in is outlined in Table 2.

Table 2. The starting dose for Safety Lead-in

Drug	Ribociclib	Everolimus	Letrozole
Dose and route (fasting 2 hours before and after dose administration)	250 mg PO	2.5 mg PO	2.5 mg PO
Frequency	Daily for 28 days	Daily for 28 days	Daily for 28 days

Within the safety lead-in, patients who do not have a dose limiting toxicity (DLT) and do not complete one cycle of treatment will be replaced. All patients that complete any treatment will be considered evaluable for a DLT unless they withdraw from the study for reasons other than toxicity. If ≤1 patient out of 6 experience a DLT in cycle 1 (including treatment delay for cycle 2 of greater than 2 weeks due to toxicity), then the regimen will be deemed safe for administration in the phase II study.

If 2 or more of 6 patients experience a DLT, then a dose reduction by one level will be considered as outlined in Table 3A and 6 additional patients will be enrolled at the -1 dose level.

Table 3A. The -1 dose level reduction for Safety Lead-in

Drug	Ribociclib	Everolimus	Letrozole
Dose and route<br>(fasting 2 hours before and after dose administration)	200 mg PO	2.5 mg PO	2.5 mg PO
Frequency	Daily for 28 days	Daily for 28 days	Daily for 28 days

If ≤1 patient out of 6 experience a DLT in cycle 1 (including treatment delays for cycle 2 of greater than 2 weeks due to toxicity), then this regimen will be deemed safe for administration in the phase II study. If 2 or more of 6 patients have a DLT at the -1 dose level, we will consider de-escalating and 6 additional patients will be enrolled at the -2 dose level. If dose level -1 is considered tolerable, we will add an additional dose level -1A to increase the dose of everolimus and determine if this continues to be tolerable (see Table 3B).

The rationale for adding this dose level -1A is that numerous studies done with the combination of everolimus and letrozole in endometrial cancer have been at the dose 10 mg and 2.5 mg respectively, and have shown excellent results. Because both ribociclib and everolimus are metabolized by CYP3A4, they

are given at a lower dose. PK data is available through Novartis showing that at a dose of ribociclib 250 mg and everolimus 2.5 mg, the everolimus is in the therapeutic window. At this time PK data is not available for ribociclib 200 mg and everolimus 2.5 mg dose level -1). To ensure that we are in the target window for everolimus (a drug known to be effective in the population) we will add dose level -1A to test for tolerability.

Table 3B. The -1A dose level reduction for Safety Lead-in

Drug	Ribociclib	Everolimus	Letrozole
Dose and route (fasting 2 hours before and after dose administration)	200 mg PO	5 mg PO	2.5 mg PO
Frequency	Daily for 28 days	Daily for 28 days	Daily for 28 days

If ≤1 patient out of 6 experience a DLT in cycle 1 (including treatment delays for cycle 2 of greater than 2 weeks due to toxicity), then this regimen will be deemed safe for administration in the phase II study. If 2 or more of 6 patients have a DLT at the -1A dose level, we will consider expanding at dose level -1 which has been shown to be tolerable.

3.1.2 DEFINITION OF DLT FOR SAFETY LEAD IN

A Dose Limiting Toxicity (DLT) is defined as either hematologic or non-hematologic toxicity [assessed in accordance with the current version of NCI Common Terminology Criteria, CTCAE], occurring during cycle 1 of therapy, which causes any of the following:

Table 4. Criteria for Dose Limiting Toxicities

Toxicity	DLT Criteria
Hematology	CTCAE grade 4 neutropenia lasting more than 7 consecutive days
	CTCAE grade 4 thrombocytopenia
	CTCAE grade 3 thrombocytopenia with bleeding
	CTCAE grade 3 or 4 febrile neutropenia
ECG QT Interval	QTc interval ≥ 501 ms on at least 2 separate ECGs
Cardiac	Cardiac toxicity ≥ CTCAE grade 3
	Clinical signs of cardiac disease, such as unstable angina or myocardial infarction, or Troponin ≥ grade 3
Gastro-intestinal	≥CTCAE grade 3 vomiting ≥48 hours despite optimal anti-emetic therapy
	≥CTCAE grade 3 diarrhea ≥48 hours despite optimal anti-diarrhea therapy
Hepato-biliary	≥CTCAE grade 2 total bilirubin for more than 7 consecutive days
	≥CTCAE grade 3 total bilirubin
	≥CTCAE grade 2 ALT or AST with total bilirubin > 2.0 x ULN without evidence of cholestasis*** OR<br>For subjects with abnormal baseline AST or ALT total bilirubin value:<br>[AST or ALT > 2x baseline AND > 3.0 x ULN] OR [AST or ALT > 8.0 x ULN], whichever is lower, combined with [total bilirubin > 2 x baseline AND > 2.0 x ULN]

	≥CTCAE grade 3 ALT for > 4 consecutive days
	CTCAE grade 4 ALT or AST
	CTCAE grade 4 serum alkaline phosphatase > 7 consecutive days
Renal	≥CTCAE grade 3 serum creatinine
Non-hematologic events	≥CTCAE grade 3, except for the exclusions noted below:
Exceptions to DLT criteria	<5 days of CTCAE grade 3 fatigue
	Grade 3 laboratory abnormalities that are responsive to oral supplementation or deemed by the investigator to be clinical insignificant
***"Cholestasis" defined as ALP elevation [>2.0 x ULN and R value < 2] in subjects without bone metastasis, or elevation of ALP liver fraction in subjects with bone metastasis<br>Note: The R value is calculated by dividing the ALT by the ALP, using multiples of the ULN for both values. It denotes whether the relative pattern of ALT and/or ALP elevation is due to cholestatic (R ≤ 2), hepatocellular (R ≥ 5), or mixed (R > 2 and < 5) liver injury.<br>CTCAE Version 4.03 should be used for grading<br>Optimal Therapy for vomiting and diarrhea should be based on institutional guidelines with consideration of the prohibited medications listed in these protocol guidelines.	

We will submit an efficacy/toxicity summary to IND Office Medical Monitor after the first six patients have completed the first cycle of study therapy.

3.1.3 EXPANSION PHASE

The safety lead-in portion of this trial has concluded and the RP2D has been identified for the experimental arm (everolimus, letrozole, and ribociclib) in the expansion phase. During the safety lead-in, two or more patients in both starting dose level 0 and dose level -1A experienced DLTs. Dose level -1 was well-tolerated, as previously stated in Section 3.1.1. Thus, patients that are randomized to the experimental arm will be given a starting dose of ribocliclib 200 mg, everolimus 2.5 mg, and letrozole 2.5 mg orally for 28-day cycles as outlined in Table 3A. Patients randomized to the control arm (everolimus and letrozole alone) will be given a starting dose of everolimus 10 mg and letrozole 2.5 mg orally for 28-day cycles.

3.2 SCHEMA

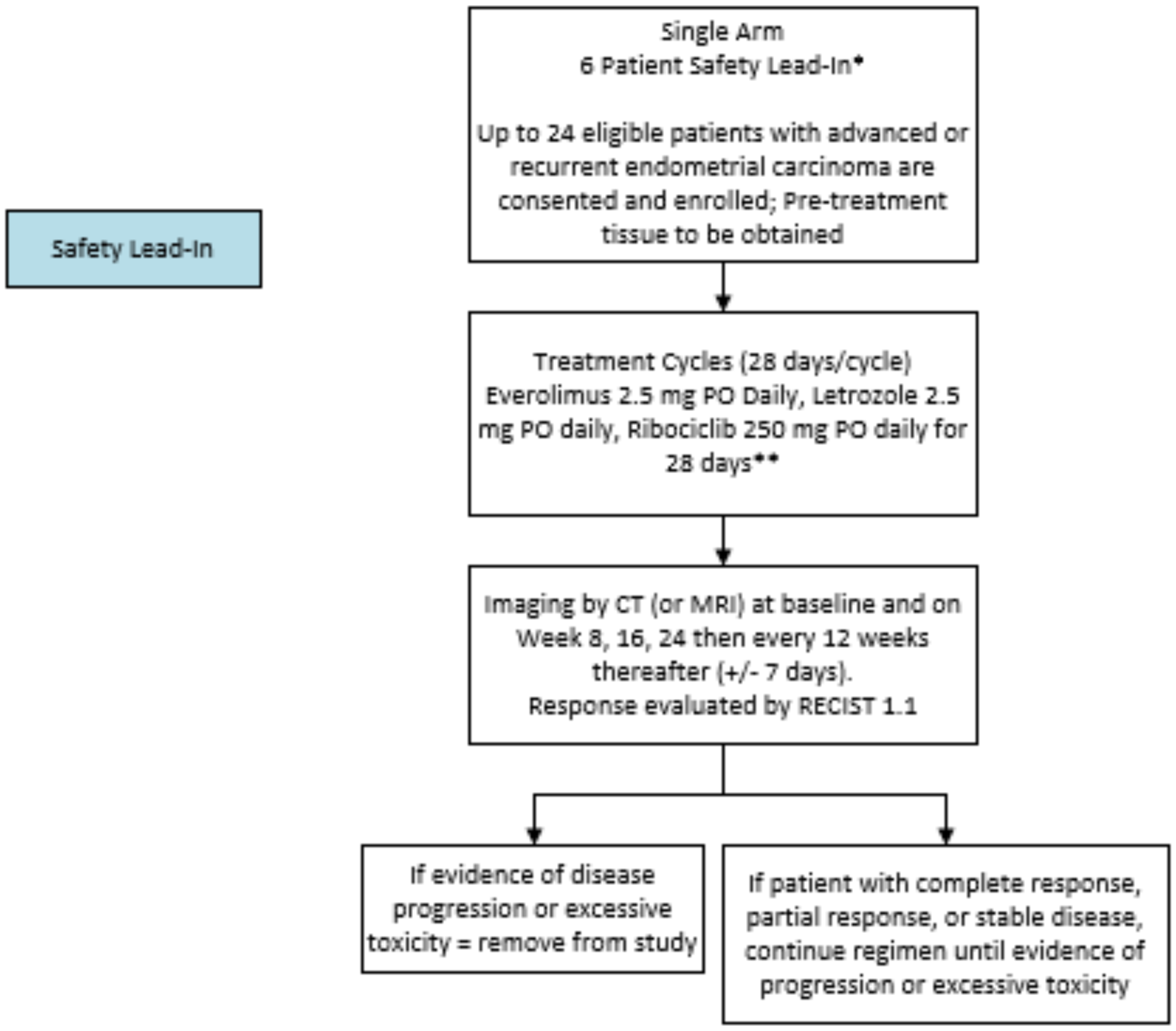

Figure 5. Safety Lead-In

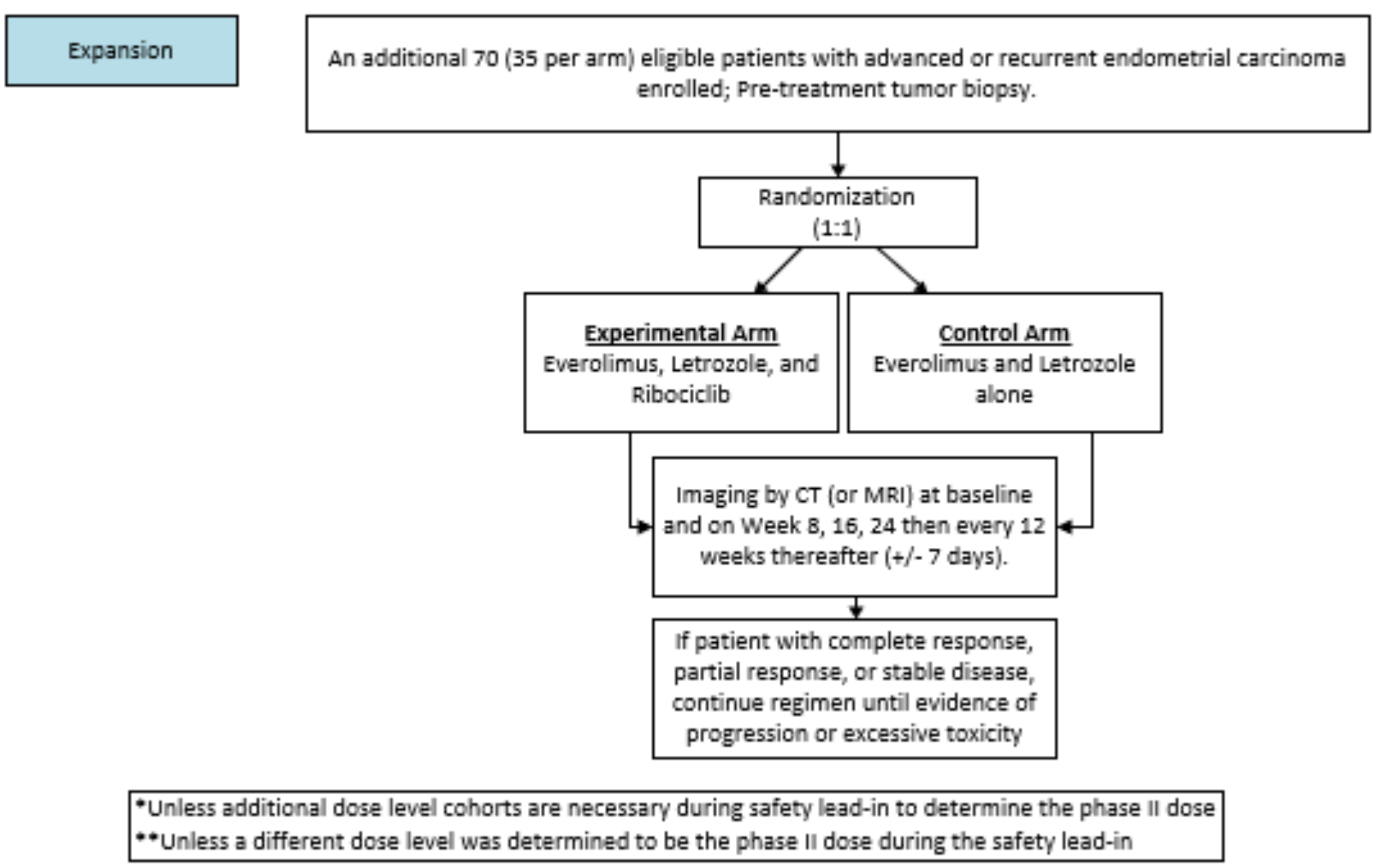

Figure 6. Expansion

3.3 PATIENT POPULATION

The target population is women who are ≥ 18 years of age with advanced or recurrent endometrial carcinoma who have not received more than 2 regimens of systemic chemotherapy for recurrent disease. This does not include chemo-sensitizing radiation. Females under the age of 18 are excluded as the safety and efficacy of letrozole has not been established in children.

We will enroll at least 14 patients with CTNNB1 mutation. The status of the CTNNB1 mutation will be determined by the Solid Genomic Tumor assay. This mutation status of patient accrual will be tracked and monitored in real time as results from the assay are received. If it is felt necessary by the Study PI, the protocol eligibility may be amended to require patients to have a CTNNB1 mutation in order to meet the 14 patient target for CTNNB1 mutations.

Patients must have baseline evaluations performed prior to the first dose of study drug and must meet all inclusion criteria. Results of all baseline evaluations must be reviewed by the Study PI or his/her designee prior to enrollment of that patient. In addition, the patient must be thoroughly informed about all aspects of the study, including the study visit schedule, required evaluations and all regulatory requirements for informed consent. The written informed consent must be obtained from the patient prior to enrollment.

The MDACC CORe system will be utilized for registration and will be a two-step process. Registration will be completed once the informed consent is signed for screening (step one). After eligibility is confirmed, registration will be completed (step two) and the patients will be randomized.

3.3.1 INCLUSION CRITERIA

1. Patient has signed the Informed Consent (ICF) prior to any screening procedures being performed and is able to comply with protocol requirements.
2. Patients must have histologically-confirmed endometrial carcinoma (endometrioid and mixed endometrioid tumors, any grade).
3. Patients must have advanced or recurrent disease that is refractory to curative treatment based on imaging or clinical exam.
4. Patient must consent to allow for a baseline tumor biopsy. Tumor material from biopsies done before the screening period are acceptable if the biopsy was performed within 3 months prior to the planned treatment start and no other systemic cancer therapy was administered in the interim. If a biopsy is performed and the specimen is considered non-diagnostic, does not have enough tissue, or the biopsy is deemed infeasible to perform, this does not prevent the patient from proceeding with the treatment.
5. Patients must have had no more than two prior chemotherapeutic regimens for recurrent endometrial carcinoma. Chemotherapy administered in conjunction with primary radiation as a radio-sensitizer is not counted as a prior treatment for recurrent or advanced disease.
6. Prior radiation therapy of any kind is allowed.
7. Prior treatment with letrozole is allowed if the patient meets the washout period of 10 days
8. All patients must have measurable disease per RECIST version 1.1 defined as at least one "target lesion" that can be accurately measured in at least one dimension (>/= 10 mm longest dimension to be recorded; Lymph nodes must be >/= 15 mm per short axis). Each lesion must be > 20 mm when measured by palpation or conventional imaging techniques (CT or MRI – based on primary physician preference) or >10 mm with spiral CT scan. Measurable lesions must be at least 2

times the slice thickness in millimeters. Tumors within a previously irradiated field will be designated as "non-target" lesions unless progression is documented. Ascites and pleural effusions are not considered measurable disease. If the measurable disease is confined to a solitary lesion, its neoplastic nature should be confirmed by cytology/histology.

9. Patients must not be pregnant, breastfeeding or of child-bearing potential. Patients are considered not of child-bearing potential if they are surgically sterile (they have undergone a total hysterectomy, bilateral tubal ligation, or bilateral oophorectomy) or they are postmenopausal for greater than 12 months (If patient is uncertain of amenorrhea for 12 months, a pregnancy test will be done to confirm pregnancy status). Patients in whom ovaries are present and were not previously menopausal at the time of hysterectomy, should have a serum estradiol <10 pm/mL to confirm ovarian senescence.
10. Patients must be off all other anti-tumor therapies (including immunologic agents) for at least four weeks prior to study registration. Patients on hormonal agents require a washout for 10 days
11. Age ≥ 18 years
12. GOG performance status of 0 or 1 (Appendix A)
13. Patient has adequate bone marrow and organ function as defined by the following laboratory values at screening:
    a. Absolute neutrophil count ≥1.5 × 10^9/L
    b. Platelets ≥100 × 10^9/L
    c. Hemoglobin ≥9.0 g/dL
    d. International normalized ratio (INR) < 1.5 x ULN and partial thromboplastin time (PTT) < 1.5 x ULN. PT can be used instead of INR if PTT < 1.5 x ULN (except when a patient is receiving anticoagulant therapy)
    e. Serum creatinine ≤1.5 mg/dL or creatinine clearance ≥50 mL/min
    f. Estimated glomerular filtration rate (eGFR) ≥ 30 mL/min/1.73m^2 according to the Chronic Kidney Disease Epidemiology Collaboration (CKD-EPI) equation formula
    g. In the absence of liver metastases, alanine aminotransferase (ALT) and aspartate aminotransferase (AST) <2.5 x ULN. If the patient has liver metastases, ALT and AST <5 x ULN
    h. Total bilirubin < ULN; or total bilirubin ≤3.0 x ULN or direct bilirubin ≤1.5 x ULN in patients with well-documented Gilbert's Syndrome.

    i. Fasting serum cholesterol ≤240 mg/dL OR ≤7.75 mmol/L AND fasting triglycerides ≤ 2.5 x ULN. NOTE: In case one or both of these thresholds are exceeded, the patient can only be included after initiation of appropriate lipid lowering medication.
14. Patient with available standard 12-lead ECG with the following parameters at screening:
    - QTc interval at screening <450msec (using Fridericia's correction)
    - Resting heart rate 50-100bpm

3.3.2 EXCLUSION CRITERIA

1. Patients who have uterine sarcomas, carcinosarcomas, serous tumors (any component) or pure clear cell carcinomas.

2. Patients currently receiving anticancer therapies or who have received anticancer therapies within 4 weeks of the start of study drug (including chemotherapy, radiation therapy, antibody based therapy, etc.)
3. Patient has not recovered from all toxicities related to prior anticancer therapies to NCI-CTCAE version 4.03 Grade ≤1 (Exception to this criterion: patients with grade 1 taxane-induced neuropathy, any grade of alopecia, amenorrhea or other toxicities not considered a safety risk for the patient as per investigator's discretion are allowed to enter the study).
4. Patient has had major surgery within 14 days prior to starting study drug or has not recovered from major side effects (tumor biopsy is not considered as major surgery).
5. Participation in other studies involving investigational drug(s) within 30 days prior to randomization or within 5 half-lives of the investigational product (whichever is longer) or participation in any other type of medical research judged not to be scientifically or medically compatible with this study.
6. Patients receiving chronic, systemic treatment with corticosteroids or another immunosuppressive agent. Topical, short duration (<5 days) of systemic corticosteroids, eye drops, local injections, or inhaled corticosteroids, or ≤10 mg daily prednisone or equivalent are allowed.
7. Patients should not receive immunization with attenuated live vaccines within one week of study entry or during study period. Close contact with those who have received attenuated live vaccines should be avoided during treatment with everolimus. Examples of live vaccines include intranasal influenza, measles, mumps, rubella, oral polio, BCG, yellow fever, varicella and TY21a typhoid vaccines.
8. Patients with central nervous system (CNS) involvement unless they meet ALL of the following criteria:
   - At least 4 weeks from prior therapy completion (including radiation and/or surgery) to starting the study treatment
   - Clinically stable CNS tumor at the time of screening and not receiving steroids and/or enzyme-inducing anti-epileptic medications for brain metastases.
9. Patient has a concurrent malignancy or malignancy within 3 years prior to starting study drug, with the exception of adequately treated, basal or squamous cell carcinoma or curatively resected cervical cancer in situ.
10. Patient has any other concurrent severe and/or uncontrolled medical condition that would, in the investigator's judgment, may cause unacceptable safety risks, contraindicate patient participation in the clinical study or compromise compliance with the protocol (e.g. chronic pancreatitis, chronic active hepatitis, active untreated or uncontrolled fungal, bacterial or viral infections, etc.)
11. Patient has a known history of HIV infection (testing not mandatory).
12. Clinically significant, uncontrolled heart disease and/or cardiac repolarization abnormalities, including any of the following:
    - History of acute coronary syndromes (including myocardial infarction, unstable angina, coronary artery bypass grafting, coronary angioplasty, or stenting) or symptomatic pericarditis within 6 months prior to screening
    - History of documented congestive heart failure (New York Heart Association functional classification III-IV)
    - Documented cardiomyopathy
    - Left Ventricular Ejection Fraction (LVEF) <50% as determined by Multiple Gated acquisition (MUGA) scan or echocardiogram (ECHO) at screening

- Clinically significant cardiac arrhythmias (e.g. ventricular tachycardia), complete left bundle branch block, high-grade AV block (e.g. bifascicular block, Mobitz type II and third-degree AV block)
- Long QT syndrome or family history of idiopathic sudden death or congenital long QT syndrome, or any of the following:
    - Risk factors for Torsades de Pointe (TdP) including uncorrected hypocalcemia, hypokalemia or hypomagnesemia, history of cardiac failure, or history of clinically significant/symptomatic bradycardia.
    - Concomitant use of medication(s) with a known risk to prolong the QT interval and/or known to cause Torsades de Pointe that cannot be discontinued (within 5 half-lives or 7 days prior to starting study drug) or replaced by safe alternative medication
    - Inability to determine the QT interval on screening (QTcF, using Fridericia's correction)
    - Systolic blood pressure (SBP) >160 mmHg or <90 mmHg at screening

13. Impairment of gastrointestinal function or gastrointestinal disease that may significantly alter the absorption of study medication (e.g., ulcerative disease, uncontrolled nausea, vomiting, diarrhea, malabsorption syndrome or small bowel resection associated with malabsorption)
14. Patient is currently receiving any of the following medications and cannot be discontinued 7 days prior to starting study drug (see Appendix B for details):
    - Known strong inducers or inhibitors of CYP3A4/5, including grapefruit, grapefruit hybrids, pummelos, star-fruit, and Seville oranges
    - That have a narrow therapeutic window and are predominantly metabolized through CYP3A4/5
    - Herbal preparations/medications, dietary supplements.
    - Hormone replacement therapy, topical estrogens (including any intra-vaginal preparations), megestrol acetate and selective estrogen-receptor modulators (e.g. raloxifene).
15. Patient is currently receiving warfarin or other coumarin-derived anticoagulant for treatment, prophylaxis or otherwise. Therapy with heparin, low molecular weight heparin (LMWH) or fondaparinux is allowed.
16. Liver disease such as cirrhosis or severe hepatic impairment (Patient with a Child-Pugh score B or C).
    Note: A detailed assessment of Hepatitis B/C medical history and risk factors (see 3.5.1) must be done at screening for all patients. HBV DNA and HCV RNA PCR testing are required at screening for all patients with a positive medical history based on risk factors and/or confirmation of prior HBV/HCV infection.
17. Patients who have received prior treatment with an mTOR inhibitor (e.g., sirolimus, temsirolimus, everolimus).
18. Patients with a known hypersensitivity to ribociclib or everolimus or to its excipients
19. History of noncompliance to medical regimens.
20. Patients unwilling to or unable to comply with the protocol.

3.3.3 INCLUSION OF WOMEN AND MINORITIES

Women of all racial and ethnic groups are eligible for this trial.

3.4 TREATMENTS

3.4.1 RIBOCICLIB, EVEROLIMUS, AND LETROZOLE ADMINISTRATION

This is an open-label, randomized 2-arm study. Patients will receive either everolimus and letrozole versus ribociclib, everolimus, and letrozole. The study drugs ribociclib, everolimus, and letrozole will be self-administered by the patients. The investigator will instruct the patient to take the study drug exactly as specified in the protocol. All medications should be taken at the same time each day.

- Patients should be instructed to take the study drug with a large glass of water (about 8 oz) daily in the morning at approximately the same time every day. Patients should be instructed to swallow the tablets whole and not to chew, crush or open them.
- On all dose administration days patients should not eat anything within 2 hours prior to study drug intake and refrain from eating or drinking additional liquids for 2 hours following Ribociclib intake.
- All drugs will be dispensed by the pharmacy. Study medication will be supplied in an open label fashion.
- If vomiting occurs, no attempt should be made to replace the vomited dose.
- Any doses that are missed (not taken within 6 hours of the intended time) should be skipped and should not be replaced or made up on a subsequent day.
- All dosages prescribed and dispensed to the patient and all dose changes during the study must be recorded. If a patient misses 10% or less of the dose prescribed in each cycle it will not be considered a deviation.
- Patients must avoid consumption of grapefruit, grapefruit hybrids, pummelos, star-fruit, Seville oranges or products containing the juice of each during the entire study and preferably 7 days before the first dose of study medication, due to potential CYP3A4 interaction with the study medications. Orange juice is allowed.
- No herbal or dietary supplements are permitted, due to potential interactions with ribociclib; multivitamins are allowed.
- Each cycle consists of 28 days (+/- 10 days) of therapy. Patients will have labs drawn per treatment schedule (Tables 5 and 6) and be evaluated in clinic prior to each cycle. To allow for holidays, vacations, and other important life events, patients may be seen +/- 10 days of their return visit date. Any other schedule modifications must be approved by the study chair. In these circumstances, patients may be dispensed additional medication in order not to interrupt treatment.
- On a day of chemistry panel and/or lipid panel sampling, patients must be fasting from all food and drink for at least 8 hours overnight. Water is allowed during all fasting periods; however coffee, tea and juice are not permitted during the fasting period.

Medication labels will comply with US legal requirements and be printed in English. They will supply no information about the patient. The storage conditions for study drug will be described on the medication label.

3.4.2 TREATMENT SCHEDULES

Table 5. Evaluation and visit schedule for Safety Lead-in

Assessments	Baseline	Cycle 1 Day 1(cc)	Cycle 1 Day 15(cc)	Cycle 2 Day 1(cc)	Cycle 2 Day 15(cc)	Cycle 3+ Day 1(cc)	End of Treatment	Follow-up
Informed Consent	X							
Medical History	X(a)							
Concomitant Meds	X(b)	X		X		X	X	
Physical Examination(c)	X(b,c)	X(c)		X(c)		X(c)	X	
Performance Status	X(b)	X		X		X	X	
Vital Signs(d)	X(b)	X		X		X	X	
Height	X(b)							
O^2 saturation on room air	X(b)							
CBC w/diff, platelets	X(b)	X	X	X		X	X	
Chemistry Panel(e)	X(f)(b)	X	X	X		X	X	
Coagulation Panel(g)	X(b)			X		X	X	
Lipid Panel(h)(i)	X(b)					X(i)		
Pregnancy Test	X(J)							
Serum Estradiol(k)	X(k)							
HIV blood test	X(a)(l)							
Hepatitis Screening	X(m)(0)							
Hepatitis Monitoring				X(n)p)(q)		X(n)(p)(q)		
Electrocardiogram (ECG/EKG)	X(b)(r)		X(s)	X(r)	X(t)	X(r)		
Echocardiogram (ECHO)	X(a)							
Adverse Event Evaluation		X(u)--X(u)						X(u)
Medication Reconciliation (pill count) Dispensation/ Accountability(v)		X		X		X	X	
Pharmacodynamic Blood(w)	X		X					
Baseline biopsy or tissue(x)	X							
Solid Tumor Genomic Assay(y)	X							
Tumor Assessments (z)	X(a)			X(aa,z)		X(aa,z)	X(aa,z)	
Survival Status								X(bb)

(a) Within 4 weeks prior to Step 2 of study registration as defined in **Section 3.3.1**
(b) Within 7 days of initiation of therapy
(c) Pelvic exam is performed at baseline. If patient has palpable disease at baseline, then to be performed every 2 cycles for the first 6 cycles and then every 3rd cycle thereafter.
(d) Vitals signs include: pulse, blood pressure, respiration rate, temperature and weight. Blood pressure, pulse and respiration rate should be measured on patients after at least 3 minutes in the sitting position.
(e) Chemistry Panel includes: sodium, potassium, chloride, bicarbonate, magnesium, glucose, creatinine, blood urea nitrogen (BUN), SGPT (ALT), SGOT (AST), total bilirubin, total protein, albumin, calcium, phosphorous, alkaline phosphatase.
(f) Creatinine must be less than 1.5 mg/dL or Creatinine clearance of greater than 50 mL/min at baseline
(g) Coagulation Panel includes: PT, PTT and INR
(h) Lipid Panel includes: Cholesterol, Triglycerides, HDL, LDL
(i) To be obtained at baseline, and day 1 of every third cycle, thereafter.
(j) Women who are menopausal but cannot confirm 12 month of natural (spontaneous) amenorrhea will need to have a negative pregnancy test within 7 days of initiation of therapy.
(k) Documentation of a serum estradiol < 10 pg/mL is required only for patients in whom hysterectomy was performed pre-menopausal and in whom bilateral oophorectomy was not completed. Estradiol is not required for women known to be menopausal or surgically castrated.
(l) HIV testing is not mandatory at baseline per eligibility criteria. Patient may opt to not undergo testing.
(m) Patients who qualify for high-risk categories as outlined in **Section 6.1** should be tested for Hepatitis B serologic markers and viral load as follows: HBV-DNA, HBsAg, HbcAB, and HBsAb.
(n) HBV-DNA monitoring should be performed depending on results from serologic markers and viral load as listed in **Section 5.2.8**
(o) Patients who qualify for high-risk categories for Hepatitis C as outlined in **Section 6.1** or at risk per physician's discretion should be tested for Hepatitis C Virus as follows: HCV RNA-PCR
(p) Follow-up testing Hepatitis C will be performed, as per the visit schedule, only if patient has a history or is positive at baseline, or both.
(q) Patients with detectable HCV RNA-PCR (determined at baseline) and patients known to have history of HCV infection, despite a negative viral load test at baseline (including those that were treated and considered 'cured') should be monitored every 4 weeks for HCV reactivation.
(r) Single electrocardiogram (ECG/EKG, 12-lead) to be performed at baseline and pre-dose at C2D1, C3D1, C4D1, C5D1, and C6D1.
(s) Triplicate electrocardiogram (ECG/EKG, 12-lead) to be performed 5 minutes apart, pre-dose and 2-4 hours post-dose on C1D15.
(t) Single electrocardiogram (ECG/EKG, 12-lead) to be performed pre-dose and 2-4 hours post-dose on C2D15 only.
(u) Adverse Events will be assessed on an ongoing basis from the first dose of study treatment until 30 days after the last dose of study drug.
(v) Patients should bring their unused medication to the clinic at each visit. Compliance should be verified by a patient pill diary and by counting the number of tablets consumed between visits using a pill count clarification document. Study medication will be dispensed.
(w) Please see **Section 3.5.1** for details and schedule collection of pharmacodynamics blood. Baseline pharmacodynamic test may be obtained any time prior to cycle 1 day 1.

(x) Tumor tissue from tumor biopsies must be submitted prior to treatment for retrospective biomarker analyses. Tumor biopsies may be done during the screening period prior to study treatment start. Tumor material from biopsies done before the screening period is acceptable if the biopsy was performed within 3 months prior to the planned treatment start and no other systemic cancer therapy was administered in the meanwhile. If a biopsy is performed and the specimen is considered non-diagnostic or does not have enough tissue, this does not prevent the patient from proceeding with the treatment. Please see **Section 3.5.4** for details regarding collection.

(y) Tumor material from the baseline biopsy or archival tissue to be submitted for Solid Tumor Genomic Assay (MDA only)

(z) Tumor imaging to include CT/MRI of chest, abdomen and pelvis and/or appropriate imaging. RECIST tumor assessments and tumor imaging will be performed at baseline and on week 8, 16, 24 then every 12 weeks thereafter (+/- 7 days).

(aa) CT/MRI of the chest to be performed only in patients with chest disease at baseline.

(bb) Patients will be contacted by phone or assessed at a regularly scheduled clinic visit every 3 months (+/- 1 month) to assess their vital status (dead or alive).

(cc) Laboratory assessments to be completed within 7 days of scheduled clinic visit each cycle. If baseline laboratory assessments are done within 7 days of C1D1 they do not need to be repeated for C1D1. Patients should be fasting prior to all lab studies.

Table 6A. Evaluation and visit schedule for Phase II – Experimental Arm

Assessments	Baseline	Cycle 1 Day 1[bb]	Cycle 1 Day 15[bb]	Cycle 2 Day 1[bb]	Cycle 3+ Day 1[bb]	End of Treatment	Follow-up
Informed Consent	X						
Medical History	X[a]						
Concomitant Meds	X[b]	X		X	X	X	
Physical Examination[c]	X[b,c]	X[c]		X[c]	X[c]	X	
Performance Status	X[b]	X		X	X	X	
Vital Signs[d]	X[b]	X		X	X	X	
Height	X[b]						
O^2 saturation on room air	X[b]						
CBC w/diff, platelets	X[b]	X	X	X	X	X	
Chemistry Panel[e]	X[f][b]	X	X	X	X	X	
Coagulation Panel[g]	X[b]			X	X	X	
Lipid Panel[h][i]	X[b]				X[i]		
Pregnancy Test	X[J]						
Serum Estradiol[k]	X[k]						
HIV blood test	X[a][l]						
Hepatitis Screening	X[m][0]						
Hepatitis Monitoring				X[n]p)[q]	X[n][p][q]		
Electrocardiogram (ECG/EKG)	X[b][r]		X[s]	X[r]	X[r]		
Echocardiogram (ECHO)	X[a]						
Adverse Event Evaluation		X[t]--X[t]					X[t]
Medication Reconciliation (pill count) Dispensation/ Accountability[u]		X		X	X	X	
Pharmacodynamic Blood[v]	X		X				
Liquid Biopsy[v]	X				X		
Baseline biopsy or tissue[w]	X						
Solid Tumor Genomic Assay[x]	X						
Tumor Assessments [y]	X[a]			X[y,z]	X[y,z]	X[y,z]	
Survival Status							X[aa]

Table 6B. Evaluation and visit schedule for Phase II – Control Arm

Assessments	Baseline	Cycle 1 Day 1(bb)	Cycle 2 Day 1(bb)	Cycle 3+ Day 1(bb)	End of Treatment	Follow-up
Informed Consent	X					
Medical History	X(a)					
Concomitant Meds	X(b)	X	X	X	X	
Physical Examination(c)	X(b,c)	X(c)	X(c)	X(c)	X	
Performance Status	X(b)	X	X	X	X	
Vital Signs(d)	X(b)	X	X	X	X	
Height	X(b)					
O^2 saturation on room air	X(b)					
CBC w/diff, platelets	X(b)	X	X	X	X	
Chemistry Panel(e)	X(f)(b)	X	X	X	X	
Coagulation Panel(g)	X(b)		X	X	X	
Lipid Panel(h)(i)	X(b)			X(i)		
Pregnancy Test	X(J)					
Serum Estradiol(k)	X(k)					
HIV blood test	X(a)(l)					
Hepatitis Screening	X(m)(0)					
Hepatitis Monitoring			X(n)p)(q)	X(n)(p)(q)		
Electrocardiogram (ECG/EKG)	X(b)					
Echocardiogram (ECHO)	X(a)					
Adverse Event Evaluation		X(t) ---			-X(t)	X(t)
Medication Reconciliation (pill count) Dispensation/ Accountability(u)		X	X	X	X	
Liquid Biopsy(v)	X			X		
Baseline biopsy or tissue(w)	X					
Solid Tumor Genomic Assay(x)	X					
Tumor Assessments	X(a)		X(y,z)	X(y,z)	X(y,z)	
Survival Status						X(aa)

(a) Within 4 weeks prior to Step 2 of study registration as defined in **Section 3.3.1**
(b) Within 7 days of initiation of therapy
(c) Pelvic exam is performed at baseline. If patient has palpable disease at baseline, then to be performed every 2 cycles for the first 6 cycles and then every 3rd cycle thereafter.
(d) Vitals signs include: pulse, blood pressure, respiration rate, temperature and weight. Blood pressure, pulse and respiration rate should be measured on patients after at least 3 minutes in the sitting position.
(e) Chemistry Panel includes: sodium, potassium, chloride, bicarbonate, magnesium, glucose, creatinine, blood urea nitrogen (BUN), SGPT (ALT), SGOT (AST), total bilirubin, total protein, albumin, calcium, phosphorous, alkaline phosphatase.
(f) Creatinine must be less than 1.5 mg/dL or Creatinine clearance of greater than 50 mL/min at baseline
(g) Coagulation Panel includes: PT, PTT and INR
(h) Lipid Panel includes: Cholesterol, Triglycerides, HDL, LDL
(i) To be obtained at baseline, and day 1 of every third cycle, thereafter.
(j) Women who are menopausal but cannot confirm 12 month of natural (spontaneous) amenorrhea will need to have a negative pregnancy test within 7 days of initiation of therapy.
(k) Documentation of a serum estradiol < 10 pg/mL is required only for patients in whom hysterectomy was performed pre-menopausal and in whom bilateral oophorectomy was not completed. Estradiol is not required for women known to be menopausal or surgically castrated.
(l) HIV testing is not mandatory at baseline per eligibility criteria. Patient may opt to not undergo testing.
(m) Patients who qualify for high-risk categories as outlined in **Section 6.1** should be tested for Hepatitis B serologic markers and viral load as follows: HBV-DNA, HBsAg, HbcAB, and HBsAb.
(n) HBV-DNA monitoring should be performed depending on results from serologic markers and viral load as listed in **Section 5.2.8**
(o) Patients who qualify for high-risk categories for Hepatitis C as outlined in **Section 6.1** or at risk per physician's discretion should be tested for Hepatitis C Virus as follows: HCV RNA-PCR
(p) Follow-up testing Hepatitis C will be performed, as per the visit schedule, only if patient has a history or is positive at baseline, or both.
(q) Patients with detectable HCV RNA-PCR (determined at baseline) and patients known to have history of HCV infection, despite a negative viral load test at baseline (including those that were treated and considered 'cured') should be monitored every 4 weeks for HCV reactivation.
(r) Single electrocardiogram (ECG/EKG, 12-lead) to be performed at baseline and pre-dose at C2D1, C3D1, C4D1, C5D1, and C6D1.
(s) Single electrocardiogram (ECG/EKG, 12-lead) to be performed pre-dose and 2-4 hours post-dose on C1D15.
(t) Adverse Events will be assessed on an ongoing basis from the first dose of study treatment until 30 days after the last dose of study drug.
(u) Patients should bring their unused medication to the clinic at each visit; during remote visits, the patient will be asked to dispose of their medication at a local DEA take-back location or pharmacy offering medication disposal services. Compliance should be verified by a patient pill diary and by counting the number of tablets consumed between visits using a pill count clarification document. Study medication will be dispensed.
(v) Please see **Section 3.5.1** for details and schedule collection of pharmacodynamics blood or section 3.5.7 for Liquid biopsy details. Baseline collections may be obtained any time prior to cycle 1 day 1.

(w) Tumor tissue from tumor biopsies must be submitted prior to treatment for retrospective biomarker analyses. Tumor biopsies may be done during the screening period prior to study treatment start. Tumor material from biopsies done before the screening period is acceptable if the biopsy was performed within 3 months prior to the planned treatment start and no other systemic cancer therapy was administered in the meanwhile. If a biopsy is performed and the specimen is considered non-diagnostic or does not have enough tissue, this does not prevent the patient from proceeding with the treatment. Please see **Section 3.5.2** for details regarding collection.

(x) Tumor material from the baseline biopsy or archival tissue to be submitted for Solid Tumor Genomic Assay (MDA only)

(y) Tumor imaging to include CT/MRI of chest, abdomen and pelvis and/or appropriate imaging. RECIST tumor assessments and tumor imaging will be performed at baseline and on week 8, 16, 24 then every 12 weeks thereafter (+/- 7 days).

(z) CT/MRI of the chest to be performed only in patients with chest disease at baseline.

(aa) Patients will be contacted by phone or assessed at a regularly scheduled clinic visit every 3 months (+/- 1 month) to assess their vital status (dead or alive).

(bb) Laboratory assessments to be completed within 7 days of scheduled clinic visit each cycle. If baseline laboratory assessments are done within 7 days of C1D1 they do not need to be repeated for C1D1. Patients should be fasting prior to all lab studies.

3.4.3 RANDOMIZATION AND STRATIFICATION

Eligible patients will be randomized (1:1 ratio) to receive ribociclib, everolimus, and letrozole (experimental arm) or everolimus and letrozole (control arm). To ensure the equal distribution of prognostic factor in the two study arms, patients will be stratified according to the following parameters
• previous radiation.
All patients must commence treatment within 7 days following randomization; otherwise, the screening assessments will need to be repeated.

3.5 CORRELATIVE STUDIES

3.5.1 PHARMACODYNAMIC BLOOD COLLECTION SCHEDULE

Prior to Cycle 1 Day 1 and on Cycle 1 Day 15, 20 ml blood will be collected for pharmacodynamic testing in patients on the ribociclib, everolimus, and letrozole arm. Please refer to the Lab Manual for specific collection and processing details.

3.5.2 BASELINE TUMOR MATERIAL

Tumor tissue from tumor biopsies must be submitted prior to treatment for retrospective biomarker analyses. Tumor biopsies may be done during the screening period prior to study treatment start. Tumor material from biopsies done before the screening period are acceptable if the biopsy was performed within 3 months prior to the planned treatment start and no other systemic cancer therapy was administered in the meanwhile. If a biopsy is performed and the specimen is considered non-diagnostic, does not have enough tissue, or the biopsy is deemed infeasible to perform, this does not prevent the patient from proceeding with the treatment.

3.5.3 COLLECTION OF TUMOR SPECIMENS

If the patient undergoes a pre-treatment biopsy, part of this sample will be sent to pathology for routine diagnostic testing and up to 4 additional cores (18 gauge or larger) will be obtained for research.

If available, archived samples may also be requested from the patient's primary surgery for immunohistochemical expression testing.

3.5.4 HANDLING, LABELING AND STORAGE OF RESEARCH SPECIMENS

Pre-treatment research samples (archived tissue or fresh) will be collected by the research staff and stored securely by the study PI and/or their designee in a locked cabinet. The research tissue specimens will be formalin-fixed and paraffin embedded into blocks.

If the tissue specimen was not obtained at MD Anderson, routine handling of unstained slides and tumor blocks will be employed. Shipping of specimens will be via courier services in secure containers when specimens are not available at the study center.

Each specimen will be labeled with a study accession number, a specimen name, and collection date. As applicable, the specimen codes will be used as follows:

The specimen name 'Pre-Tx' will be assigned for the formalin-fixed and paraffin embedded tissue obtained from a pre-treatment tissue biopsy.

The specimen name 'Primary' will be assigned for the archived tissue obtained from the patient's primary surgery or biopsy.

3.5.5 SITE PERFORMING THE CORRELATIVE STUDY

All assays will be performed at The University of Texas MD Anderson Cancer Center.

3.5.6 THE SOLID TUMOR GENOMIC ASSAY

The Solid Tumor Genomic Assay (STGA) molecular testing will be performed on MD Anderson pre-treatment samples as standard of care. The STGA molecular testing will not be performed on tumor tissue samples from The Harris Health System (LBJ) as the test is not offered at LBJ. The MD Anderson Molecular Diagnostics Laboratory will be performing the Solid Tumor Genomic Assay Panel testing which will include the *CTNNB1* gene sequencing and mutation analysis.

This panel has gone through several name changes and may also change in the future. Study staff will use the appropriate panel that is currently available at the time.

3.5.7 BIOMARKER STUDIES

We will use immunohistochemistry to evaluate the expression of PTEN, mTOR, AKT, p70S6K, and ERK1/2 using commercially available antibodies. Additional relevant biomarkers may be tested using immunohistochemistry or other molecular techniques. Blood for liquid biopsies will be drawn at baseline, prior to initiation of therapy and again at 8 weeks (completion of cycle 2) for the first 15 patients in each treatment group.

3.5.8 FUTURE RESEARCH

The patient will have the option to allow their leftover PD blood and tissue samples from the study to be stored in the Gynecologic Oncology Tumor Bank and used for future research.

Before leftover tissue samples can be used for research, the people doing the research must get specific approval from the Institutional Review Board (IRB) of MD Anderson.

4 CONCOMITANT THERAPY

Patients will be instructed not to take any additional medications (including over-the-counter products) during the course of the study without prior consultation with the investigator. At each visit, the investigator will ask the patient about any new medications he/she is or has taken after the start of the study drug.

All concomitant medications/significant non-drug therapies taken ≤ 30 days prior to start and after start of study drug and blood transfusions will be maintained in the medical record.

The following restrictions apply during the entire duration of the study:

a. No other investigational therapy should be given to patients.
b. No anticancer agents other than the study medication should be given to patients. If such agents are required for a patient, then the patient must first be withdrawn from the study.
c. It is highly recommended that patients positive HBV-DNA or HBsAg are treated prophylactically with an antiviral for 1-2 weeks prior to receiving study drug (see Section 3.2).
d. The antiviral treatment should continue throughout the entire study period and for at least 4 weeks after the last dose of study drug.
e. Patients on antiviral prophylaxis treatment or positive HBV antibodies should be tested for HBV-DNA according to study visit schedule.

4.1 INHIBITORS OF CYP3A4 AND/OR PGP

- Co-administration with strong inhibitors of CYP3A4 (e.g., ketoconazole, itraconazole, ritonavir) or P-glycoprotein (PgP) should be avoided **(see** Appendix B).
- Co-administration with moderate CYP3A4 inhibitors (e.g., erythromycin, fluconazole) or PgP inhibitors should be used with caution. **If patient requires co-administration of moderate CYP3A4 inhibitors or PgP inhibitors, the dose of everolimus should be no greater than 2.5 mg daily**. Additional dose reductions to every other day may be required to manage toxicities. If the inhibitor is discontinued the everolimus dose should be returned to the dose used prior to initiation of the moderate CYP3A4/PgP inhibitor after a washout period of 2 to 3 days. See appendix B for list of medications that are prohibited while on trial and should be avoided. Cautionary medications are at the discretion of the treating physician.
- Seville orange, star fruit, pummelos, grapefruit, grapefruit hybrids, and their juices affect P450 and PgP activity. Concomitant use should be avoided.
- See **Appendix B** for list of inhibitors of CYP3A4 and/or PgP.

4.2 INDUCERS OF CYP3A4 AND/OR PGP

- Avoid the use of strong CYP3A4 inducers for at least 7 days prior to starting on trial (see Appendix B). **If patient requires co-administration of strong CYP3A4 inducers** (i.e., phenytoin, carbamazepine, rifampin, rifabutin, phenobarbital, St. John's wort)**, an increase in the dose of everolimus from 2.5 mg daily to 5 mg daily, or from 5 mg to 10 mg depending on the dose level**. When the strong CYP3A4 inducer is discontinued, everolimus dose will be reduced to prior dose level.
- This dose adjustment of everolimus is intended to achieve similar AUC to the range observed without inducers. However, there are no clinical data with this dose adjustment in patients receiving strong CYP3A4 inducers. If the strong inducer is discontinued the everolimus dose should be returned to the dose used prior to initiation of the strong CYP3A4/PgP inducer.
- See Appendix B for list of inducers of CYP3A4 and/or PgP.

5 DISCONTINUATION, INTERRUPTIONS AND DOSE ADJUSTMENTS

All interruptions or changes to study drug administration must be recorded

5.1 DISCONTINUATION OF TREATMENT

It will be documented whether or not each patient completed the clinical study. If for any patient either study treatment or observations were discontinued the reason will be recorded.
Reasons that a patient may discontinue participation in a clinical study are considered to constitute one of the following:

1. adverse event(s)
2. abnormal laboratory value(s)
3. abnormal test procedure result(s)
4. determination by the physician it is in the best interest of the patient to stop treatment.
5. disease progression
6. protocol violation
7. patient withdrew consent

8. non-compliance by the patient with protocol requirements.
9. lost to follow-up
10. termination of the study by the Study PI or Sponsor
11. death

5.2 DOSE ADJUSTMENTS:

For patients who are unable to tolerate the protocol-specified dosing schedule, dose adjustments are permitted in order to keep the patient on study drug (Table 7A and Table 7B). If administration of Ribociclib must be interrupted because of unacceptable toxicity, drug dosing will be interrupted or modified according to rules described in Table 8. Patients who must discontinue Ribociclib indefinitely due to toxicity may remain on study with everolimus and letrozole; everolimus will be increased to 10 mg daily. Toxicity will be assessed using the NIH-NCI Common Terminology Criteria for Adverse Events v4.03 (CTCAEv4.03).

Table 7A. Dose reduction table for patients in the Safety Lead-In and Experimental Arm

Dose Level	Everolimus Dose	Letrozole Dose	Ribociclib Dose
-3	5 mg daily	2.5 mg daily	200mg daily 21 days on, 7 days off
-2	2.5 mg daily	2.5 mg daily	300 mg daily 21 days on, 7 days off
-1*	2.5 mg daily	2.5 mg daily	200 mg daily for 28 days
-1A	5 mg	2.5 mg daily	200 mg daily for 28 days
0	2.5 mg daily	2.5 mg daily	250 mg daily for 28 days

*Please note the Expansion Phase (RP2D) starting dose (Table 3A).

Table 7B. Dose reduction table for patients in the Control Arm

Dose Level	Everolimus Dose	Letrozole Dose	Ribociclib Dose
-3	Off Study	Off Study	N/A
-2	2.5 mg daily	2.5 mg daily	N/A
-1	5 mg daily	2.5 mg daily	N/A
0	10 mg daily	2.5 mg daily	N/A

Everolimus dose level -3 is increased to 5 mg daily in order to maintain a therapeutic dose of Everolimus. Due to the synergistic effect of dosing ribociclib and everolimus, it is necessary to increase the everolimus dose when reducing ribociclib to 200 mg daily 21 days on 7 days off, if the everolimus is maintained at 2.5 mg, patients will be below the therapeutic dose for everolimus.

Prior to starting each cycle, non-hematologic toxicities should have resolved to Grade 1 or may be grade 2 if tolerable to the patient (electrolyte abnormalities should resolve to at least Grade 2) and hematologic toxicities to Grade 2 (Table 8).

Patients will be removed from study for any:

- Grade 4 treatment-related non-hematologic toxicity (assuming that appropriate preventive therapy has been give where possible), or
- Hematologic or non-hematologic toxicity requiring interruption of drug therapy for more than 4 weeks, or
- Recurrent/persistent hematologic or non-hematologic toxicities resulting in greater than 2 dose reductions

Table 8. Criteria for dose-modification in case of suspected "treatment related" toxicity

Toxicity	Actions
Non-hematological toxicity	
Grade 2<br>(except hyperlipidemia* - refer to Section 5.2.6)<br>(except pneumonitis – refer to Section 5.2.7)<br>(except AST/ALT and/or total bilirubin – refer to Section 5.2.1)<br>(except QT prolongation – refer to Section 5.2.2)<br>(except Renal Impairment – refer to Section 5.2.10)	If the toxicity is tolerable to the patient, maintain the same dose. If the toxicity is intolerable to patient, interrupt the likely causative agent until recovery to grade ≤1 within 28 days. Then reintroduce that agent at same dose.<br>If event recurs within 28 days, hold the likely causative agent until recovery to ≤ grade 1 or baseline grade / value. If the event resolves ≤ 7 days all agents should be re-started at same dose. If resolution takes > 7 days reintroduce the medication at one dose level lower, if available.<br>For grade 2 hyperglycemia, no dose adjustment is required, manage with appropriate medical therapy.
Grade 3<br>(except hyperlipidemia – refer to Section 5.2.6)<br>(except pneumonitis – refer to Section 5.2.7)<br>(except AST/ALT and/or total bilirubin – refer to Section 5.2.1)<br>(except QT prolongation – refer to Section 5.2.2)<br>(except Renal Impairment – refer to Section 5.2.10)	For the first occurrence, interrupt the likely causative agent until recovery to grade ≤1. If resolution occurs ≤ 7 days, all agents should be re-started at the dose level prior to interruption.<br>If resolution takes > 7 days, or if event recurs within 28 days, hold the likely causative agent until recovery to ≤ grade 1 or baseline grade / value and reintroduce the medications at one dose level lower, if available.<br>Patients will be withdrawn from the study if they fail to recover to ≤ grade 1 or baseline grade / value within 28 days.<br>For grade 3 hyperlipidemia, no dose adjustment is required, manage with appropriate medical therapy.
Grade 4	Hold everolimus, ribociclib, and letrozole until recovery to ≤ grade 1 or baseline grade / value and reintroduce the medications at one dose level lower, if available. Patients will be withdrawn from the study if they fail to recover to ≤ grade 1 or baseline grade / value within 28 days.
Hematological toxicity	
Grade 2 Thrombocytopenia (platelets <75, ≥ $50x10^9/L$)	Hold ribociclib until recovery to ≤ grade 1. Restart at same dose.
Grade 3 Thrombocytopenia (platelets <50, ≥ 25 x109/L)	Hold everolimus and ribociclib until recovery to ≤ grade 1 (platelets ≥ 75 x109/L). If resolution occurs ≤ 7 days, re-start at same dose level prior to interruption.<br>If resolution takes > 7 days, or if event recurs within 28 days, hold everolimus and ribociclib until recovery to ≤ grade 1 or baseline grade / value and reintroduce the medications at one dose level lower, if available.

Grade 4 Thrombocytopenia (platelets < 25 x10^9/L)	Hold everolimus, ribociclib, and letrozole until recovery to ≤ grade 1 (platelets ≥ 75 x10^9/L) then reintroduce the medications at one dose level lower, if available. If grade 4 toxicity recurs discontinue ribociclib.
Grade 3 Neutropenia (neutrophils <1, ≥0.5 x10^9/L)	Hold everolimus and ribociclib until recovery to ANC ≥ 1.0 x 10^9/L. If resolution occurs ≤ 7 days, everolimus and ribociclib should be re-started at the dose level prior to interruption.<br>If resolution takes > 7 days, or if event recurs within 28 days, hold everolimus and ribociclib until recovery to ANC ≥ 1.0 x 10^9/L reintroduce the medications at one dose level lower, if available.
Grade 4 Neutropenia (neutrophils < 0.5 x10^9/L)	Hold everolimus and ribociclib until recovery to ANC ≥ 1.0 x 10^9/L then reintroduce the medications at one dose level lower, if available.
Grade 3 febrile neutropenia (not life-threatening)	Hold Everolimus and ribociclib until resolution of fever and recovery ANC ≥ 1.0 x 10^9/L. Re-started at the dose level prior to interruption.<br>If febrile neutropenia recurs, discontinue ribociclib.
Grade 4 febrile neutropenia (life-threatening)	Remove from study.
Grade 3 Anemia (<8.0 g/dL; <4.9 mmol/L)	If asymptomatic, monitor closely; patient may continue on study medications.<br>If symptomatic, transfuse patient as indicated; patient may continue on study medications. If the patient requires additional transfusions at more than one time point they may continue on study at the discretion of the treating physician.
Grade 4 Anemia (Life-threatening)	Remove from study.
Any hematological or non-hematological toxicity requiring interruption for ≥ 28 days	Remove from study.

5.2.1 DOSE MODIFICATION IN CASE OF HEPATIC TOXICITY

Table 9. Criteria for dose-modification in case of hepatic toxicity (Bilirubin, ALT, AST)

**TOTAL BILIRUBIN without ALT/AST increase above baseline value**	
Grade1 (>ULN – 1.5 x ULN)<br>Confirmed 48-72 hours later	Maintain dose level with LFTs monitored every two weeks
Grade 2 (>1.5 – 3.0 ULN)	Hold ribociclib and everolimus.<br>If resolved to ≤ grade 1 in < 21 days, then restart same dose. If resolved to ≤ grade 1 in > 21 days, then restart at dose level lower if available. Repeat liver enzymes and bilirubin twice weekly for 2 weeks after dose resumption.<br>If toxicity recurs after 2 dose reductions, discontinue ribociclib and everolimus.
Grade 3 (>3.0 – 10.0 x ULN)	Hold ribociclib and everolimus.<br>If resolved to ≤ grade 1 in < 28 days, then lower one dose level. Repeat liver enzymes and bilirubin twice weekly for 2 weeks after dose resumption.<br>If resolved to ≤ grade 1 in > 28 days or toxicity recurs discontinue ribociclib.

Grade 4 (> 10.0 x ULN)	Discontinue ribociclib and everolimus.
Confounding factors and/or alternative causes for increase of total bilirubin would be excluded before dose interruption/reduction. They include but are not limited to: evidence of obstruction, such as elevated ALP and GGT typical of gallbladder or bile duct disease, hyper-bilirubinemia due to indirect component only due to hemolysis or Gilbert Syndrome, pharmacologic treatment, viral hepatitis, alcoholic or autoimmune hepatitis, and other hepatotoxic drugs.	
**AST or ALT without bilirubin elevation >2 x ULN**	
Same grade as baseline or increase from baseline grade 0 to grade 1 (confirmed 48 to 72 hours later)	No dose adjustment with LFTs monitored per protocol if same grade as baseline. If increase from grade 0 to grade 1, monitor every two weeks. If the patient is stable at grade 1, start monthly monitoring.
Increase from baseline grade 0 or 1 to grade 2 (>3.0 – 5.0 x ULN)	Hold ribociclib and everolimus.<br>If resolved to ≤ grade 1 in < 21 days, then restart same dose. If resolved to ≤ grade 1 in > 21 days, then restart at dose level lower if available. Repeat liver enzymes and bilirubin twice weekly for 2 weeks after dose resumption. If toxicity recurs after 2 dose reductions or recovery to < baseline is >28 days, discontinue ribociclib
Increase from baseline grade 0 or to grade 3 (>5.0 – 20.0 x ULN)	Hold ribociclib and everolimus.<br>If resolved to ≤ baseline value, then lower one dose level. Repeat liver enzymes and bilirubin twice weekly for 2 weeks after dose resumption.<br>If resolved to ≤ baseline in > 28 days or toxicity recurs discontinue ribociclib.
Increase from baseline grade 2 to grade 3 (>5.0 – 20.0 x ULN)	Hold ribociclib and everolimus.<br>If resolved to ≤ baseline value, then lower one dose level. Repeat liver enzymes and bilirubin twice weekly for 2 weeks after dose resumption.<br>If resolved to ≤ baseline in > 28 days or toxicity recurs discontinue ribociclib.
Grade 4 (>20.0 x ULN)	Discontinue ribociclib and everolimus.
AST or ALT and concurrent Bilirubin<br>For patients with normal ALT or AST or total bilirubin at baseline: AST or ALT > grade 2 combined with total bilirubin >2 x ULN without evidence of cholestasis<br>OR<br>For patients with elevated ALT or AST or total bilirubin at baseline: baseline (AST or ALT > 2 x baelsine and >3.0 x ULN) OR (AST or ALT 8.0 x ULN) whichever is lower-combined with (total bilirubin 2 x baseline AND >2 x ULN)	Discontinue ribociclib and everolimus.

Increase in transaminases combined with total bilirubin (TBIL) increase may be indicative of drug-induced liver injury (DILI), and should be considered as clinically important events.

The threshold for potential DILI may depend on the patient's baseline AST/ALT and TBIL value; patients meeting any of the following criteria will require further follow-up as outlined below:

- For patients with normal ALT or AST or TBIL value at baseline: AST or ALT > 3.0 x ULN combined with TBIL > 2.0 x ULN
- For patients with elevated AST or ALT or TBIL value at baseline: [AST or ALT > 2 x baseline AND > 3.0 x ULN] OR [AST or ALT > 8.0 x ULN], whichever is lower, combined with [TBIL> 2 x baseline AND > 2.0 x ULN]

Medical review needs to ensure that liver test elevations are not caused by cholestasis, defined as: ALP elevation > 2.0 x ULN with R value < 2 in patients without bone metastasis, or elevation of ALP liver fraction in patients with bone metastasis.

Note: (The R value is calculated by dividing the ALT by the ALP, using multiples of the ULN for both values. It denotes the relative pattern of ALT and/or ALP elevation is due to cholestatic or hepatocellular liver injury or mixed type injury)

In the absence of cholestasis, these patients should be immediately discontinued from study drug treatment, and repeat LFT testing as soon as possible, preferably within 48 hours from the awareness of the abnormal results. The evaluation should include laboratory tests, detailed history, physical assessment and the possibility of liver metastasis or new liver lesions, obstructions/compressions, etc.

Hepatic toxicity monitoring includes the following LFTs: albumin, ALT, AST, total bilirubin, direct and indirect bilirubin, alkaline phosphatase (fractionated if alkaline phosphatase is grade 2 or higher), creatine kinase, prothrombin time (PT/INR) and GGT (Table 9). For patients with Gilbert Syndrome: total and direct bilirubin must be monitored, intensified monitoring applies to changes in direct bilirubin only.

Close observation is recommended in case of AST, ALT, and/or bilirubin increase requiring dose interruption, which involves:

- Repeating liver enzyme and serum bilirubin tests **two or three times weekly**. Frequency of re-testing can decrease to once a week or less if abnormalities stabilize or return to normal values.
- Obtaining a more detailed history of current symptoms.
- Obtaining a more detailed history of prior and/or concurrent diseases, including history of any pre-existing liver conditions or risk factors.
- Obtaining a history of concomitant drug use (including non-prescription medications, herbal and dietary supplements), alcohol use, recreational drug use, and special diets.
- Ruling out acute viral hepatitis types A, B, C, D, and E; hepatotropic virus infections (CMV, EBV or HSV); autoimmune or alcoholic hepatitis; NASH; hypoxic/ischemic hepatopathy; and biliary tract disease.
- Obtaining a history of exposure to environmental chemical agents.
- Obtaining additional tests to evaluate liver function, as appropriate (e.g., INR, direct bilirubin).
- Considering gastroenterology or hepatology consultations.
- Assessing cardiovascular dysfunction or impaired liver oxygenation, including hypotension or right heart failure as possible etiologies for liver dysfunction.

Liver biopsy as clinically indicated to assess pathological change and degree of potential liver injury.

All cases confirmed on repeat testing meeting the laboratory criteria defined above, with no other alternative cause for LFT abnormalities identified, should be considered as "medically significant", thus met the

definition of SAE (Section 7.02), and reported as SAE using the term “potential drug-induced liver injury”. All events should be followed up with the outcome clearly documented.

5.2.2 DOSE MODIFICATIONS IN CASE OF QT PROLONGATION

Table 10. Criteria for dose-modification in case of QT prolongation

Grade	Dose modification
For all grades	1. Check the quality of the ECG and the QT value and repeat if needed.<br>2. Perform analysis of serum electrolytes (K+, Ca++, Phos, Mg++). If outside of the normal range,, interrupt ribociclib administration, correct with supplements or appropriate therapy as soon as possible, and repeat electrolytes until documented as normal.<br>3. Review concomitant medication usage for the potential to inhibit CYP3A4 and/or to prolong the QT interval.<br>4. Check compliance with correct dose and administration of ribociclib.<br><br>Consider collecting a time matched PK sample; record date and time of last study drug intake.
Grade 1<br>QTc 450-480 ms	Perform steps 1-4 as directed in “For all grades.” No dose adjustment required
Grade 2<br>QTc 481-500 ms	Interrupt ribociclib. Perform steps 1-4 as directed in “For all grades.”<br>• Perform a repeat ECG **within** one hour **of** the first QTcF of ≥ 481ms.<br>• Repeat ECG as clinically indicated until the QTcF returns to < 481 ms. Restart ribociclib **with dose reduced by 1 dose level**.<br>• If QTcF ≥ 481 ms recurs, ribociclib should be reduced **again** by 1 dose level.<br>• Repeat ECGs 7 days and 14 days after dose resumption (then as clinically indicated) for any patient who had therapy interrupted due to QTcF ≥481 ms.
Grade 3<br>QTc ≥501 ms on at least two separate ECGs	Interrupt ribociclib. Perform steps 1-4 as directed in “For all grades.”<br>**Transmit ECG immediately and confirm prolongation/abnormalities with central assessment (if applicable).**<br>• Perform a repeat ECG within one hour of the first QTcF of ≥ 501 ms.<br>• If QTcF remains ≥ 501 ms, consult with a cardiologist (or qualified specialist) and repeat cardiac monitoring as indicated until the QTcF returns to <481 ms.<br>• If QTcF returns to < 481 ms, ribociclib should be reduced by 1 dose level (please refer to the dosing schedule table)<br>• If QTcF remains to ≥ 481 ms after performing steps 1-4 as directed in “For all grades,” discontinue ribociclib.<br>• Repeat ECGs 7 days and 14 days after dose resumption **(then as clinically indicated)** for any patient who has therapy interrupted due to QTcF ≥ 501 ms<br>• If QTcF of ≥ 501 ms recurs, discontinue ribociclib.
Grade 4<br>QT/QTc ≥501 or >60 ms change from baseline<br>and<br>Torsades de pointes or polymorphic ventricular tachycardia, or signs/symptoms of serious arrhythmia	Discontinue ribociclib. Perform steps 1-4 as directed in “For all grades.”<br>• Obtain local cardiologist consultation (or qualified specialist) and repeat cardiac monitoring as indicated until the QtcF returns to <481ms

5.2.3 MANAGEMENT OF STOMATITIS/ORAL MUCOSITIS/MOUTH ULCERS

Patients with a clinical history of stomatitis/mucositis/mouth ulcers and those with gastrointestinal morbidity associated with mouth/dental infections, irritation of esophageal mucosa e.g. gastroesophageal reflux disease (GERD) and pre-existing stomatitis/mucositis must be monitored even more closely. Patients should be instructed to report the first onset of buccal mucosa irritation/reddening to their study physician immediately.

Stomatitis/oral mucositis/mouth ulcers due to everolimus should be treated using local supportive care. Please note that investigators in earlier trials have described the oral toxicities associated with everolimus as mouth ulcers, rather than mucositis or stomatitis. If your examination reveals mouth ulcers rather than a more general inflammation of the mouth, please classify the adverse event as such.

Please follow the paradigm below for treatment of stomatitis/oral mucositis/mouth ulcers:

1. For mild toxicity (Grade 1), use conservative measures such as non-alcoholic mouth wash or salt water (0.9%) mouth wash several times a day until resolution.
2. For more severe toxicity (Grade 2 in which case patients have pain but are able to maintain adequate oral alimentation, or Grade 3 in which case patients cannot maintain adequate oral alimentation), the suggested treatments are topical analgesic mouth treatments (i.e., local anesthetics such as benzocaine, butyl aminobenzoate, tetracaine hydrochloride, menthol, or phenol) with or without topical corticosteroids, such as triamcinolone oral paste 0.1% (Kenalog in Orabase®).
3. Agents containing hydrogen peroxide, iodine, and thyme derivatives may tend to worsen mouth ulcers. It is preferable to avoid these agents.
4. Antifungal agents must be avoided unless a fungal infection is diagnosed. In particular, systemic imidazole antifungal agents (ketoconazole, fluconazole, itraconazole, etc.) should be avoided in all patients due to their strong inhibition of everolimus metabolism, therefore leading to higher everolimus exposures. Therefore, topical antifungal agents are preferred if an infection is diagnosed. Similarly, antiviral agents such as acyclovir should be avoided unless a viral infection is diagnosed.

Note: Stomatitis/oral mucositis should be appropriately graded using the functional grading given on the NCI-CTC for adverse events, version 4.0.

5.2.4 MANAGEMENT OF SKIN TOXICITY

For patients with grade 1 toxicity, no specific supportive care is usually needed or indicated. Rash must be reported as an AE. Patients with grade 2 or higher toxicity may be treated with the following suggested supportive measures at the discretion of the investigator: oral minocycline, topical tetracycline, topical clindamycin, topical silver sulfadiazine, diphenhydramine, oral prednisolone (short course), topical corticosteroids, or pimecrolimus.

5.2.5 MANAGEMENT OF DIARRHEA

Appearance of grade 1-2 diarrhea attributed to study drug toxicity may be treated with supportive care such as loperamide, initiated at the earliest onset.

5.2.6 MANAGEMENT OF HYPERLIPIDEMIA AND HYPERGLYCEMIA

Treatment of hyperlipidemia should take into account the pre-treatment status and dietary habits of the patient. Grade 2 or higher hypercholesterolemia (>300 mg/dL or 7.75 mmol/L) or grade 2 hypertriglyceridemia or higher (>2.5x upper normal limit) should be treated with a 3-hydroxy-3-methyl-glutaryl (HMG)-CoA reductase inhibitor (e.g. atorvastatin, pravastatin, fluvastatin) or appropriate triglyceride-lowering medication, in addition to diet.
Note: Concomitant therapy with fibrates and an HMG-CoA reductase inhibitor is associated with an increased risk of a rare but serious skeletal muscle toxicity manifested by rhabdomyolysis, markedly elevated creatine phosphokinase (CPK) levels and myoglobinuria, acute renal failure and sometimes death. The risk versus benefit of using this therapy should be determined for individual patients based on their risk of cardiovascular complications of hyperlipidemia
Hyperglycemia has been reported in clinical trials. Monitoring of fasting serum glucose is recommended prior to the start of everolimus therapy and periodically thereafter. Optimal glycemic control should be achieved before starting a patient on study drug.

5.2.7 MANAGEMENT OF NON-INFECTIOUS PNEUMONITIS

Non-infectious pneumonitis is a class effect of rapamycin derivatives. Cases of non-infectious pneumonitis (including interstitial lung disease) have also been described in patients taking everolimus. Some of these have been severe and on rare occasions, a fatal outcome was observed.

A diagnosis of non-infectious pneumonitis should be considered in patients presenting with non-specific respiratory signs and symptoms such as hypoxia, pleural effusion, cough or dyspnea, and in whom infectious, neoplastic and other non-medicinal causes have been excluded by means of appropriate investigations. Opportunistic infections such as PJP should be ruled out in the differential diagnosis of non-infectious pneumonitis. Patients should be advised to report promptly any new or worsening respiratory symptoms.

Patients who develop radiological changes suggestive of non-infectious pneumonitis and have few or no symptoms may continue everolimus therapy without dose alteration. If symptoms are moderate (Grade 2), interrupt everolimus until recovery to ≤ Grade 1. The use of corticosteroids may be indicated. Everolimus should be reintroduced at a reduced dose after recovery to Grade 1 (Table 11). For cases where symptoms of non-infectious pneumonitis are severe (Grade 3), everolimus therapy should be discontinued and the use of corticosteroids may be indicated until clinical symptoms resolve. Therapy with everolimus may be re-initiated at a reduced dose depending on the individual clinical circumstances (Table 11).

Table 11. Management of Non-Infectious Pneumonitis

Worst Grade Pneumonitis	Required Investigations	Management of Pneumonitis	Everolimus Dose Adjustment	Ribociclib Dose Adjustment
Grade 1	CT scans with lung windows	No specific therapy is required	Administer 100% of everolimus dose.	No dose adjustment required. Initiate appropriate medical therapyand monitor as clinically indicated.
Grade 2	CT scan with lung windows and consider pulmonary function testing including: spirometry, D_LCO, and room air O_2 saturation at rest. Repeat each subsequent Cycle until return to baseline. Consider bronchoscopy *	Symptomatic only. Consider corticosteroids if cough is troublesome. Consider pulmonary consult.	Rule out infection and hold Everolimus until recovery to ≤ Grade 1. Re-initiate Everolimus at one dose level lower if available. Patients will be withdrawn from the study if they fail to recover to ≤ Grade 1 within 28 days.	Interrupt ribociclib dose until recovery to Grade ≤1, then resume ribociclib at the next lower dose level. An individualized bene/fit risk assessment should be perfomed before resuming ribociclib.
Grade 3	CT scan with lung windows and pulmonary function testing including: spirometry, D_LCO, and room air O_2 saturation at rest; Repeat each subsequent Cycle until return to ≤ Grade 1. Bronchoscopy is recommended *	Pulmonary consult. Consider corticosteroids if infective origin is ruled out. Taper as medically indicated.	Rule out infection and hold Everolimus until recovery to ≤ Grade 1. Re-initiate Everolimus at one dose level lower if available. Patients will be withdrawn from the study if they fail to recover to ≤ Grade 1 within 28 days.	Discontinue ribociclib.
Grade 4	CT scan with lung windows and required pulmonary function testing includes: spirometry, DLCO, and room air O_2 saturation at rest. Repeat each subsequent Cycle until return to baseline. Bronchoscopy is recommended *.	Pulmonary consult. Consider corticosteroids if infective origin is ruled out. Taper as medically indicated.	Rule out infection and discontinue treatment.	Discontinue ribociclib.

*A bronchoscopy with biopsy and/or bronchoalveolar lavage is recommended.

5.2.8 MANAGEMENT OF HEPATITIS REACTIVATION / FLARE

Reactivation of Hepatitis B (HBV) has been observed in patients with cancer receiving chemotherapy (Yeo, et al 2004). Sporadic cases of Hepatitis B reactivation have also been seen in this setting with everolimus. Use of antivirals during anti-cancer therapy has been shown to reduce the risk of Hepatitis B virus reactivation and associated morbidity and mortality (Loomba, et al. 2008). A detailed assessment of Hepatitis B/C medical history and risk factors must be done for all patients at screening, with testing performed prior to the first dose of everolimus.

Monitoring and prophylactic treatment for hepatitis B reactivation

Table 12 provides detail of monitoring and prophylactic therapy according to the screening results of viral load and serologic markers testing.

Table 12. Action to be taken based on screening hepatitis B results

Test	Result	Result	Result	Result	Result
HBV-DNA	+	+ or -	-	-	-
HBsAg	+ or -	+	-	-	-
HBsAb	+ or -	+ or -	+<br>and no prior HBV vaccination	+ or -	-<br>or + with prior HBV vaccination
HBcAb	+ or -	+ or -	+ or -	+	-
Recommendation	Prophylaxis treatment should be started 1-2 weeks prior to first dose of Everolimus<br><br>Monitor HBV-DNA approximately every 4-8 weeks		No prophylaxis<br><br>Monitor HBV-DNA approximately every 3-4 weeks		No specific action

Antiviral prophylaxis therapy should continue for at least 4 weeks after last dose of Everolimus. For HBV reactivation definition and management guidelines, see Table 13.

Table 13. Guidelines for the management of hepatitis B reactivation

HBV reactivation (with or without clinical signs and symptoms)*	
For patients with baseline results:<br>Positive HBV-DNA<br>OR<br>positive HBsAg<br>----------------------------------<br>**reactivation is defined as:**<br>[Increase of 1 log in HBV-DNA relative to baseline HBV-DNA value OR new appearance of measurable HBV-DNA]	**Treat**: Start a second antiviral medication AND<br>**Interrupt** Everolimus and ribociclib administration until resolution:<br>• ≤ baseline HBV-DNA levels<br>**If resolution occurs within ≤ 28 days**, everolimus and ribociclib should be re-started at one dose lower, if available (see Table 7 for dose levels available). If the patient is already receiving the lowest dose of everolimus and ribociclib according to the protocol, the patient should restart at the same dose after resolution. Both antiviral therapies should continue at least 4 weeks after last dose.<br>**If resolution occurs > 28 days** Patients should discontinue everolimus and ribociclib but continue both antiviral therapies at least 4 weeks after last dose of everolimus.

For patients with baseline results:<br><br>Negative HBV-DNA **and** HBsAg<br><br>AND<br><br>**[**Positive HBsAb (with no prior history of vaccination against HBV), **OR** positive HBcAb**]**<br><br>----------------------------------<br><br>**Reactivation is defined as:** New appearance of measurable HBV-DNA	**Treat :** Start first antiviral medication AND<br><br>**Interrupt** everolimus and ribociclib administration until resolution:<br><br>• ≤ undetectable (negative) HBV-DNA levels<br><br>**If resolution occurs within ≤ 28 days**, everolimus and ribociclib should be re-started at one dose lower, if available (see Table 7 for dose levels available). If the patient is already receiving the lowest dose of everolimus according to the protocol, the patient should restart at the same dose after resolution. Antiviral therapy should continue at least 4 weeks after last dose of everolimus and ribociclib.<br><br>**If resolution occurs > 28 days** Patients should discontinue everolimus and ribociclib but continue antiviral therapy at least 4 weeks after last dose of everolimus and ribociclib.

* All reactivations of HBV are to be recorded as grade 3 (e.g. CTCAE Version 4.03 - Investigations/Other: Viral Reactivation), unless considered life threatening by the investigator, in which case they should be recorded as grade 4. Date of viral reactivation is the date on which the rise or reappearance of HBV-DNA was recorded.

5.2.9 MONITORING FOR HEPATITIS C FLARE

The following two categories of patients should be monitored every 4–8 weeks for HCV flare:

- Patients with detectable HCV RNA-PCR test at screening.
- Patients known to have a history of HCV infection, despite a negative viral load test at screening (including those that were treated and are considered ‘cured’)

For definitions of HCV flare and actions to be taken in the event of a flare, please refer Table 14.

Table 14. Guidelines for the management of hepatitis C flare

Baseline results	HCV flare definition*	HCV flare management
Detectable HCV-RNA	> 2 $\log_{10}$ IU/mL increase in HCV-RNA<br><br>AND<br><br>ALT elevation > 5 x ULN or 3 x baseline level, whichever is higher.	Discontinue everolimus
Knowledge of past hepatitis C infection with no detectable HCV-RNA	New appearance of detectable HCV-RNA<br><br>AND<br><br>ALT elevation > 5 x ULN or 3 x baseline level, whichever is higher.	Discontinue everolimus

* All flares of HCV are to be recorded as grade 3 (e.g. CTCAE Version 4.03- Investigations - Other: Viral Flare), unless considered life threatening by the investigator; in which case they should be recorded as grade 4. Date of viral flare is the date on which both the clinical criteria described above were met. (e.g., for a patient whose HCV-RNA increased by 2 logs on 01 JAN 2011 and whose ALT reached > 5 x ULN on 22 JAN 2011, the date of viral flare is 22 JAN 2011).

5.2.10 RENAL IMPAIRMENT

Insufficient data are available to provide a dosage recommendation for ribociclib in patients with renal impairment. Patients with baseline renal impairment are excluded from the study (serum creatinine > 1.5 or creatinine clearance <50 mL/min). For patients who experience renal impairment of grade 2 or higher during the treatment period hold Ribociclib and Everolimus until recovery to ≤ grade 1 or baseline. If resolution occurs within 28 days, restart study medications at dose level prior to interruption. For patients who experience renal impairment of grade 2 or higher during the treatment period, hold Ribociclib and Everolimus until recovery to ≤ grade 1. If resolution occurs within 28 days, restart study medications at dose level prior to interruption. If renal impairment of grade 2 or higher recurs, hold Ribociclib and Everolimus until recovery to ≤ grade 1 and reintroduce the medications at one dose level lower, if available. Patients will be withdrawn from the study if they fail to recover to ≤ grade 1 or baseline within 28 days.

5.2.11 ANGIOEDEMA WITH CONCOMITANT USE OF ANGIOTENSIN-CONVERTING ENZYME (ACE) INHIBITORS

Patients taking concomitant ACE inhibitor therapy may be at increased risk for angioedema (e.g. swelling of the airways or tongue, with or without respiratory impairment).

6 ASSESSMENTS

6.1 HBV/HCV ASSESSMENTS

A detailed assessment of hepatitis B/C medical history and risk factors must be done for all patients at screening. Patients with positive hepatitis B and/or hepatitis C test results must begin a course of antivirals 1-2 weeks prior to the first dose of everolimus (as outlined in section 3.4.13).
Testing for hepatitis B viral load and serologic markers: HBV-DNA, HBsAg, HBsAb, and HBc Ab and HCV RNA PCR are required at screening for all patients in the following risk categories:

- All patients who currently live in (or have lived in) Asia, Africa, Central and South America, Eastern Europe, Spain, Portugal, and Greece. [http://wwwnc.cdc.gov/travel/yellowbook/2010/chapter-2/hepatitis-b.aspx#849]
- Patients with any of the following risk factors:
  - known or suspected past hepatitis B infection,
  - blood transfusion(s) prior to 1990,
  - current or prior IV drug users,
  - current or prior dialysis,
  - household contact with hepatitis B infected patient(s),
  - current or prior high-risk sexual activity,
  - mother known to have hepatitis B
  - History suggestive of hepatitis B infection, e.g., dark urine, jaundice, right upper quadrant pain.
- Additional patients at the discretion of the investigator

Testing for hepatitis C should be performed using quantitative RNA-PCR at screening for all patients in the following risk categories:

- known or suspected past hepatitis C infection (including patients with past interferon 'curative' treatment),

- blood transfusions prior to 1990,
- current or prior IV drug users,
- current or prior dialysis,
- household contact of hepatitis C infected patient(s),
- current or prior high-risk sexual activity

At the discretion of the investigator, additional patients may also be tested for hepatitis C.

6.2 EFFICACY ASSESSMENTS

6.2.1 ANTITUMOR EFFECT – SOLID TUMORS

For the purposes of this study, patients should be re-evaluated for response after the first 2 cycles (8 weeks). In addition, confirmatory scans should be obtained after cycle # 4 and 6 and then every 3 cycles (12 weeks) following initial documentation of objective response.
Response and progression will be evaluated in this study using the new international criteria proposed by the revised Response Evaluation Criteria in Solid Tumors (RECIST) Committee guidelines (version 1.1) [*Eur J Ca* 45:228-47, 2009]. Changes in the largest diameter (unidimensional measurement) of the tumor lesions and the shortest diameter in the case of malignant lymph nodes are used in the RECIST criteria.

Radiologists at the MD Anderson Cancer Center have developed a multimedia structured reporting system, called ViSion, which will be used to capture the key images and metrics recorded by a radiologist for tumor response assessment. The captured images are tagged by a radiologist with metadata referenced to the SNOMED-CT ontology to indicate the anatomical location and radiological observation/diagnosis of each finding. The ViSion system displays serial image findings linked in graphical disease timelines. Target and non-target lesions may be designated by a radiologist from which the calculation and display of disease response criteria (e.g., RECIST) is automated. The radiologist's actual image analysis is performed using the institution's FDA-cleared Philips iSite picture archiving and communications system (PACS) display that is enabled with electronic calipers – ViSion only captures the annotated key images and metrics for subsequent storage, analysis and display. The ViSion database is housed within theinstitutional firewall on an MD Anderson server, and the system is HIPAA-compliant requiring institutional passwords for controlled access.

6.2.2 DEFINITIONS

Evaluable for toxicity. All patients will be evaluable for toxicity from the time of their first treatment with everolimusand letrozole, ± ribociclib.

Evaluable for objective response. Only those patients who have measurable disease present at baseline, have received at least one cycle of therapy, and have had their disease re-evaluated will be considered evaluable for response. These patients will have their response classified according to the definitions stated below. (Note: Patients who exhibit objective disease progression prior to the end of cycle 1 will also be considered evaluable.)

6.2.3 DISEASE PARAMETERS

Measurable disease. Measurable lesions are defined as those that can be accurately measured in at least one dimension (longest diameter to be recorded) as $\geq$20 mm with conventional techniques (CT, MRI, x-ray) or as $\geq$10 mm with spiral CT scan. All tumor measurements must be recorded in millimeters (or decimal fractions of centimeters).

Note: Tumor lesions that are situated in a previously irradiated area might or might not be considered measurable. Tumors within a previously irradiated field will be designated as "non-target" lesions, unless progression is documented or a biopsy is obtained to confirm persistence at least 90 days following completion of radiation therapy.

Malignant lymph nodes. To be considered pathologically enlarged and measurable, a lymph node must be ≥15 mm in short axis when assessed by CT scan (CT scan slice thickness recommended to be no greater than 5 mm). At baseline and in follow-up, only the short axis will be measured and followed.

Non-measurable disease. All other lesions (or sites of disease), including small lesions (longest diameter <20 mm with conventional techniques or <10 mm using spiral CT scan), are considered non-measurable disease. Bone lesions, leptomeningeal disease, ascites, pleural/pericardial effusions, lymphangitis cutis/pulmonis, inflammatory breast disease, abdominal masses (not followed by CT or MRI), and cystic lesions are all non-measurable.

Note: Cystic lesions that meet the criteria for radiographically defined simple cysts should not be considered as malignant lesions (neither measurable nor non-measurable) since they are, by definition, simple cysts. "Cystic lesions" thought to represent cystic metastases can be considered as measurable lesions, if they meet the definition of measurability described above. However, if non-cystic lesions are present in the same patient, these are preferred for selection as target lesions.

Target lesions. All measurable lesions up to a maximum of 2 lesions per organ and 5 lesions in total, representative of all involved organs, should be identified as **target lesions** and recorded and measured at baseline. Target lesions should be selected on the basis of their size (lesions with the longest diameter) and their suitability for accurate repeated measurements (either by imaging techniques or clinically). A sum of the longest diameter (LD) for all target lesions will be calculated and reported as the baseline sum LD. The baseline sum LD will be used as reference by which to characterize the objective tumor response.

Non-target lesions. All other lesions (or sites of disease) including any measurable lesions over and above the 5 target lesions should be identified as **non-target lesions** and should also be recorded at baseline. Measurements of these lesions are not required, but the presence or absence of each should be noted throughout follow-up.

Methods for Evaluation of Measurable Disease

All measurements should be taken and recorded in metric notation using a ruler or calipers. All baseline evaluations should be performed as closely as possible to the beginning of treatment and never more than 4 weeks before the beginning of the treatment.

The same method of assessment and the same technique should be used to characterize each identified and reported lesion at baseline and during follow-up. Imaging-based evaluation is preferred to evaluation by clinical examination when both methods have been used to assess the antitumor effect of a treatment.

Clinical lesions Clinical lesions will only be considered measurable when they are superficial (e.g., skin nodules and palpable lymph nodes) and ≥10 mm diameter as assessed using calipers. In the case of skin lesions, documentation by color photography, including a ruler to estimate the size of the lesion, is recommended.

Chest x-ray Lesions on chest x-ray are acceptable as measurable lesions when they are clearly defined and surrounded by aerated lung. However, CT is preferable.

Conventional CT and MRI This guideline has defined measurability of lesions on CT scan based on the assumption that CT slice thickness is 5mm or less. If CT scans have slice thickness greater than 5 mm, the minimum size for a measurable lesion should be twice the slice thickness. MRI is also acceptable. As with CT, if an MRI is performed, the technical specifications of the scanning sequences used should be optimized for the evaluation of the type and site of disease.

The cytological confirmation of the neoplastic origin of any effusion that appears or worsens during treatment when the measurable tumor has met criteria for response or stable disease is mandatory to differentiate between response or stable disease (an effusion may be a side effect of the treatment) and progressive disease.

6.2.4 RESPONSE CRITERIA

6.2.4.1 EVALUATION OF TARGET LESIONS

Complete Response (CR):	Disappearance of all target lesions. Any pathologic lymph nodes (whether target or non-target) must have reduction in short axis to <10 mm
Partial Response (PR):	At least a 30% decrease in the sum of the longest diameter (LD) of target lesions, taking as reference the baseline sum LD
Progressive Disease (PD):	At least a 20% increase in the sum of the LD of target lesions, taking as reference the smallest sum LD recorded since the treatment started or the appearance of one or more new lesions. In addition to the relative increase of 20%, the sum LD must also demonstrate an absolute increase of at least 5 mm.
Stable Disease (SD):	Neither sufficient shrinkage to qualify for PR nor sufficient increase to qualify for PD, taking as reference the smallest sum LD since the treatment started.

6.2.4.2 EVALUATION OF NON-TARGET LESIONS

Complete Response (CR):	Disappearance of all non-target lesions and normalization of tumor marker level. All lymph nodes must be non-pathological in size (<10 mm short axis). **Note:** If tumor markers are initially above the upper normal limit, they must normalize for a patient to be considered in complete clinical response.
Non-CR/Non-PD:	Persistence of one or more non-target lesion(s) and/or maintenance of tumor marker level above the normal limits.
Progressive Disease (PD):	Appearance of one or more new lesions and/or unequivocal progression of existing non-target lesions<br>Although a clear progression of “non-target” lesions only is exceptional, the opinion of the treating physician should prevail in such circumstances, and the progression status should be confirmed at a later time by the review panel (or Study PI).

6.2.5 EVALUATION OF BEST OVERALL RESPONSE

The best overall response is the best response recorded from the start of the treatment until disease progression/recurrence (taking as reference for progressive disease the smallest measurements recorded since

the treatment started). The patient's best response assignment will depend on the achievement of both measurement and confirmation criteria.

Table 15. Evaluation for Patients with Measurable Disease (i.e., Target Disease)

Target Lesions	Non-Target Lesions	New Lesions	Overall Response	Best Response for this Category Also Requires*:
CR	CR	No	CR	≥4 wks. Confirmation**
CR	Non-CR/Non-PD	No	PR	≥4 wks. Confirmation**
PR	Non-PD	No	PR	
SD	Non-PD	No	SD	documented at least once ≥4 wks. from baseline
PD	Any	Yes or No	PD	no prior SD, PR or CR
Any	PD***	Yes or No	PD	
Any	Any	Yes	PD	

* See RECIST v1.1 manuscript for further details on what is evidence of a new lesion.

** Only for non-randomized trials with response as primary endpoint.

*** In exceptional circumstances, unequivocal progression in non-target lesions may be accepted as disease progression.

Note: Patients with a global deterioration of health status requiring discontinuation of treatment without objective evidence of disease progression at that time should be reported as "*symptomatic deterioration*". Every effort should be made to document the objective progression even after discontinuation of treatment.

6.2.6 DURATION OF RESPONSE

Duration of overall response: The duration of overall response is measured from the time measurement criteria are met for CR or PR (whichever is first recorded) until the first date that recurrent or progressive disease is objectively documented (taking as reference for progressive disease the smallest measurements recorded since the treatment started).

The duration of overall CR is measured from the time measurement criteria are first met for CR until the first date that recurrent disease is objectively documented.

Duration of stable disease: Stable disease is measured from the start of the treatment until the criteria for progression are met, taking as reference the smallest measurements recorded since the treatment started, including the baseline measurements.

Patients achieving a complete response, once confirmed, are permitted to stop study medication. Subsequent follow-up will be at the discretion of the treating physician.

6.2.7 OTHER EFFICACY PARAMETERS

Duration of disease control, time to disease progression, 6 month progression free survival and overall survival will be evaluated.

7 SAFETY ASSESSMENTS

Safety assessments will consist of monitoring and recording adverse events present of grade 2 or higher and possibly related to any of the study drugs. Adverse events will be collected starting at the first dose of any study drug until 30 days after the last dose. See Tables 5 and 6 evaluation and visit schedule.

Safety and tolerability will be assessed according to the NIH/NCI CTCAEv4.03.

7.1 ADVERSE EVENTS

Information about all adverse events, whether volunteered by the subject, discovered by investigator questioning, or detected through physical examination, laboratory test or other means, will be collected and recorded and followed as appropriate.

An adverse event is the appearance or worsening of any undesirable sign, symptom, or medical condition occurring after starting the study drug even if the event is not considered to be related to study drug. Medical conditions/diseases present before starting study drug are only considered adverse events if they worsen after starting study drug. Abnormal laboratory values or test results constitute adverse events only if they induce clinical signs or symptoms, are considered clinically significant, or require therapy.

The occurrence of adverse events should be sought by non-directive questioning of the patient at each visit during the study. Adverse events also may be detected when they are volunteered by the patient during or between visits or through physical examination, laboratory test, or other assessments. As far as possible, each adverse event should be evaluated to determine:

1. the severity grade (mild, moderate, severe) or (grade 1-4)
2. its relationship to the study drug(s) (suspected/not suspected)
3. its duration (start and end dates or if continuing at final exam)
4. action taken (no action taken; study drug dosage adjusted/temporarily interrupted; study drug permanently discontinued due to this adverse event; concomitant medication taken; non-drug therapy given; hospitalization/prolonged hospitalization)
5. whether it constitutes a serious adverse event (SAE)

All adverse events should be treated appropriately. Such treatment may include changes in study drug treatment including possible interruption or discontinuation, starting or stopping concomitant treatments, changes in the frequency or nature of assessments, hospitalization, or any other medically required intervention. Once an adverse event is detected, it should be followed until its resolution, and assessment

should be made at each visit (or more frequently, if necessary) of any changes in severity, the suspected relationship to the study drug, the interventions required to treat it, and the outcome.

Information about common side effects already known about the investigational drug can be found in the [Investigators' Brochure]. This information should be included in the patient informed consent and should be discussed with the patient during the study as needed.

7.2 TREATMENT COMPLIANCE

Records of study medication used, dosages administered, and intervals between visits will be recorded during the study. Drug accountability will be noted and patients will be asked to return all unused study medication.

7.3 SAFETY MONITORING

The study will be monitored by the M.D. Anderson IND office and a protocol specific monitoring plan will be followed. Also, the MDACC Data Safety Monitoring Board (DSMB) will be monitoring this protocol.

7.3.1 SERIOUS ADVERSE EVENT (SAE) DEFINITION AND REPORTING TO MD ANDERSON

An adverse event or suspected adverse reaction is considered "serious" if, in the view of either the investigator or the sponsor, it results in any of the following outcomes:

- Death
- A life-threatening adverse drug experience – any adverse experience that places the patient, in the view of the initial reporter, at immediate risk of death from the adverse experience as it occurred. It does not include an adverse experience that, had it occurred in a more severe form, might have caused death.
- Inpatient hospitalization or prolongation of existing hospitalization
- A persistent or significant incapacity or substantial disruption of the ability to conduct normal life functions.
- A congenital anomaly/birth defect.

Important medical events that may not result in death, be life-threatening, or require hospitalization may be considered a serious adverse drug experience when, based upon appropriate medical judgment, they may jeopardize the patient or subject and may require medical or surgical intervention to prevent one of the outcomes listed in this definition. Examples of such medical events include allergic bronchospasm requiring intensive treatment in an emergency room or at home, blood dyscrasias or convulsions that do not result in inpatient hospitalization, or the development of drug dependency or drug abuse (21 CFR 312.32).

- Important medical events as defined above, may also be considered serious adverse events. Any important medical event can and should be reported as an SAE if deemed appropriate by the Study PI or the IND Sponsor, the MDACC IND Office.
- All events occurring during the conduct of a protocol and meeting the definition of a SAE must be reported to the IRB in accordance with the timeframes and procedures outlined in "The University of Texas M. D. Anderson Cancer Center Institutional Review Board Policy for Investigators on Reporting Serious Unanticipated Adverse Events for Drugs and Devices". Unless stated otherwise

in the protocol, all SAEs, expected or unexpected, must be reported to the IND Office, regardless of attribution (within 5 working days of knowledge of the event).

The lead site (MDACC) will utilize the electronic SAE application (eSAE) for reporting SAEs to the IND office and IRB.

7.3.2 REPORTING FOR ALL SITES

- A written report should be submitted to the Institutional Review Board (IRB) according to the requirements of the assigned IRB for patients enrolled at each site.
- Unless stated otherwise in the protocol, all SAEs, expected or unexpected, must be reported to the IND Office **within 5 working days of knowledge of the event** regardless of the attribution.
- Death or life-threatening events that are unexepected, possibly, probably or definitely related to drug must be reported **within 24 hours of knowledge of the event**.
- Serious adverse events will be captured from the time of the first protocol-specific intervention, unless the protocol states otherwise, and be reported until 30 days after the last dose of drug or protocol specific timeline. Serious adverse events must be followed until clinical recovery is complete and laboratory tests have returned to baseline, progression of the event has stabilized, or there has been acceptable resolution of the event.
- Additionally, any serious adverse events that occur after the 30 day time period, or protocol specific timeline, that are related to the study treatment must be reported to the IND Office. This may include the development of a secondary malignancy.
- All events reported the supporting company must also be reported to the IND Office.

7.3.3 REPORTING FOR NON-LEAD SITE

- SAEs will be reported to the IND Office (sponsor) on a template form that will be provided to each non-lead site. If available, a copy of all relevant examinations that have been carried out and the dates on which these examinations were performed should be attached. For laboratory results, normal ranges should be included. Patient name should be marked out and initials and study number included on all attachments.
- In case of a serious adverse event, the following actions must be undertaken by the non-lead site principal investigator: (Please note that these are in addition to the reporting that is required by the local IRB and supporting company).Complete the SAE form and upload it to the MD Anderson Box system or through secure electronic submission of SAE report(s) via e-mail to the MD Anderson IND Office safety inbox: MDACCSafetyReports@mdanderson.org
- The non-lead site will receive an electronic confirmation of receipt, and approval of the SAE report and it should be filed in the study regulatory binder at the non-lead site.
- The University of Texas M.D. Anderson Cancer Center IND Office Medical Affairs & Safety Contact Information:
  e-mail: mdaccsafetyreports@mdanderson.org

7.3.4 SAE REPORTING TO FDA

Serious adverse events will be forwarded to FDA by the IND Sponsor according to 21 CFR 312.32.

It is the responsibility of the PI and the research team at each site to ensure serious adverse events are reported according to the Code of Federal Regulations, Good Clinical Practices, the protocol guidelines, the sponsor's guidelines, and Institutional Review Board policy.

7.3.5 SAE REPORTING TO NOVARTIS

The Site PI has the obligation to report all serious adverse events per M. D. Anderson Cancer Center policy to the IRB, MD Anderson IND Office, and Novartis Pharmaceuticals Drug Safety and Epidemiology Department (DS&E).

To ensure patient safety, every SAE, regardless of suspected causality, occurring must be reported to Novartis within 24 hours of learning of its occurrence. Any SAEs experienced after this 4-week (28 days) period should only be reported to Novartis if the investigator suspects a causal relationship to the study drug. Recurrent episodes, complications, or progression of the initial SAE must be reported as follow-up to the original episode within 24 hours of the investigator receiving the follow-up information. An SAE occurring at a different time interval or otherwise considered completely unrelated to a previously reported one should be reported separately as a new event. The end date of the first event must be provided.

The investigator must assess and record the relationship of each SAE to each specific study drug (if there is more than one study drug), complete the SAE Report in English, and send the completed, signed form by email to Clinicalsafetyop.phuseh@Novartis.com within 24 hours.

The original copy of the SAE Report and the email confirmation sheet must be kept within the Trial Master File at the study site.

Follow-up information is sent to the same email as the original SAE Report Form was sent, using a new SAE Report Form stating that this is a follow-up to the previously reported SAE and giving the date of the original report. Each re-occurrence, complication, or progression of the original event should be reported as a follow-up to that event regardless of when it occurs. The follow-up information should describe whether the event has resolved or continues, if and how it was treated, whether the blind was broken or not (if applicable), and whether the patient continued or withdrew from study participation.

If the SAE is not previously documented in the everolimus IB or Package Insert (new occurrence) and is thought to be related to the Novartis study drug, a Drug Safety and Epidemiology Department associate may urgently require further information from the investigator for Health Authority reporting. Novartis may need to issue an Investigator Notification (IN), to inform all investigators involved in any study with the same drug that this SAE has been reported. Suspected Unexpected Serious Adverse Reactions (SUSARs) will be collected and reported to the competent authorities and relevant ethics committees in accordance with Directive 2001/20/EC or as per national regulatory requirements in participating countries.

All events must be reported, by email to Clinicalsafetyop.phuseh@Novartis.com within 24 hours of learning of its occurrence. This includes serious, related, labeled (expected) and serious, related, unlabeled (unexpected) adverse experiences. All deaths during treatment or within 30 days following completion of active protocol therapy must be reported within 5 working days.

Any serious adverse event occurring after the patient has provided informed consent and until 4 weeks (28 days) after the patient has stopped study participation must be reported. This includes the period in which the study protocol interferes with the standard medical treatment given to a patient (e.g. treatment withdrawal during washout period, change in treatment to a fixed dose of concomitant medication).

Serious adverse events occurring more than 4 weeks (28 days) after study discontinuation need only be reported if a relationship to the Novartis study drug (or therapy) is suspected.

This protocol will be monitored by the MDACC IND office.

8 ALLOWANCES FOR REMOTE PROCEDURES

When deemed appropriate by attending physician or PI, protocol-specific safety considerations, remote evaluations and interventions as described below will be allowed to ensure the safety and continued participation of research subjects, if approved by the Principal Investigator (PI). These allowances will be documented in the electronic health record and will not be considered protocol deviations.

Patient monitoring parameters will be documented in the electronic health record. Results from assessments performed at local labs will be reviewed by investigator/PI with clinically-significant lab abnormalities and/or interventions being noted. CLIA certificates for any laboratories used in the study will be collected and stored in the investigator site file. Telemedicine/phone communication with investigator and study team will be completed and documented into the electronic medical record. Physical exams, vitals, and performance status assessments may be performed by local providers, but it will not be required nor will missed timepoints cause deviations. Documentation that may be requested from local provider may include progress notes, reports of protocol related laboratory and diagnostic studies and documentation of hospitalizations. Due to the nature of telemedicine/phone visits, not all or any physical assessments will be completed.

Commercially sourced, standard-of-care agents may be administered or dispensed locally, and oral investigational agents may be released in larger supplies or shipped directly to the patient. In these cases, patient safety will be documented with labs, and AE documentation performed with investigative team review. Medication shipped by the investigational pharmacy will be trackable (e.g. FedEx or UPS) with overnight delivery when necessary.

9 PROTOCOL AMENDMENTS, OR CHANGES IN STUDY CONDUCT

Any change or addition (excluding administrative) to this protocol requires a written protocol amendment that must be reviewed by Novartis and the Study PI before implementation. Amendments significantly affecting the safety of subjects, the scope of the investigation or the scientific quality of the study require additional approval by the IRB at the study center. A copy of the written approval of the IRB must be provided to Novartis. Examples of amendments requiring such approval are:

1. increases in drug dose or duration of exposure of subjects,
2. significant changes in the study design (e.g. addition or deletion of a control group),
3. increases in the number of invasive procedures,
4. addition or deletions of a test procedure required for monitoring of safety.

These requirements for approval should in no way prevent any immediate action from being taken by the investigator or by Novartis in the interests of preserving the safety of all patients included in the trial. If an immediate change to the protocol is felt to be necessary by the investigator and is implemented for safety reasons Novartis must be notified and the IRB at the center must be informed immediately. Amendments

affecting only administrative aspects of the study do not require formal protocol amendments or IRB approval but the IRB must be kept informed of such administrative changes. Examples of administrative changes not requiring formal protocol amendments and IRB approval include:

1. changes in the staff used to monitor trials
2. minor changes in the packaging or labeling of study drug.

10 DATA MANAGEMENT

10.1 DATA COLLECTION

10.1.1 REGISTRATION

MDACC and LBJ will serve as the sites for this study. All patients enrolled in the study will be registered in the Clinical Oncology Research System (CORe) at MDACC following procedures described in Section 3.2.2.

Patient data will be collected and entered into the Prometheus Software Platform. Prometheus is a secure portal that requires users to login with validated credentials, has granular data access controls to ensure that the minimal amount of information required to complete a task is presented, handles the de-linking and de-identification of patient information to maintain patient confidentiality. Prometheus provides a multi-institute 21 CFR 11 compliant data capture portal to simplify these tasks. Standard data collection, storage procedures, and quality assurance procedures will be followed, to ensure integrity and auditability of all information entered.

Data to be entered to include baseline data, study medications by course including, drug holds and reductions, laboratory and diagnostic test results, clinic visit data, involved disease sites and response, adverse events, and off study date.

Investigators must record the information required by the protocol.

10.1.2 CONFIDENTIALITY PROCEDURES

All pathology specimens, evaluation forms, reports and other records will be identified in a manner designed to maintain patient confidentiality. All records will be kept in a secure storage area with limited access. Clinical information will not be released without the written permission of the patient or the patient's guardian, except as necessary for monitoring by regulatory authorities, or the IRB.

The investigators and all employees and co-workers involved with this study shall not disclose or use for any purpose, other than performance of the study, any data, records or other unpublished, confidential information disclosed to those individuals for the purpose of the study. No patient identifiers will be used when analyzing or reporting the results.

11 STATISTICAL METHODS

11.1 DEFINITION OF PRIMARY ENDPOINT

Progression free survival (PFS) is defined as the time from the date of randomization to progerssion or death due to any causes, whichever is earlier.

11.2 DEFINITION OF SECONDARY ENDPOINTS

- Clinical benefit rate (CBR) will be determined by combining the complete response rate, partial response rate, and stable disease rate. Response will be evaluated by repeat imaging (CT or MRI) using RECIST 1.1 at the completion of the second cycle (8 weeks + 10 days of treatment).
- Overall survival (OS) defined as the time from the date of randomization to death
- Frequency and severity of toxicities as assessed by CTCAE v.4.0
- Correlative Studies: Binary outcomes (present vs. absent) of CTNNB1 gene or other mutation in the most recent tumor biopsy using the Solid Tumor Genomic Assay will be correlated with response to Ribociclib (LEE001), everolimus (RAD001), and letrozole. Biomarkers (PTEN, mTOR, AKT, p70S6K, and ERK1/2) will be also evaluated in pre-treatment samples.
- Liquid biopsy results at baseline will be correlate with tissue mutational analysis. At 8 weeks, liquid biopsies with be correlated with response to therapy.

11.3 ANALYTIC PLAN FOR PRIMARY OBJECTIVE

We will use descriptive statistics to summarize the demographic and clinical characteristics of patients. We will estimate PFS with the Kaplan-Meier product-limit estimator. The longrank test will be used to compare the PFS between two arms.

11.4 ANALYTIC PLAN FOR SECONDARY OBJECTIVES

- We will estimate the CBR with exact 95% binomial confidence intervals.
- We will estimate OS with the Kaplan-Meier product-limit estimator.
- Toxicities will be tabulated by type and grade according to CTCAE v4.0.
- Correlative Studies: In an exploratory analysis, CTNNB1 and other gene mutation status and PTEN expression status will be correlated with response to therapy using two-tailed Fisher Exact tests. The Cox model will be used to correlate CTNNB1 and other gene mutation status and PTEN expression status with PFS. Response rates with exact 95% confidence intervals and Kaplan-Meier curves of PFS will be reported for the binary outcomes of CTNNB1 and other mutation present/absent and PTEN expression present/absent.

11.5 DEFINITION OF POPULATIONS FOR ANALYSES

Patients who are withdrawn from the study due to toxicity before being assessed for response will be counted as treatment failures.

11.6 SAMPLE SIZE JUSTIFICATION

Bayesian optimal phase 2 (BOP2) design (Zhou et al., 2017) will be used for futility monitoring. An interim analysis is planned when 18 patients are enrolled in each arm. Due to randomness of randomization, the sample size of one of the arms might be slightly larger than 18 at the interim. Let n denote the interim sample size and N denote the maximum sample size. Let PFS_exp and PFS_ctl denote the median PFS for experimental arm and control arm, respectively. We will stop the trial for futility if

$$\Pr(PFS\_exp>PFS\_ctl \mid data)<\lambda(n/N)^{\alpha},$$

where $\lambda=0.9$ and $\alpha=0.9$ are design parameters optimized to minimize the chance of incorrectly claiming that an efficacious treatment is not promising (i.e., type II error) under the alternative hypothesis $H_1: PFS\_exp=6$ months, while controlling the type I error rate at 0.05 under the null hypothesis $H_1: PFS\_exp=PFS\_ctl=3$

months (i.e., the chance of incorrectly claiming that an inefficacious treatment is promising is no more than 5%). The posterior probability will be calculated based on the inverse gamma-exponential model, assuming that the PFS follows an exponential distribution with a non-informative inverse gamma distribution IG(0.01, 0.01). Under the above design, we will accrue a minimum of 36 evaluable patients and a maximum of 70 evaluable patients.

We will enroll 99 patients to obtain 35 evaluable patients per arm, which yield 80% power to detect an improvement of the median PFS from 3 months to 6 months, hazard ratio of 0.5, with alpha =0.05. This calculation is performed using nQuery Advisor ® 7.0 (Copyright © 1995-2007, Statistical Solutions, Saugus, MA), assuming that the PFS follows an exponential distribution, 1-year accrual time, and maximum study duration of 2 years.

We will enroll at least 14 patients with *CTNNB1*. We will use Fisher's exact test (and longrank test) to compare patients with/without *CTNNB1*mutation with respect to PFS and CBR. With 14 of patients with *CTNNB1*mutation we will have 80% power with a 1-sided significance level of 0.10 to detect a difference in CBR rates of 0.33 for patients without *CTNNB1*mutation and 0.70 for patients with *CTNNB1* mutation. This power calculation was performed with nQuery Advisor ® 7.0 (Copyright © 1995-2007, Statistical Solutions, Saugus, MA).

11.7 TOXICITY MONITORING

We will monitor the rate of unacceptable toxicity independently for the two arms in Cycle 1 only. Grade 3+ non-hematologic toxicity and grade 4 hematologic toxicity will be considered unacceptable. We will use the Bayesian optimal phase 2 (BOP2) method (Zhou et al., 2017) to monitor the unacceptable toxicity rate.

Let p_{tox} denote the probability of toxicity. We will consider H_0: p_{tox}> 33%, representing that toxicity is too high. We will stop the trial early if

$$Pr(p_{tox} \leq 0.33|data) < \lambda(\frac{n}{N})^{\alpha},$$

where λ=0.75 and α=0.4 are design parameters optimized to minimize the chance of incorrectly claiming that a safe treatment is unacceptable under the alternative hypothesis $H_1: p_{tox} = 0.2$, while controlling the type I error rate at 0.2 (i.e., the chance of incorrectly claiming that an overly toxic treatment is acceptable is no more than 20%). Assuming a Beta(0.33,0.67) prior distribution for p_{tox}, the above decision rule corresponds to the following stopping boundaries:

Table 16: Optimized stopping boundaries

# patients treated	Stop if # toxicity >=
5	3
10	4
20	7
35	10

Based on Table 16, we perform the interim analysis when the number of enrolled patients reaches 5, 10 and 20. When the total number of patients reaches the maximum sample size of 35, we conclude that the treatment is acceptably safe if the number of toxicities are less than 10; otherwise we conclude that the treatment is unacceptable.

Below are the operating characteristics of the design based on 10000 simulations using the BOP2 web application, which is available at http://www.trialdesign.org.

Table 17: Operating characteristics

Toxicity rate	Early stopping (%)	Claim acceptable (%)	Sample size
0.20	17.25	76.25	30.7
0.33	60.06	19.29	20.5
0.45	90.29	1.08	11.9

The investigator is responsible for completing an efficacy/toxicity summary report and submitting it to the IND Office Medical Affairs and Safety Group for review and approval. This should be submitted after 5, 10, 20 and 35 patients per arm have completed two cycles of study therapy.

A copy of the cohort summary should be placed in the investigator's regulatory binder under "sponsor correspondence."

12 PROCEDURES AND INSTRUCTIONS

12.1 PUBLICATION OF RESULTS

Any formal presentation or publication of data from this trial may be published after review and comment by Novartis and prior to any outside submission. Novartis must receive copies of any intended communication in advance of publication (at least fifteen working days for presentational materials and abstracts and thirty working days for manuscripts). These requirements acknowledge Novartis' responsibility to provide peer input regarding the scientific content and conclusions of such publications or presentations. Principal Investigation/Institution shall have the final authority to determine the scope and content of its publications, provided such authority shall be exercised with reasonable regard for the interests of Novartis and, in accord with the trial contract and shall not permit disclosure of Novartis confidential or proprietary information.

12.2 DISCLOSURE AND CONFIDENTIALITY

The investigator agrees to keep all information provided by Novartis in strict confidence and to request similar confidentiality from his/her staff and the IRB/IEC/REB. Study documents provided by Novartis (investigators' brochures and other material) will be stored appropriately to ensure their confidentiality. The information provided by Novartis to the investigator may not be disclosed to others without direct written authorization from Novartis, except to the extent necessary to obtain informed consent from patients who wish to participate in the trial.

12.3 DISCONTINUATION OF STUDY

Novartis reserves the right to discontinue any study under the conditions specified in the clinical trial agreement.

12.4 ETHICS AND GOOD CLINICAL PRACTICE

This study must be carried out in compliance with the protocol and the principles of Good Clinical Practice, as described in Novartis standard operating procedures and:

- ICH Harmonized Tripartite Guidelines for Good Clinical Practice 1996. Directive 91/507/EEC, The Rules Governing Medicinal Products in the European Community.
- US 21 Code of Federal Regulations dealing with clinical studies (including parts 50 and 56 concerning informed consent and IRB regulations).
- Declaration of Helsinki and amendments, concerning medical research in humans (Recommendations Guiding Physicians in Biomedical Research Involving Human Subjects).
- The investigator agrees to adhere to the instructions and procedures described in it and thereby to adhere to the principles of Good Clinical Practice to which it conforms.

12.5 INSTITUTIONAL REVIEW BOARD/INDEPENDENT ETHICS COMMITTEE

Before implementing this study, the protocol, the proposed informed consent form and other information to subjects, must be reviewed by a properly constituted Institutional Review Board/Independent Ethics Committee/Research Ethics Board (IRB/IEC/REB). A signed and dated statement that the protocol and informed consent have been approved by the IRB/IEC/REB must be given to Novartis before study initiation. Any amendments to the protocol, other than administrative ones, must be reviewed by Novartis approved by this committee.

12.6 PROTOCOL REVIEW AND MONITORING

The University of Texas MD Anderson Cancer Center IND Office will monitor the study investigators to assure data recording, and protocol adherence. The site principal investigator and staff are expected to cooperate and provide all relevant study documentation in detail at each site visit on request for review. MD Anderson Cancer Center will monitor and/or audit the other participating sites to assure satisfactory protocol adherence and enrollment. The site will be visited on a regular basis by the Clinical Study Monitor, who will check completed source documentation, discuss the progress of the study and monitor drug according to good clinical practice (GCP). The monitoring will also include source data verification (SDV).

12.7 INFORMED CONSENT

Written informed consent will be obtained via in person or remote processes according to the local site's policies and standard operating procedures.

The investigator must explain to each subject (or legally authorized representative) the nature of the study, its purpose, the procedures involved, the expected duration, the potential risks and benefits involved and any discomfort it may entail. Each subject must be informed that participation in the study is voluntary and that he/she may withdraw from the study at any time and that withdrawal of consent will not affect his/her subsequent medical treatment or relationship with the treating physician.

This informed consent should be given by means of a standard written statement, written in non-technical language. The subject should read and consider the statement before signing and dating it and should be given a copy of the signed document. No patient can enter the study before his/her informed consent has been obtained.

The informed consent form is part of the protocol and must be submitted by the investigator with it for IRB/IEC/REB approval.

12.8 DECLARATION OF HELSINKI

The investigator must conduct the trial in accordance with the principles of the Declaration of Helsinki. Copies of the Declaration of Helsinki and amendments will be provided upon request or can be accessed via the website of the World Medical Association at http://www.wma.net/e/policy/17-c_e.html.

13 REFERENCES

Afinitor®/Votubia® (RAD001) Everolimus Investigator's Brochure Edition 14, Release date: 12-May-2015

Ribociclib (LEE011) Investigator's Brochure, Version 8.0, Release Date 7-September-2015

Atkins, MB, Hildalgo, M, Stadler, WM, et al (2004) Randomized phase II study of multiple dose levels of CCI-779, a novel mammalian target of rapamycin kinase inhibitor, in patients with advanced refractory renal cell carcinoma. J Clin Oncology; 22(5):909-18.

Baselga J, et al (2009) Phase II randomized study of neoadjuvant everolimus plus letrozole compared with placebo plus letrozole in patients with estrogen receptor-positive breast cancer. J Clin Oncol; 27(16):2630-7.

Bjornsti MA and Houghton PJ (2004) The TOR pathway. A target for cancer chemotherapy. Nat Rev Cancer; 4:335-338.

Boulay A, Lane HA (2007) The mammalian target of rapamycin kinase and tumor growth inhibition. Recent Results Cancer Res. 2007;172:99-124.

Bowker SL, et al (2006) Increased cancer-related mortality for patients with type 2 diabetes who use sulfonylureas or insulin: Response to Farooki and Schneider. Diabetes Care;29(8):1990-1.

Cantrell LA, et al (2010) Metformin is a potent inhibitor of endometrial cancer cell proliferation--implications for a novel treatment strategy. Gynecol Oncol;116(1):92-8.

Decensi A, et al (2010). Metformin and cancer risk in diabetic patients: a systematic review and meta-analysis. Cancer Prev Res;3(11):1451-61.

Dedes KJ, et al (2011) Emerging therapeutic targets in endometrial cancer. Nat Rev Clin Oncol;8(5):261-71.

Evans JM, et al (2005) Metformin and reduced risk of cancer in diabetic patients. BMJ;330(7503):1304-5.

Fellman, B. M. 2014. Stata command for calculating adverse event and efficacy stopping boundaries for phase II single-arm trials. *Stata Journal.* 14(2):407-417.

Fleming GF, et al (2004) Phase III trial of doxorubicin plus cisplatin with or without paclitaxel plus filgrastim in advanced endometrial carcinoma: a Gynecologic Oncology Group Study. J Clin Oncol;22(11):2159-66.

Haritunians T, Mori A, O'Kelly J, et al (2007) Antiproliferative activity of RAD001 (everolimus) as a single agent and combined with other agents in mantle cell lymphoma. Leukemia; 21(2):333-9.

Ingelman-Sundberg M (2004) Human drug metabolising cytochrome P450 enzymes: properties and polymorphisms, Naunyn Schmiedebergs Arch Pharmacol; 369(1):89-104.

Ikezoe T, Nishioka C, Bandobashi K, et al (2007) Longitudinal inhibition of PI3K/Akt/mTOR signaling by LY294002 and rapamycin induces growth arrest of adult T-cell leukemia cells. Leuk Res; 31(5):673-682.

Jiralerspong S, et al (2009) Metformin and pathologic complete responses to neoadjuvant chemotherapy in diabetic patients with breast cancer. J Clin Oncol;27(20):3297-302.

J. Jac, et al. (2008); ASCO, abstract #5113

Kim TH, et al (2010) The Synergistic Effect of Conditional Pten Loss and Oncogenic K-ras Mutation on Endometrial Cancer Development Occurs via Decreased Progesterone Receptor Action. J Oncol:139087

Krishna G, Moton A, Lei Ma L, Savant I, Martinho M, Seiberling M, McLeod J. Effects of oral posaconazole on the pharmacokinetic properties of oral and intravenous midazolam: A phase I, randomized, open-label, crossover study in healthy volunteers. Clinical Therapeutics, 2009; 31: 286-98.

Jemal A, et al (2010) Cancer statistics, 2010. CA Cancer J Clin; 60(5):277-300.

Johnston PB, Inwards DJ, et al (2010) A Phase II trial of the oral mTOR inhibitor everolimus in relapsed Hodgkin lymphoma. Am J Hematol. May;85(5):320-4

Lane H, Tanaka C, Kovarik J, et al (2003) Preclinical and clinical pharmacokinetic / pharmacodynamic (PK/PD) modeling to help define an optimal biological dose for the oral mTOR inhibitor, RAD001, in oncology [abstract]. J Clin Oncol; 22:237A.

Lane HA, Wood JM, McSheehy PM, et al (2009) mTOR inhibitor RAD001 (everolimus) has antiangiogenic/vascular properties distinct from a VEGFR tyrosine kinase inhibitor. Clin Cancer Res; 15(5):1612-1622.

Loomba R, Rowley A, Wesley R, et al (2008). Systematic review: the effect of preventive lamivudine on hepatitis B reactivation during chemotherapy. Ann Intern Med 148(7): 519-28.

Lu CH, Wyszomierski SL, Tseng LM, et al (2007) Preclinical Testing of Clinically Applicable Strategies for Overcoming Trastuzumab Resistance Caused by PTEN Deficiency. Clin Cancer Res; 13(19):5883-5888.

Ma BB, et al (2004) The activity of letrozole in patients with advanced or recurrent endometrial cancer and correlation with biological markers--a study of the National Cancer Institute of Canada Clinical Trials Group. Int J Gynecol Cancer;14(4):650-8.

Mabuchi S, Altomare DA, Cheung M, et al (2007) RAD001 inhibits human ovarian cancer cell proliferation, enhances cisplatin-induced apoptosis, and prolongs survival in an ovarian cancer model. Clin Cancer Res; 13(14):4261-4270.

Manning BD, Cantley LC (2007) AKT/PKB signaling: navigating downstream. Cell; 129(7):1261-74.

Maxwell, GL, et al (1998) Mutation of the PTEN tumor suppressor gene in endometrial hyperplasias. Cancer Res;58(12):2500-3.

Milam MR, et al (2007) Reduced progression of endometrial hyperplasia with oral mTOR inhibition in the Pten heterozygote murine model. Am J Obstet Gynecol;196(3):247 e1-5.

Motzer RJ, et al. Phase 3 trial of everolimus for metastatic renal cell carcinoma : final results and analysis of prognostic factors. Cancer;116(18):4256-65.

Mutter GL, et al (2000) Altered PTEN expression as a diagnostic marker for the earliest endometrial precancers. J Natl Cancer Inst;92(11):924-30.

O'Reilly T, McSheehy PM (2009) Biomarker Development for the Clinical Activity of the mTOR Inhibitor everolimus (RAD001): Processes, Limitations, and Further Proposals. Tran Oncol; in the press.

Oza AM, Biagi J, Chapman W, Tsao M, Kamel-Reid S, Hedley D, Hansen C, Dancey J, Eisenhauer E (2006) Molecular correlates associated with a phase II study of temsirolimus (CCI-779) in patients with metastatic or recurrent endometrial cancer--NCIC IND 160. Proceedings from Annual American Society of Clinical Oncology Meeting; 3003.

Podsypanina K, et al (2009) An inhibitor of mTOR reduces neoplasia and normalizes p70/S6 kinase activity in Pten+/- mice. Proc Natl Acad Sci U S A; 98(18):10320-5.

Pollak M (2008) Insulin and insulin-like growth factor signalling in neoplasia. Nat Rev Cancer; 8(12):915-28.

Prat, J (2004) Prognostic parameters of endometrial carcinoma. Hum Pathol; 35(6):649-62.

Risinger JI, et al (1997) PTEN/MMAC1 mutations in endometrial cancers. Cancer Res; 57(21):4736-8.

Romero IL, et al (2012) Relationship of type II diabetes and metformin use to ovarian cancer progression, survival, and chemosensitivity. Obstet Gynecol;119(1):61-7.

Rossi F, Ehlers I, Agosti V, et al (2006) Oncogenic Kit signaling and therapeutic intervention in a mouse model of gastrointestinal stromal tumor. Proc Natl Acad Sci U S A; 103(34):12843-12848.

Salvesen HB, et al (2001) PTEN methylation is associated with advanced stage and microsatellite instability in endometrial carcinoma. Int J Cancer; 91(1):22-6.

Siegel R, et al (2011) Cancer Statistics, 2011: the impact of eliminating socioeconomic and racial disparities on premature cancer deaths. CA Cancer J Clin; 61(4):212-236.

Sieghart W, Fuereder T, Schmid K, et al (2007) Mammalian target of rapamycin pathway activity in hepatocellular carcinomas of patients undergoing liver transplantation. Transplantation; 83(4):425-432.

Simon R (1989) Optimal two-stage designs for phase II clinical trials. Control Clin Trials; 10(1):1-10.

Slomovitz BM, et al (2010) A phase 2 study of the oral mammalian target of rapamycin inhibitor, everolimus, in patients with recurrent endometrial carcinoma. Cancer;116(23):5415-9.

Tabernero J, Rojo F, Casado E et al. (2005) A phase 1 study with tumor molecular pharmacodynamic evaluation of dose and schedule of the oral mTOR inhibitor RAD001 (everolimus) in patients with advanced solid tumors. [abstract]. J.Clin Oncol; 23 16S: 3007.

Tabernero J, Rojo F, Emiliano C, et al. (2008) Dose and schedule dependent inhibition of the mammalian target of rapamycin pathway with everolimus: A phase I tumor pharmacodynamic study in patients with advanced solid tumors. J. Clin Oncol; 28:10.

Tashiro H, et al (1997) Mutations in PTEN are frequent in endometrial carcinoma but rare in other common gynecological malignancies. Cancer Res; 57(18):3935-40.

Thigpen, JT et al (2004) Phase III trial of doxorubicin with or without cisplatin in advanced endometrial carcinoma: a gynecologic oncology group study. J Clin Oncol;22(19):3902-8.

Torres-Arzayus MI, Yuan J, et al (2007) Targeting the AIB1 oncogene through mammalian target of rapamycin inhibition in the mammary gland. Cancer Res; 66(23):11381-11388.

Treeck O, Wackwitz B, Haus U, et al (2006) Effects of a combined treatment with mTOR inhibitor RAD001 and tamoxifen in vitro on growth and apoptosis of human cancer cells. Gynecol Oncol; 102(2):292-299.

Tuncyurek P, Mayer JM, Klug F, et al (2007) Everolimus and mycophenolate mofetil sensitize human pancreatic cancer cells to gemcitabine in vitro: a novel adjunct to standard chemotherapy? Eur Surg Res; 39(6):380-387.

Wanner K, Hipp S, Oelsner M, et al (2006) Mammalian target of rapamycin inhibition induces cell cycle arrest in diffuse large B cell lymphoma (DLBCL) cells and sensitises DLBCL cells to rituximab. Br J Haematol; 134(5):475-484.

Wang L, Harris TE, Roth RA, et al (2007) PRAS40 regulates mTORC1 kinase activity by functioning as a direct inhibitor of substrate binding. J Biol Chem; 282(27):20036-44.

Witzig TE, Geyer SM, Ghobrial I, et al (2005) Phase II trial of single-agent temsirolimus (CCI-779) for relapsed mantle cell lymphoma. J Clin Oncol; 23(23):5347-5356.

Witzig TE, Reeder CB, et al (2011) A phase II trial of the oral mTOR inhibitor everolimus in relapsed aggressive lymphoma. Leukemia 25, 341–347

Wullschleger S, Loewith R, Hall MN (2006) TOR signaling in growth and metabolism. Cell; 124(3):471-84.

Yeo W, Lam KC, Zee B, et al. (2004) Hepatitis B reactivation in patients with hepatocellular carcinoma undergoing systemic chemotherapy. Annals of Oncology 15: 1661-1666

Zeng Z, Sarbassov dos D, Samudio IJ, et al (2007) Rapamycin derivatives reduce mTORC2 signaling and inhibit AKT activation in AML. Blood; 109(8):3509-3512.

Zhou C, et al (2003) Rapamycin inhibits telomerase activity by decreasing the hTERT mRNA level in endometrial cancer cells. Mol Cancer Ther; 2(8):789-95.

Zhou H, Lee JJ, Yuan Y. (2017) BOP2: Bayesian optimal design for phase II clinical trials with simple and complex endpoints. Stat Med; 36:3302-14.

Zitzmann K, De Toni EN, Brand S, et al (2007) The novel mTOR inhibitor RAD001 (everolimus) induces antiproliferative effects in human pancreatic neuroendocrine tumor cells. Neuroendocrinology; 85(1):54-60

Bardia A, Modi S, Oliveira M, Campone M, Ma B, Dirix L, Weise A, Nardi L, Zhang V, Bhansali SG, Hewes B, Chavez-MacGregor M. Triplet therapy with ribociclib, everolimus, and exemestane in women with HR+/HER2– advanced breast cancer. [abstract]. In: Proceedings of the Thirty-Eighth Annual CTRC-AACR San Antonio Breast Cancer Symposium: 2015 Dec 8-12; San Antonio, TX. Philadelphia (PA): AACR; Cancer Res 2016;76(4 Suppl):Abstract nr P6-13-01

APPENDIX A – GOG PERFORMANCE STATUS

GOG Score	Karnofsky Score	Activity Level
0	90-100	Fully active, unrestricted activities of daily living
1	70-80	Ambulatory, but restricted in strenuous activity
2	50-60	Ambulatory, and capable of self care. Unable to work. Out of bed for >50% of waking hours
3	30-40	Limited self care, or confined to bed or chair 50% of waking hours. Needs special assistance
4	10-20	Completely disabled, and no self care
5	0	Dead

APPENDIX B - LIST OF PROHIBITED MEDICATIONS AND PERMITTED MEDICATIONS TO BE USED WITH CAUTION DURING EVEROLIMUS + LETROZOLE + RIBOCICLIB

List of Prohibited Medications during Everolimus + Letrozole + Ribociclib investigational treatment

Category	Drug Name
Strong CYP3A4/5 Inhibitors	Atazanavir/ritonavir, boceprevir, clarithromycin, cobicistat, conivaptan, danoprevir/ritonavir, eltegravir/ritonavir, grapefruit juice, indinavir/ritonavir, itraconazole, ketoconazole, lopinavir/ritonavir, mibefradil, nefazodone, nelfinavir, ombitasvir/paritaprevir/dasabuvir/ritonavir (VIEKIRA PAK), posaconazole, ritonavir, saquinavir/ritonavir, telaprevir, telithromycin, tipranavir/ritonavir, troleandomycin, voriconazole
Strong CYP3A4/5 Inducers	Carbamazepine[3], enzalutamide, lumacaftor, mitotane, phenobarbital, phenytoin[3], rifabutin, rifampin (rifampicin)[3], St. John's wort (hypericum perforatum)[2,3]
CYP3A4/5 substrates with NTI[1]	Alfentanil, astemizole, cisapride, cyclosporine, diergotamine (dihydroergotamine), ergotamine, fentanyl, lomitapide[5], lovastatin, pimozide, quinidine, sirolimus, tacrolimus
Medications that carry a risk for QT prolongation[4]	Amiodarone, anagrelide, arsenic trioxide, astemizole, azithromycin, bepridil, chloroquine, cocaine chlorpromazine, cilostazol, ciprofloxacin, cisapride, clarithromycin, disopyramide, dofetilide, domperidone, donepezil, dronedarone, droperidol, erythromycin, escitalopram, flecainide, fluconazole, gatifloxacin, grepafloxacin, halofantrine, haloperidol, ibutilide, levofloxacin, levomepromazine, levosulpiride, levomethadyl, mesoridazine methadone, moxifloxacin, ondansetron, oxaliplatin, papaverine HCl (intra-coronary), pentamidine, pimozide, probucol, procainamide, propofol, quinidine, roxithromycin, sevoflurane, sotalol, sulpiride, sultopride, terlipressin, terodiline, terfenadine, thioridazine, vandetanib
Other investigational and antineoplastic therapies	Other investigational therapies must not be used while the patient is on the study. Anti-cancer therapy (chemotherapy, hormonal therapy, including but not limited to all SERMS [including raloxifene], biologic or radiation therapy [except for palliative radiotherapy], and surgery) other than the study treatments must not be given while the patient is on the study medication. If such agents are required, then the patient must discontinue the study drug.

Herbal preparations/medications	Herbal preparations/medications known as strong inducers or inhibitors of CYP3A4/5 or those with a known risk of QT prolongation are prohibited throughout the study. These herbal medications include, but are not limited to: St. John's wort, Kava, ephedra (ma huang), gingko biloba, dehydroepiandrosterone (DHEA), yohimbe, saw palmetto, black cohosh, and ginseng. Patients should stop using these herbal medications 7 days prior to first dose of study drug.

[1] NTI = narrow therapeutic index drugs whose exposure-response indicates that increases in their exposure levels by the concomitant use of potent inhibitors may lead to serious safety concerns (e.g., Torsades de Pointes) or drugs which have < 2-fold difference in the minimum toxic concentrations and minimum effective concentrations in the blood.
[2] Herbal product
[3] P-gp inducer

[4] The list provided is as of January 2018. Check https www crediblemeds.org/healthcare-providers/drug-list for the most updated list.

[5]Drug has warning for risk of hepatotoxicity.As far as possible, avoid co-administration of QT prolonging drugs or any other drugs with the potential to increase the risk of drug-related QT prolongation (e.g., via a potential DDI that increases the exposure of ribociclib or the exposure of the QT prolonging drug). A definitive list of drugs with a known risk, possible risk, or conditional risk of QT prolongation and/or Torsades de Pointes (TdP) is available online at qtdrugs.org.

Source: Novartis PK Sciences Memorandum: Drug-Drug Interactions (DDI) and Co-medication Considerations for Novartis Clinical Trials (January 2018), which is compiled from Indiana University "Clinically Relevant" Flockhart Table

™, University of Washington Drug Interaction Database, and FDA Drug Development and Drug Interactions: Table of Substrates, Inhibitors and Inducers.

List of Medications to be used with caution during Everolimus + Letrozole + Ribociclib investigational treatment

Category	Drug Name
Moderate CYP3A4/5 inhibitors	Aprepitant, amprenavir, asafoetida resin (Ferula asafoetida) cimetidine, crizotinib, diltiazem, faldaprevir, imatinib, isavuconazole, netupitant, nilotinib, tofisopam, Schisandra sphenanthera (nan wu wei zi), verapamil
Moderate CYP3A4/5 inducers	Bosentan, dabrafenib, efavirenz, etravirine, genistein,lopinavir5, modafinil, nafcillin, telotristat

Sensitive CYP3A4/5 Substrates[1]	Alpha-dihydroergocryptine, apixaban, aprepitant, atorvastatin, avanafil, bosutinib, brotizolam, budesonide, buspirone, cobimetinib, darifenacin, dasatininb, ebastine, eletriptan, eplerenone, everolimus, felodipine, fluticasone, grazoprevir, ibrutnib, isavuconazole, ivabradine, ivacaftor, lumefantrine, lurasidone, maraviroc, midazolam, midostaurin, naloxegol, neratinib, perospirone, quetiapine, ridaforolimus, sildenafil, simeprevir, ticagrelor, tilidine, tolvaptan, triazolam, ulipristal, vardenafil, venetoclax, vicriviroc, voclosporin
BSEP inhbitors	Alectinib, atorvastatin, bromocriptine, candesartan, clobetasol, clofaziminie, dabigatran, dipyridamole, glyburide, grazoprevir, ledipasvir, mifepristone, pioglitazone, reserpine, rifamycin, simeprevir, telmisartan, timcodar, troglitazone, valinomycin, velpatasvir
MATE1/2 substrates	Acyclovir, cephalexin, cimetidine, fexofenadine, ganciclovir, glycopyrronium, metformin, pindolol, plisicainide, ranitidine, topotecan, varenicline
OCT1/2 substrates[2]	Amantadine, 6-beta-hydroxycortisol, carboplatin, cephalexin, cephradine, cisplatin, ipratropium, lamivudine, lingagliptin, metformin, oxybutynin, oxyplatin, phenformin, picoplatin, pilsicainide, pindolol, ranitidine, sorafenib, tropisetron, trospium, umeclidinium, zidovudine
BCRP substrates	Daunorubicin, dolutegravir, doxorubicin, ethinyl estradiol, hematoporphyrin, imatinib, irinotecan, methotrexate, mitoxantrone, pitavastatin, rosuvastatin, sulfasalazine, sofosbuvir, tenofovir, topotecan, venetoclax

Medications that carry a possible risk for QT prolongation	Alfuzosin, apomorphine, aripiprazole, artenimol+piperaquine, asenapine, atazanavir, atomoxetine, bedaquiline, bendamustine, bortezomib, bosutinib, buprenorphine, cabozantinib, capecitabine, certinib, clomipramine, crizotinib, clozapine, cyamemazine (cyamepromazine), debrafenib, dasatinib, degarilix, delamanid, desipramine, dexmedetomidine, dolasetron, efavirenz, eliglustat, epirubicin, eribulin mesylate, ezogabine (retigabine), famotidine, felbamate, fingolimod, flupentixol, gemifloxacin, granisetron, hydrocodone-ER, iloperidone, imipramine (melipramine), isradipine, ketanserin, lapatinib, lenvatinib, leuprolide, lithium, loperamide, melperone, midostaurin, mifepristone, mirabegron, mirtazapine, moexipril/HCTZ, necitumumab, nilotinib, norfloxacin, nortiptyline, nusinersen, ofloxacin, olanzapine, osimertinib, oxytocin, paliperidone, palonosetron, panabinostat, pasireotide, pazopanib, perflutren lipid microspheres, perphenazine, pilsicainide, pimavanserin, pipamperone, promethazine, prothipendyl, quetiapine, ranolazine rilpivirine, risperidone, romidepsin, sertindole, sorafenib, sunitinib, tamoxifen, telavancin, tetrabenzine, tipiracil/trifluridine, tizanidine, tolterodine, toremifene, trimipramine, tropisetron, vardenafil, vemurafenib, venlafaxine, vorinostat, ziprasidone

[1] Sensitive substrates: Drugs whose plasma AUC values have been shown to increase 5-fold or higher when coadministered with a potent inhibitor.

[2] The list provided is as of January 2018. Check https www crediblemeds.org/healthcare-providers/drug-list for the most updated list.

[3] MATE1 and MATE2 share considerable substrate specificity.

4 OCT1 and OCT2 share considerable substrate specificity.

5 Lopinavir is prohibited when combined with ritonavir (see Table 14-1)

Source: Novartis PK Sciences Memorandum: Drug-Drug Interactions (DDI) and Co-medication Considerations for Novartis Clinical Trials (January 2018), which is compiled from Indiana University "Clinically Relevant" Flockhart Table™, University of Washington Drug Interaction Database, and FDA Drug Development and Drug Interactions: Table of Substrates, Inhibitors and Inducers.
