# Supplementary material for: Efficacy of everolimus and letrozole with or without ribociclib in recurrent endometrial cancer: a phase 2 randomized clinical trial
Source: Nat Commun. 2026 Sep 1;17:10413. doi: 10.1038/s41467-026-77049-3 (PMC13631232; doi:10.1038/s41467-026-77049-3)
Supplement: Supplementary file 2 [file 41467_2026_77049_MOESM2_ESM.pdf]

nature portfolio

Corresponding author(s): Pamela T. Soliman

Last updated by author(s): Jun 21, 2026

Reporting Summary

Nature Portfolio wishes to improve the reproducibility of the work that we publish. This form provides structure for consistency and transparency in reporting. For further information on Nature Portfolio policies, see our Editorial Policies and the Editorial Policy Checklist.

Statistics

For all statistical analyses, confirm that the following items are present in the figure legend, table legend, main text, or Methods section.

n/a	Confirmed	
☐	☒	The exact sample size (n) for each experimental group/condition, given as a discrete number and unit of measurement
☐	☒	A statement on whether measurements were taken from distinct samples or whether the same sample was measured repeatedly
☐	☒	The statistical test(s) used AND whether they are one- or two-sided<br>*Only common tests should be described solely by name; describe more complex techniques in the Methods section.*
☐	☒	A description of all covariates tested
☐	☒	A description of any assumptions or corrections, such as tests of normality and adjustment for multiple comparisons
☐	☒	A full description of the statistical parameters including central tendency (e.g. means) or other basic estimates (e.g. regression coefficient) AND variation (e.g. standard deviation) or associated estimates of uncertainty (e.g. confidence intervals)
☐	☒	For null hypothesis testing, the test statistic (e.g. F, t, r) with confidence intervals, effect sizes, degrees of freedom and P value noted<br>*Give P values as exact values whenever suitable.*
☐	☒	For Bayesian analysis, information on the choice of priors and Markov chain Monte Carlo settings
☒	☐	For hierarchical and complex designs, identification of the appropriate level for tests and full reporting of outcomes
☒	☐	Estimates of effect sizes (e.g. Cohen's d, Pearson's r), indicating how they were calculated

Our web collection on statistics for biologists contains articles on many of the points above.

Software and code

Policy information about availability of computer code

Data collection	Clinical data collection was supported in part by The University of Texas MD Anderson Cancer Center's Prometheus informatics system
Data analysis	Analyses were performed using STATA statistical software version 16.1. Figures were created using STATA statistical software version 16.1 and R version 4.5.1

For manuscripts utilizing custom algorithms or software that are central to the research but not yet described in published literature, software must be made available to editors and reviewers. We strongly encourage code deposition in a community repository (e.g. GitHub). See the Nature Portfolio guidelines for submitting code & software for further information.

Data

Policy information about availability of data

All manuscripts must include a data availability statement. This statement should provide the following information, where applicable:

- Accession codes, unique identifiers, or web links for publicly available datasets
- A description of any restrictions on data availability
- For clinical datasets or third party data, please ensure that the statement adheres to our policy

The study protocol (with the statistical analysis plan) is available in the Supplementary Information file. Source data are provided with this paper. Requests for additional deidentified clinical data not included in the source data file should be directed to the corresponding author and will be evaluated on a case-by-case basis. Reason for access restrictions is our Institutional Review Board (IRB) policy. Requests should be made to the corresponding author beginning 3 months and ending 5 years after the publication of the article. The expected timeframe for response to access requests is within 30 business days. Once access has been

granted, the data will be available without restriction for general research use and indefinitely.

Research involving human participants, their data, or biological material

Policy information about studies with human participants or human data. See also policy information about sex, gender (identity/presentation), and sexual orientation and race, ethnicity and racism.

Reporting on sex and gender	All patients enrolled into the study were female. Women of all races or ethnicity were eligible for enrollment on this trial.
Reporting on race, ethnicity, or other socially relevant groupings	Details on race and ethnicity of all participants are reported in Table 1. Ethnicity and race were determined based on self-report.
Population characteristics	Table 1 summarizes the population characteristics.<br>Key trial eligibility criteria included ≥18 years old, histologically-confirmed endometrioid or mixed endometrioid EC, advanced/recurrent disease not amenable to curative therapy, Eastern Cooperative Oncology Group Performance status (ECOG PS) 0-1, measurable disease per RECIST v1.1 criteria, and no more than two prior chemotherapeutic regimens in the recurrent setting. Prior exposure to non-chemotherapy systemic regimens (e.g. endocrine therapies, immunotherapy, targeted therapy, antibody-drug conjugates) was permissible and not counted in the maximum number of prior lines. Prior treatment with letrozole was permitted but those with prior exposure to mTOR inhibitors were excluded. Estrogen/progesterone receptor (ER/PR) positivity was not required. Patients with uterine sarcomas, carcinosarcomas, and pure serous or clear cell carcinomas were excluded. Prespecified-protocol enrollment of ≥14 patients with a CTNNB1 mutation was required.
Recruitment	This investigator initiated study was open at two institutions (The University of Texas MD Anderson Cancer Center and Lyndon B. Johnson General Hospital). Patients were eligible for the study if they fulfilled the eligibility criteria of the study. Members of all races and ethnic groups were eligible and there was no bias in recruitment.
Ethics oversight	This investigator-initiated study (ClinicalTrials.gov Identifier: NCT03008408) was approved by the The University of Texas MD Anderson Cancer Center and Lyndon B. Johnson General Hospital institutional review boards and adhered to principles of the Declaration of Helsinki and the International Conference on Harmonization Good Clinical Practice guidelines. All patients provided written informed consent prior to enrollment in the study.

Note that full information on the approval of the study protocol must also be provided in the manuscript.

Field-specific reporting

Please select the one below that is the best fit for your research. If you are not sure, read the appropriate sections before making your selection.

[x] Life sciences [ ] Behavioural & social sciences [ ] Ecological, evolutionary & environmental sciences

For a reference copy of the document with all sections, see nature.com/documents/nr-reporting-summary-flat.pdf

Life sciences study design

All studies must disclose on these points even when the disclosure is negative.

Sample size	Safety lead-in phase: Enrollment into the safety lead-in occurred at six patient increments (up to 24 patients) starting at the starting dose level 0. Details and outcomes on the safety lead-in phase is shown in Figure 1 and the Supplemental information.<br><br>Randomized dose expansion phase: A sample size of 35 evaluable patients per arm would provide 80% power to detect an improvement in median PFS from three to six months (HR of 0.5, with alpha level of 0.05). To evaluate the effect of CTNNB1 mutations on treatment response, we prespecified enrollment ≥14 patients with a CTNNB1 mutation in the experimental arm. We used Fisher's exact test (or log-rank test if appropriate) to compare patients with or without CTNNB1 mutation with respect to CBR, ORR, PFS, and OS. With 14 of patients with CTNNB1 mutation in the experimental arm, there was 80% power with a one-sided significance level of 0.10 to detect a difference in CBR rates of 0.33 for patients without CTNNB1 mutation and 0.70 for patients with CTNNB1 mutation.
Data exclusions	All patients who received study drug were evaluable for safety and/or efficacy reporting and were not excluded.
Replication	There were no attempts for replication given the nature of the study.
Randomization	Once the was recommended phase II dose was determined in the safety lead-in phase, eligible patients were randomly assigned 1:1 in the dose expansion phase, via the OnCore clinical trial management system (Advarra,Inc, Columbia, Maryland) to either the experimental (daily oral everolimus 2.5 mg, letrozole 2.5 mg, and ribociclib 200 mg in 28-day cycles) or the control arm (daily oral everolimus 10 mg and letrozole 2.5 mg in 28-day cycles). All patients commenced treatment within 7 days following randomization.
Blinding	This was an open-label study and there was no blinding.

Reporting for specific materials, systems and methods

We require information from authors about some types of materials, experimental systems and methods used in many studies. Here, indicate whether each material, system or method listed is relevant to your study. If you are not sure if a list item applies to your research, read the appropriate section before selecting a response.

Materials & experimental systems

n/a	Involved in the study
☒	☐ Antibodies
☒	☐ Eukaryotic cell lines
☒	☐ Palaeontology and archaeology
☒	☐ Animals and other organisms
☐	☒ Clinical data
☒	☐ Dual use research of concern
☒	☐ Plants

Methods

n/a	Involved in the study
☒	☐ ChIP-seq
☒	☐ Flow cytometry
☒	☐ MRI-based neuroimaging

Clinical data

Policy information about clinical studies
All manuscripts should comply with the ICMJE guidelines for publication of clinical research and a completed CONSORT checklist must be included with all submissions.

Clinical trial registration	ClinicalTrials.gov Identifier: NCT03008408
Study protocol	The protocol is included as a supplement file in the submission.
Data collection	This investigator initiated study was open at two institutions (The University of Texas MD Anderson Cancer Center and Lyndon B. Johnson General Hospital). Patient enrollment occurred from 9-28-2017 to 1-22-2024. Data collection/cutoff was 6-14-2025.
Outcomes	The primary endpoint was PFS (time from treatment allocation to disease progression [RECIST v1.1] or death). Patients were censored at the last visit evaluating for disease status if they did not have disease progression at the time of data cutoff. If they had discontinued treatment prior to disease progression due to other reasons (e.g. toxicity or withdrawal of consent), PFS was evaluated until disease progression and censored at the start date of a new anticancer therapy if it was initiated prior to disease progression. Secondary endpoints included overall survival (OS), objective response rate (ORR), clinical benefit rate (CBR), toxicity, and presence of a somatic CTNNB1 mutation and association with treatment response. OS was defined as time from treatment allocation to death (censored at the last known date alive). CBR was defined as having prolonged stable disease (SD; ≥16 weeks) or partial (PR) or complete (CR) response by RECIST v1.1. Exploratory endpoints included correlation between efficacy endpoints and other somatic mutations. Post-hoc analyses included correlation between efficacy endpoints and other molecular features (e.g. p53, MMR, and ER/PR) or clinicopathologic characteristics. All analyses were preplanned, unless specified. Efficacy and safety analyses were performed on all patients who received at least one dose of study drug.

Plants

Seed stocks	*Report on the source of all seed stocks or other plant material used. If applicable, state the seed stock centre and catalogue number. If plant specimens were collected from the field, describe the collection location, date and sampling procedures.*
Novel plant genotypes	*Describe the methods by which all novel plant genotypes were produced. This includes those generated by transgenic approaches, gene editing, chemical/radiation-based mutagenesis and hybridization. For transgenic lines, describe the transformation method, the number of independent lines analyzed and the generation upon which experiments were performed. For gene-edited lines, describe the editor used, the endogenous sequence targeted for editing, the targeting guide RNA sequence (if applicable) and how the editor was applied.*
Authentication	*Describe any authentication procedures for each seed stock used or novel genotype generated. Describe any experiments used to assess the effect of a mutation and, where applicable, how potential secondary effects (e.g. second site T-DNA insertions, mosiacism, off-target gene editing) were examined.*
