# Supplementary material for: Efficacy of everolimus and letrozole with or without ribociclib in recurrent endometrial cancer: a phase 2 randomized clinical trial
Source: Nat Commun. 2026 Sep 1;17:10413. doi: 10.1038/s41467-026-77049-3 (PMC13631232; doi:10.1038/s41467-026-77049-3)
Supplement: Supplementary file 3 [file 41467_2026_77049_MOESM3_ESM.pdf]

nature portfolio

Peer Review File

Efficacy of everolimus and letrozole with or without ribociclib in recurrent endometrial cancer: A Phase 2 randomized clinical trial

Corresponding Author: Dr Pamela Soliman

This file contains all reviewer reports in order by version, followed by all author rebuttals in order by version.

Version 0:

Reviewer comments:

Reviewer #1

(Remarks to the Author)
How and colleagues report a randomized phase 2 clinical trial of everolimus and letrozole with or without ribociclib in recurrent endometrial cancer. The study is well written and is a valuable contribution to the literature of hormonal therapies for endometrial cancer. Key strengths include its randomized design, incorporation of somatic tumor mutation testing and molecular profiling, allowance of prior endocrine therapy, and lack of a hormone-receptor–positivity requirement for eligibility.
However, I have several concerns regarding interpretation and presentation of data, especially data related to the CTNNB1mutated cohort as it pertains to the statistical plan described in the study protocol.

MAJOR CONCERNS
1. In the statistical plan described in the manuscript (lines 183-185) and also in the Section 11.6 of the study protocol, it is stated that "With 14 patients with CTNNB1mutation we will have 80% power with a 1-sided significance level of 0.10 to detect a difference in CBR rates of 0.33 for patients without CTNNB1mutation and 0.70 for patients with CTNNB1 mutation." However, although this power calculation was based on the clinical benefit rate (CBR), the CBR in patients with CTNNB1mutation is not reported anywhere in the main manuscript. This information is included in Supplement Table 9 where, among CTNNB1 mutated tumors, CBR in the control arm was actually nominally higher than the experimental arm (93.3% in the control vs 82.4% in the experimental arm). This is a key finding that directly contradicts the primary hypothesis that ribociclib enhances the therapeutic activity of letrozole/everolimus in CTNNB1-mutated tumors. Because the power calculation for determining the required number of CTNNB1-mutated cases was based on CBR, the CBR for CTNNB1-mutated tumors in both the control and experimental arms must be reported in the abstract, main results section and explicitly acknowledged and discussed in the Discussion.
2. In the section "Impact of CTNNB1 mutation status on efficacy – a prespecified analysis", the authors describe differences in PFS and OS between CTNNB1mut and CTNNB1wt tumors within the experimental and within the control arms. Although these results are interesting, they do not address the main hypothesis stated by the authors that "addition of ribociclib would improve therapeutic efficacy, particularly among patients with CTNNB1 mutant tumors". To address that, the comparison in PFS and OS should occur in CTNNB1mut and CTNNB1wt tumors between arms (as they did with ORR in Lines 234-235), and not within arms. In other words, they should compare PFS and OS between letrozole/ribociclib vs letrozole/ribociclib/everolimus among CTNNB1mut tumors and show these PFS and OS survival curves as Figures in the main results. Based on the supplement Table 6 where these comparisons are made, there is no statistically significant difference in PFS and OS between control and experimental arms among CTNNB1 mutated tumors. These findings need to be reported in the main results and discussed in the discussion section.
3. Based on points 1 and 2 above, the statement in the abstract conclusions Lines 59-61 that "Although it did not improve survival in all-comers, the addition of ribociclib to everolimus/letrozole may specifically improve survival in CTNNB1mut EC and improve efficacy in patients previously treated with endocrine therapy" is not supported by the data (CBR, PFS and OS comparisons between arms within CTNNB1mut tumors) presented in the study. This statement in the abstract and relevant discussion need to be revised accordingly.

MINOR CONCERNS
1. Abstract, Lines 52-53, "Addition of ribociclib resulted in a higher ORR among those with prior endocrine therapy (61.5% vs

0.0%, p<0.001)": The 0% response rate of letrozole/everolimus among patients who had received prior hormonal therapy is striking and should be discussed further. What hormonal therapies did patients on letrozole/everolimus alone arm receive? In the previous study of everolimus/letrozole/metformin by the same group which allowed prior hormonal therapy and ORR was 28%, were responses seen only among hormonal therapy naive patients? What about the previous letrozole/everolimus study by Slomovitz et al.?
2. Abstract, Lines 45-46, "Secondary endpoints included toxicity, OS, ORR": Per study protocol, Section 2.3, clinical benefit was also a secondary endpoint and therefore should be included in the abstract and reported in the results. ORR is not specifically stated as a secondary endpoint in the protocol except only as part of "to determine the presence of a CTNNB1 mutation is associated with response to ribociclib (LEE011), everolimus, and letrozole."
3. Methods, Line 104, "Prespecified-protocol enrollment of ≥14 patients with a CTNNB1 mutation was required": It needs to be clarified whether ≥ 14 patients were required for each arm versus the entire study. Also, more information needs to be provided on how the investigators kept track of when the required number of CTNNB1mut tumors was achieved.
4. Discussion, Lines 291-294: In the discussion of relevant literature on CTNNB1mut tumors, the authors should also include data from a recently published study of letrozole/abemaciclib/metformin in Nature Communications which showed that presence of CTNNB1 mutations correlated with clinical benefit and that 2 of the 3 patients with complete responses to letrozole/abemaciclib/metformin harbored CTNNB1 mutations (PMID: 41339350).

Reviewer #2

(Remarks to the Author)
Randomized Phase II of double (everolimus + letrozole) or triple (+ ribociclib) association in recurrent endometrial carcinoma.
2 steps design with a safety lead-in and an open randomized ph 2 of double (everolimus + letrozole) or triple (+ ribociclib) association in recurrent endometrial carcinoma.
The objectives : To characterize the toxicity, the efficacy, and the molecular subtypes that would benefit the most from the triple combination of AI + mTOR inhibitor (everolimus) + CDK4 6 inhibitor (everolimus).
This is an important question in a population with an unmet medical need.

Major remarks :
Randomized phase 2 study conducted in only two centers, which required 5 years to complete enrollment. This is a long period for a common indication (all endometrioid/mixed EC after at least one line of chemotherapy, up to two lines).
Why was the study not expanded to include additional centers?

Population
Population is heterogenous : number of prior lines, prior hormonal treatments, histology, hormonal status can impact the response and toxicity ; 1 or 2 stratification factor could have been introduced ; to be comment in the statistical paragraph and in the discussion in particular in the safety results.
Population, in particular molecular status, are imbalanced between the safety run and the ph 2, and could impact results
The rational to include patients whatever the hormonal receptor status for an hormonal treatment association have to be explain.
The definition of ER/PR positivity, the absence of centralisation of the results could impatc the results and should also be mentioned.

In the inclusion criteria, non chemotherapy treatments are not counted as prior lines. Why are ADCs (which are chemotherapy agents) excluded from the count of prior chemotherapy lines? It could impact results on toxicity and efficacy.

Given the hypothesis of increased efficacy, particularly in the population of CTNNB1 mutated tumors considered from the outset of the study, why was this factor not used for stratification ?

Statistics
The authors mention PFS results around 6 months with the everolimus–letrozole combination. Why does the statistical hypothesis start from an estimated 3 month median PFS for the control arm? This may affect the statistical calculation of the required sample size and potentially overestimate the benefit of the investigational arm."

Results

Safety lead-in part :
Level 0 is based on safety of assocations used in BC. The rational of escalation/desescalation is unclear : the dose -1A level with higer doses of everolimus than the level 0 and level -1 is unclear ; to be explain
The results of this part of the study are not presented /commented in the results section.
It is not clearly stated which dose was selected for the phase 2 study, nor the rationale for this choice

The combination is toxic, with 20% of patients discontinuing ribociclib and 10% discontinuing the tritherapy. The authors should acknowledge this is a limitation of the study. Is that comparable as described is other trials in breast carcinoma ? To be comment/discussed.
Do the authors plan to adjust the doses if they were abble to conduct another study?

Minor remarks

What were the dose reduction rules in cases of neutropenia?

A recent biopsy or a biopsy performed at inclusion was used for non centralized molecular analysis, which may impact the results of the subgroup analysis.

Define the positivity of HR
Presentation of results : some informations in table 1 are less important than thoses in supplement datas. The results on race and social status may be mentioned in the supplements, whereas the information on previous treatments, HR status and molecular status (p53, MMR, PI3KA, PTEN, POLE) could be included in this table.

Reviewer #3

(Remarks to the Author)
I co-reviewed this manuscript with one of the reviewers who provided the listed reports. This is part of the Nature Communications initiative to facilitate training in peer review and to provide appropriate recognition for Early Career Researchers who co-review manuscripts.

Reviewer #4

(Remarks to the Author)
For the last part of the results (lines 245-249) - given the limited sample size, I think the conclusion that the there is a difference in the experimental arm but not control over-states the results. The hazard ratio in the control arm is 0.40 for PFS and 0.44 for OS in the control arm - while not as large as the experimental arm, the HRs are quite far from null. The sample size means the CI has limited precision but I think all we can conclude here is limited precision - I wouldn't say a signal in one but not the other (or rather a signal in one group, but less precision and potentially a less extreme signal in the other).

In general, we can't just compare p-values in subgroups and conclude difference if there is a significant p-value in one subgroup but not the other. The formal evaluation of this needs an interaction test. It may be the case there is a significant interaction here... but with this N and the data presented, I think it is hard to conclude there really is no signal in the control group.

Discussion line 279 - median not reached with limited follow-up, decreasing hazards of death over time, and small numbers is less meaningful. Reporting the absolute difference is OS estimates at 2 or 3 years would be more meaningful.

Minor, line 281 - this is just a report for one patient? So not a median per se but really just the PFS length of that one patient.

Version 1:

Reviewer comments:

Reviewer #1

(Remarks to the Author)
The authors have adequately addressed my previous comments. My only remaining concern regarding the revised manuscript pertains to the final statement in the abstract conclusions: “Future study designs investigating the role of ribociclib should be focused in the CTNNB1mut EC tumor population.” This conclusion appears overly strong and should, in my view, be removed.
Although cases with CTNNB1-mutant tumors were observed to demonstrate exceptional responses, this does not establish that only patients with CTNNB1-mutant tumors responded to ribociclib. Moreover, while CTNNB1-mutated tumors in the experimental arm (letrozole/everolimus/ribociclib) performed significantly better than CTNNB1 wild-type tumors, this does not necessarily indicate that the observed effect was attributable specifically to ribociclib. Prior studies have shown responses to everolimus in CTNNB1-mutated tumors, suggesting that the exceptional outcomes in this subgroup may be related to the combination of everolimus and ribociclib, rather than to ribociclib alone.

Reviewer #2

(Remarks to the Author)
Tha authors respond to questions and remarks

Reviewer #3

(Remarks to the Author)
I co-reviewed this manuscript with one of the reviewers who provided the listed reports. This is part of the Nature Communications initiative to facilitate training in peer review and to provide appropriate recognition for Early Career Researchers who co-review manuscripts.

Reviewer #4

(Remarks to the Author)
Lines 256-259: It does not appear that these p-values account for censoring. Kaplan Meier estimates should be reported with Z-tests using Kaplan-Meier variances/standard deviations.

Line 260 references results in the control arm comparing CTNNB1 mut vs wt, but I don't see an equivalent of 3c and 3d for the control arm.

Relatedly, Sup figure 3e and 3f show much less of a gap between curves (with limited n) then observed in Sup figure 3c and 3d. It is important to understand how much of the results in Sup figure 3c and 3d are due to the prognostic association of CTNNB1. Additional curves with the control arm would be important.

Lines 314-316 over-state the conclusions. Finding significant p-values in one group and non-signficant in another group do not by themselves do not "support to the clinical benefit for ribociclib in CTNNB1mut tumors" (line 309-310).

Figure 4 needs to include all patients (those without tumor measurements should be annotated in some way as no responding). It does not appear to include all patients.

Version 2:

Reviewer comments:

Reviewer #4

(Remarks to the Author)
Given the sample size, those interaction p-values are rather compelling. It could be useful to provide them somewhere in the manuscript as a reference for any future research, but this is just a thought and not a requirement.

Open Access This Peer Review File is licensed under a Creative Commons Attribution 4.0 International License, which permits use, sharing, adaptation, distribution and reproduction in any medium or format, as long as you give appropriate credit to the original author(s) and the source, provide a link to the Creative Commons license, and indicate if changes were made.
In cases where reviewers are anonymous, credit should be given to 'Anonymous Referee' and the source.
The images or other third party material in this Peer Review File are included in the article’s Creative Commons license, unless indicated otherwise in a credit line to the material. If material is not included in the article’s Creative Commons

license and your intended use is not permitted by statutory regulation or exceeds the permitted use, you will need to obtain permission directly from the copyright holder.
To view a copy of this license, visit https://creativecommons.org/licenses/by/4.0/

Pamela T. Soliman, M.D., M.P.H.
Professor and Ad Interim Chair
psoliman@mdanderson.org

Department of Gynecologic Oncology and Reproductive Medicine
1155 Pressler Street, Unit 1362
Houston, Texas 77030

Point-by-point responses to reviewers' comments (Responses in blue)

Reviewer #1 (Remarks to the Author):

How and colleagues report a randomized phase 2 clinical trial of everolimus and letrozole with or without ribociclib in recurrent endometrial cancer. The study is well written and is a valuable contribution to the literature of hormonal therapies for endometrial cancer. Key strengths include its randomized design, incorporation of somatic tumor mutation testing and molecular profiling, allowance of prior endocrine therapy, and lack of a hormone-receptor–positivity requirement for eligibility.
However, I have several concerns regarding interpretation and presentation of data, especially data related to the CTNNB1mutated cohort as it pertains to the statistical plan described in the study protocol.

MAJOR CONCERNS
1. In the statistical plan described in the manuscript (lines 183-185) and also in the Section 11.6 of the study protocol, it is stated that "With 14 patients with CTNNB1mutation we will have 80% power with a 1-sided significance level of 0.10 to detect a difference in CBR rates of 0.33 for patients without CTNNB1mutation and 0.70 for patients with CTNNB1 mutation." However, although this power calculation was based on the clinical benefit rate (CBR), the CBR in patients with CTNNB1mutation is not reported anywhere in the main manuscript. This information is included in Supplement Table 9 where, among CTNNB1 mutated tumors, CBR in the control arm was actually nominally higher than the experimental arm (93.3% in the control vs 82.4% in the experimental arm). This is a key finding that directly contradicts the primary hypothesis that ribociclib enhances the therapeutic activity of letrozole/everolimus in CTNNB1-mutated tumors. Because the power calculation for determining the required number of CTNNB1-mutated cases was based on CBR, the CBR for CTNNB1-mutated tumors in both the control and experimental arms must be reported in the abstract, main results section and explicitly acknowledged and discussed in the Discussion.

Response: Thank you for this comment. We wanted to clarify that the sample size calculation regarding *CTNNB1* mutant tumors was a prespecified subset analysis with respect to patients within the experimental arm to determine whether the addition of ribociclib would improve treatment efficacy in *CTNNB1* mutant tumors compared to those with *CTNNB1* wildtype tumors. The sample size calculation was not powered to determine whether the experimental arm was superior to the control arm among the *CTNNB1* mutant tumor population. We have clarified this in the Methods section and discussed this limitation in the Discussion section. Future studies should be powered and focus on the experimental and control regimens in a larger *CTNNB1* mutant EC tumor population.

We have added the CBR rate between the experimental and control arm among *CTNNB1* mutant tumors in the Abstract and Results section.

Please see the revisions in the following sections:

<u>Abstract</u>
Lines 46-48
"The primary endpoint was progression-free survival (PFS). Secondary endpoints included toxicity, overall survival (OS), objective response rate (ORR), and clinical benefit rate (CBR). Prespecified-protocol enrollment of ≥14 patients with *CTNNB1*$_{mut}$ in the experimental arm was required and *CTNNB1*$_{mut}$ status was correlated with efficacy endpoints."

Lines 54-55
"Among *CTNNB1*$_{mut}$ tumors, there was no difference in CBR (82.4% vs 93.3%, p=0.60) and ORR (58.8% vs 26.7%, p=0.09)."

Lines 61-64
"Although it did not improve survival in all-comers, the addition of ribociclib to everolimus/letrozole may improve efficacy in patients previously treated with endocrine therapy and cases with *CTNNB1*$_{mut}$ tumors were observed to have exceptional response. Future study designs investigating the role of ribociclib should be focused in the *CTNNB1*$_{mut}$ EC tumor population."

Pamela T. Soliman, M.D., M.P.H.
Professor and Ad Interim Chair
psoliman@mdanderson.org

Department of Gynecologic Oncology and Reproductive Medicine
1155 Pressler Street, Unit 1362
Houston, Texas 77030

Methods
Lines 106-107
"Prespecified-protocol enrollment of ≥14 patients with a *CTNNB1* mutation in the experimental arm was required."
Lines 188-196
"To evaluate the effect of *CTNNB1* mutations on treatment response, we prespecified enrollment ≥14 patients with a *CTNNB1* mutation in the experimental arm. All patients underwent somatic tumor NGS testing and tracking for enrollment of patients with *CTNNB1* mutations was reviewed monthly at the scheduled trial team meetings. We used Fisher's exact test (or log-rank test if appropriate) to compare patients with or without *CTNNB1* mutation with respect to CBR, ORR, PFS, and OS. With 14 of patients with *CTNNB1* mutation in the experimental arm, there was 80% power with a one-sided significance level of 0.10 to detect a difference in CBR rates of 0.33 for patients without *CTNNB1* mutation and 0.70 for patients with *CTNNB1* mutation."

Results
Lines 242-243
"Within the experimental arm, those with $CTNNB1_{mut}$ tumors had significantly higher CBR compared to those with $CTNNB1_{WT}$ tumors (82.4% [14/17] vs 46.7% [7/15], p=0.03)."

Lines 252-253
"There was no difference in CBR between arms among $CTNNB1_{mut}$ tumors (93.3% vs 82.4%, p=0.60)."

Discussion
Lines 310-315
"It should be noted that despite this prespecified subgroup analysis of $CTNNB1_{mut}$ tumors, the study was not powered to compare the efficacy of the experimental versus control arms in the $CTNNB1_{mut}$ tumor population. Furthermore, the study was not powered to determine the impact of *CTNNB1* mutation status on treatment efficacy within the control arm. Thus, the study may not have been sufficiently powered to detect a beneficial signal that may be less pronounced. Future studies should be designed to evaluate these regimens in a larger $CTNNB1_{mut}$ only tumor population."

Lines 372-376
"In conclusion, we observed significant, durable antitumor activity of everolimus and letrozole with or without ribociclib in advanced/recurrent endometrioid EC. Despite no observed statistical difference in efficacy between overall treatment arms, the addition of ribociclib to everolimus and letrozole demonstrated exceptional response in tumors harboring *CTNNB1* mutations and should be examined in larger, future trials enriched with this molecular profile."

2. In the section "Impact of CTNNB1 mutation status on efficacy – a prespecified analysis", the authors describe differences in PFS and OS between CTNNB1mut and CTNNB1wt tumors within the experimental and within the control arms. Although these results are interesting, they do not address the main hypothesis stated by the authors that "addition of ribociclib would improve therapeutic efficacy, particularly among patients with CTNNB1 mutant tumors". To address that, the comparison in PFS and OS should occur in CTNNB1mut and CTNNB1wt tumors between arms (as they did with ORR in Lines 234-235), and not within arms. In other words, they should compare PFS and OS between letrozole/ribociclib vs letrozole/ribociclib/everolimus among CTNNB1mut tumors and show these PFS and OS survival curves as Figures in the main results. Based on the supplement Table 6 where these comparisons are made, there is no statistically significant difference in PFS and OS between control and experimental arms among CTNNB1 mutated tumors. These findings need to be reported in the main results and discussed in the discussion section.

Response: Thank you for this comment and suggestion. We have added Figures 3e and 3f that demonstrate K-M curves comparing PFS and OS among $CTNNB1_{mut}$ tumors between treatment arms. We have added lines demonstrating no difference between arms among $CTNNB1_{mut}$ tumors but as discussed earlier, the study was not powered to compare these two arms among $CTNNB1_{mut}$ tumors. Please see the revisions to the text in the following lines:

Results
Lines 251-252
There was no difference in PFS or OS between arms among $CTNNB1_{mut}$ tumors (Figures 3e-f).

Pamela T. Soliman, M.D., M.P.H.
Professor and Ad Interim Chair
psoliman@mdanderson.org

Department of Gynecologic Oncology and Reproductive Medicine
1155 Pressler Street, Unit 1362
Houston, Texas 77030

Discussion
Lines 310-315
"It should be noted that despite this prespecified subgroup analysis of *CTNNB1*$_{mut}$ tumors, the study was not powered to compare the efficacy of the experimental versus control arms in the *CTNNB1*$_{mut}$ tumor population. Furthermore, the study was not powered to determine the impact of *CTNNB1* mutation status on treatment efficacy within the control arm. Thus, the study may not have been sufficiently powered to detect a beneficial signal that may be less pronounced. Future studies should be designed to evaluate these regimens in a larger *CTNNB1*$_{mut}$ only tumor population."

3. Based on points 1 and 2 above, the statement in the abstract conclusions Lines 59-61 that "Although it did not improve survival in all-comers, the addition of ribociclib to everolimus/letrozole may specifically improve survival in CTNNB1mut EC and improve efficacy in patients previously treated with endocrine therapy" is not supported by the data (CBR, PFS and OS comparisons between arms within CTNNB1mut tumors) presented in the study. This statement in the abstract and relevant discussion need to be revised accordingly.

Response: Thank you for this comment. Please see our response/revisions noted for Point 1 above and we have revised the Abstract and Discussion accordingly.

MINOR CONCERNS
1. Abstract, Lines 52-53, "Addition of ribociclib resulted in a higher ORR among those with prior endocrine therapy (61.5% vs 0.0%, p<0.001)": The 0% response rate of letrozole/everolimus among patients who had received prior hormonal therapy is striking and should be discussed further. What hormonal therapies did patients on letrozole/everolimus alone arm receive? In the previous study of everolimus/letrozole/metformin by the same group which allowed prior hormonal therapy and ORR was 28%, were responses seen only among hormonal therapy naive patients? What about the previous letrozole/everolimus study by Slomovitz et al.?

Response: Thank you for comment. We have incorporated the details below into Table 1 under endocrine therapy. Please note that patients may have received more than one line of prior endocrine therapy.

	Safety lead-in	Control Arm	Experimental Arm
Aromatase inhibitor	1	7	6
Progestin plus SRM	3	3	4
Hormonal receptor blocker	2	7	3
Progestin therapy alone	0	1	1

We appreciate the questions regarding other studies performed at our center. Unfortunately, the two study protocols mentioned are closed and the data are not available for access. Analysis and access would require IRB-protocols to be approved and are outside the scope of the study. However, these are interesting questions that should evaluated in a separate study.

2. Abstract, Lines 45-46, "Secondary endpoints included toxicity, OS, ORR": Per study protocol, Section 2.3, clinical benefit was also a secondary endpoint and therefore should be included in the abstract and reported in the results. ORR is not specifically stated as a secondary endpoint in the protocol except only as part of "to determine the presence of a CTNNB1 mutation is associated with response to ribociclib (LEE011), everolimus, and letrozole."

Response: Thank you for this comment. CBR was added to the manuscript abstract. Please see the addition of this information in lines 54-55 of the revised manuscript:

"Among *CTNNB1*$_{mut}$ tumors, there was no difference in CBR (82.4% vs 93.3%, p=0.60) and ORR (58.8% vs 26.7%, p=0.09)."

3. Methods, Line 104, "Prespecified-protocol enrollment of ≥14 patients with a CTNNB1 mutation was required": It needs to be clarified whether ≥ 14 patients were required for each arm versus the entire study. Also, more information needs to be provided on how the investigators kept track of when the required number of CTNNB1mut tumors was achieved.

Pamela T. Soliman, M.D., M.P.H.
Professor and Ad Interim Chair
psoliman@mdanderson.org

Department of Gynecologic Oncology and Reproductive Medicine
1155 Pressler Street, Unit 1362
Houston, Texas 77030

Response: Thank you for this comment. Enrollment of at least 14 patients for the experimental arm was required. All patients got somatic tumor NGS testing and tracking for enrollment of patients with *CTNNB1* mutations was reviewed at the monthly protocol review with the clinical trial team.

Please see the addition of this information in lines 190-192 of the revised manuscript:
"All patients underwent somatic tumor NGS testing and tracking for enrollment of patients with *CTNNB1* mutations was reviewed monthly at the scheduled trial team meetings."

4. Discussion, Lines 291-294: In the discussion of relevant literature on CTNNB1mut tumors, the authors should also include data from a recently published study of letrozole/abemaciclib/metformin in Nature Communications which showed that presence of CTNNB1 mutations correlated with clinical benefit and that 2 of the 3 patients with complete responses to letrozole/abemaciclib/metformin harbored CTNNB1 mutations (PMID: 41339350).

Response: Thank you for this suggestion. We have added this important reference in the manuscript.

Please see lines 322-325 of the revised manuscript:
"Additionally in another single-arm study by Konstantinopoulos et al., $CTNNB1_{mut}$ EC tumors were associated with clinical benefit with the use of abemaciclib, letrozole, and metformin with two of three complete responses from tumors harboring a $CTNNB1_{mut}$ tumor[36]."

Reviewer #2 (Remarks to the Author):

Randomized Phase II of double (everolimus + letrozole) or triple (+ ribociclib) association in recurrent endometrial carcinoma.
2 steps design with a safety lead-in and an open randomized ph 2 of double (everolimus + letrozole) or triple (+ ribociclib) association in recurrent endometrial carcinoma.
The objectives : To characterize the toxicity, the efficacy, and the molecular subtypes that would benefit the most from the triple combination of AI + mTOR inhibitor (everolimus) + CDK4 6 inhibitor (everolimus).
This is an important question in a population with an unmet medical need.

Major remarks :
Randomized phase 2 study conducted in only two centers, which required 5 years to complete enrollment. This is a long period for a common indication (all endometrioid/mixed EC after at least one line of chemotherapy, up to two lines).
Why was the study not expanded to include additional centers?

Response: Thank you for this question. This was an investigator-initiated study that was performed at The University of Texas MD Anderson Cancer Center and Lyndon B. Johnson General Hospital, a safety-net hospital where MD Anderson Gynecologic Oncology Faculty also practice and provide care to underserved populations. This trial occurred during the COVID-19 pandemic which led to a pause in participant enrollment and thus affected the speed of recruitment. When open, we accrued on average 2 patients per month which was our target accrual.

Population
Population is heterogenous: number of prior lines, prior hormonal treatments, histology, hormonal status can impact the response and toxicity; 1 or 2 stratification factor could have been introduced; to be comment in the statistical paragraph and in the discussion in the safety results.

Response: Thank you for raising this point. Although it would be ideal to be able to stratify by 1 or 2 factors, this unfortunately would significantly reduce statistical power to detect differences between groups which is challenging for smaller phase II RCTs. In general, the randomization process should balance the differences in clinicopathologic characteristics and decrease selection bias. Additionally, we had removed p-values to denote any statistically significant differences between treatment arms given many journals discourage use of p-values in Table 1 but there were no statistically significant differences between groups and thus were balanced.

Pamela T. Soliman, M.D., M.P.H.
Professor and Ad Interim Chair
psoliman@mdanderson.org

Department of Gynecologic Oncology and Reproductive Medicine
1155 Pressler Street, Unit 1362
Houston, Texas 77030

Population, in particular molecular status, are imbalanced between the safety run and the ph 2, and could impact results
The rational to include patients whatever the hormonal receptor status for an hormonal treatment association have to be explain.
The definition of ER/PR positivity, the absence of centralisation of the results could impatc the results and should also be mentioned.

Response: Thank you for raising these points. Given the safety lead-in cohort was introduced to determine the recommended phase II dose of the experimental arm, the safety and efficacy of the safety lead-in cohort was reported but not compared to the cohorts in the phase II portion of the study. Thus, molecular characteristics of the safety lead-in cohort should be limited in impact for interpretation of the comparative results.

The definition of ER/PR positivity as the threshold in multiple other studies has varied from at least 1% to at least 10%. Given the target population was selective for endometrioid/mixed endometrioid histology, the prevalence of ER/PR positivity is high (>80-90%), even in poorly differentiated grade 3 endometrioid tumors (50%). Given the high prevalence of ER/PR positivity in endometrioid tumors and the low threshold to be considered hormonal receptor positive, we did not mandate hormonal receptor status as an inclusion criteria since it would serve as another potential barrier for clinical trial enrollment, particularly for our underserved populations. We had defined positivity as any ER/PR positivity (1% or greater) vs no positivity (Supplemental Table 3) and greater than 90% of patients in the randomized phase portion had ER/PR positivity. We also demonstrated the breakdown of percentages per patient in the Heatmap shown in Supplemental Figure 2.
Regarding centralization of results, all p53, MMR, and quantification of ER/PR by IHC was reviewed and evaluated by study pathologists subspecialized in gynecologic cancer pathology.

We have added text to clarify ER/PR status and centralization of results in the revised manuscript. Please see below:

Lines 157-160
"Additionally, immunohistochemistry staining on pre-treatment tumor biopsies were performed for p53, mismatch repair proteins (MMR), and ER/PR; these were all centrally reviewed by two gynecologic oncology study pathologists. A tumor was considered ER/PR positive if there was at least 1% expression)."

Lines 364-371
"Another limitation may be the lack of requirement for ER/PR positivity for trial eligibility criteria and this may have led to a possible decrease in treatment efficacy. The trial inclusion criteria required endometrioid histology which is known to have high prevalence of ER/PR positivity, even in poorly differentiated endometrioid tumors. Thus, the majority (>90%) had ER/PR positivity in the randomized phase portion and the impact of mandating ER/PR stain positivity is likely minimal. Although ER/PR staining is becoming routinely performed in clinical practice for endometrial cancer, it was not routinely performed at trial initiation and may have served as another barrier/delay to trial enrollment, particularly in underserved populations."

In the inclusion criteria, non chemotherapy treatments are not counted as prior lines. Why are ADCs (which are chemotherapy agents) excluded from the count of prior chemotherapy lines? It could impact results on toxicity and efficacy.

Response: Thank you for raising this point. At the design of this trial, ADCs were not being investigated for endometrial cancer and thus were not included as part of prior lines of chemotherapy in the trial protocol. Of note, there were no patients enrolled on the trial with a prior line that included an ADC.

Given the hypothesis of increased efficacy, particularly in the population of CTNNB1 mutated tumors considered from the outset of the study, why was this factor not used for stratification ?

Response: Thank you for raising this point. At the design of this trial, there was not enough data to justify using *CTNNB1* mutation status as a stratification factor that could potentially reduce statistical power that could be observed in the all comer population. Thus, there was a protocol pre-specified enrollment of patients with *CTNNB1* mutations in the experimental arm in order to at least enrich the population for this mutation.

Pamela T. Soliman, M.D., M.P.H.
Professor and Ad Interim Chair
psoliman@mdanderson.org

Department of Gynecologic Oncology and Reproductive Medicine
1155 Pressler Street, Unit 1362
Houston, Texas 77030

Statistics
The authors mention PFS results around 6 months with the everolimus–letrozole combination. Why does the statistical hypothesis start from an estimated 3 month median PFS for the control arm? This may affect the statistical calculation of the required sample size and potentially overestimate the benefit of the investigational arm."

Response: Thank you for this point. The hypothesis and design of this trial was based on the results of a phase II trial of everolimus and letrozole in endometrial cancer which had a median PFS of 3 months and included any endometrial cancer histologic subtype (Slomovitz et al. A randomized phase II study of everolimus and letrozole in patients with recurrent endometrial carcinoma; J Clin Oncol . 2015 Mar 10;33(8):930-6. PMID: 25624430). Given this prior data, the design of the trial had the null hypothesis for control arm at 3 months and had excluded any patients without an endometrioid histologic component given the observed poor response rates. Given the inclusion criteria being restricted to having an endometrioid histologic component, this may have contributed to overperformance of the control arm and there is a potential that this study may be underpowered.

We have stated this in the discussion below in lines 275-280 of the revised manuscript.

"The overperformance of the control arm may have limited the ability to detect a difference between arms at the specified sample size since sample size calculations were based on available PFS trial data reported by Slomovitz et al.[13]. In this study, the trial population was heterogeneous with the patients with serous histology having no treatment responses (0/11 patients) to everolimus and letrozole[13]. Given this finding, pure non-endometrioid tumors were excluded in the current trial design and this may have improved efficacy in the control arm."

Results

Safety lead-in part :
Level 0 is based on safety of associations used in BC. The rational of escalation/desescalation is unclear : the dose -1A level with higer doses of everolimus than the level 0 and level -1 is unclear ; to be explain
The results of this part of the study are not presented /commented in the results section.
It is not clearly stated which dose was selected for the phase 2 study, nor the rationale for this choice

Response: Thank you for this comment. There was evidence of everolimus at 2.5-5 mg in the therapeutic window based on ongoing breast cancer studies using ribcociclib, everolimus, and exemestane (Bardia et al. 2020 [PMID: 32998962] and Bardia et al. 2021 [PMID: 33722897]). The company had recommended this strategy of dose escalation of everolimus in order to maximize dose of everolimus and minimize toxicity because ribociclib was a CYP3A4 inhibitor and everolimus a CYP3A4 substrate.

Regarding the results of the safety lead-in, we had discussed this in the Methods section given it appeared most appropriate for the flow of the manuscript and placed the details of the safety lead-in in Figure 1A. We had omitted to repeat the findings in the text of the results section to avoid redundancy however, we have added a passage regarding the dose selection for better clarification in the revised manuscript.

Lines 200-203
"In the safety lead-in phase, 17 patients were enrolled (9/2017-11/2018) and treated with everolimus, letrozole, and ribociclib. Details of the dose selection for the experimental arm are demonstrated in Figure 1a. There were two DLTs each in the DL 0 and -1A cohorts and thus DL -1 was chosen."

The combination is toxic, with 20% of patients discontinuing ribociclib and 10% discontinuing the tritherapy. The authors should acknowledge this is a limitation of the study. Is that comparable as described is other trials in breast carcinoma ? To be comment/discussed.
Do the authors plan to adjust the doses if they were abble to conduct another study?

Pamela T. Soliman, M.D., M.P.H.
Professor and Ad Interim Chair
psoliman@mdanderson.org

Department of Gynecologic Oncology and Reproductive Medicine
1155 Pressler Street, Unit 1362
Houston, Texas 77030

Response: Thank you for this comment. Although discontinuation rates were numerically higher in the experimental arm, we did not see a statistically significant difference between arms. Although it is challenging to compare across trials (particularly since our study had a heavily pretreated population), treatment discontinuation all drugs in the experimental arm occurred in 3 of 35 (8.6%) of patients which is in line with other CDK4/6 therapeutic trials in endometrial and breast cancer (7-12.5%) (PMID: 36174113, 39657575, 32998962, 33722897). Treatment discontinuation due to adverse events have been in the similar range compared to historical data of drug regimens that have received regulatory approval in less heavily pretreated populations (e.g. first line and second line treatment setting) for endometrial cancer (e.g. KEYNOTE-775 – discontinuation of pembrolizumab/lenvatinib of 33% - PMID 35045221; RUBY trial – discontinuation of dostarlimab of 17% - PMID 36972026).

We do not plan to adjust the doses if another study were to be conducted.

Minor remarks
What were the dose reduction rules in cases of neutropenia?

Response: Thank you for this question. Standard dose reduction rules for neutropenia were used. These rules can be referenced in the supplemental study protocol on page 43.

A recent biopsy or a biopsy performed at inclusion was used for non centralized molecular analysis, which may impact the results of the subgroup analysis.

Response: IHC stains for all cases were centrally reviewed by two study pathologists trained in gynecologic oncology pathology and this is noted in the methodology section. Please see the text in the revised manuscript on lines 157-160.

“Additionally, immunohistochemistry staining on pre-treatment tumor biopsies were performed for p53, mismatch repair proteins (MMR), and ER/PR; these were all centrally reviewed by two gynecologic oncology study pathologists. A tumor was considered ER/PR positive if there was at least 1% expression).”

Define the positivity of HR
Presentation of results : some informations in table 1 are less important than thoses in supplement datas. The results on race and social status may be mentioned in the supplements, whereas the information on previous treatments, HR status and molecular status (p53, MMR, PI3KA, PTEN, POLE) could be included in this table.

Response: HR positivity was defined as at least 1% of ER or PR (please see above response). Given the greater importance on race and social status for clinical trial inclusion, we feel it is important to include these in the paper. Given the exploratory analyses with these molecular subtypes and to avoid a long table 1, we feel it would be more suitable for the supplemental tables.

Reviewer #3 (Remarks to the Author):

I co-reviewed this manuscript with one of the reviewers who provided the listed reports. This is part of the Nature Communications initiative to facilitate training in peer review and to provide appropriate recognition for Early Career Researchers who co-review manuscripts.

Reviewer #4 (Remarks to the Author):

For the last part of the results (lines 245-249) - given the limited sample size, I think the conclusion that the there is a difference in the experimental arm but not control over-states the results. The hazard ratio in the control arm is 0.40 for PFS and 0.44 for OS in the control arm - while not as large as the experimental arm, the HRs are quite far from null. The sample size means the CI has limited precision but I think all we can conclude here is limited precision - I wouldn't say a signal in one but not the other (or rather a signal in one group, but less precision and potentially a less extreme signal in the other).

Pamela T. Soliman, M.D., M.P.H.
Professor and Ad Interim Chair
psoliman@mdanderson.org

Department of Gynecologic Oncology and Reproductive Medicine
1155 Pressler Street, Unit 1362
Houston, Texas 77030

In general, we can't just compare p-values in subgroups and conclude difference if there is a significant p-value in one subgroup but not the other. The formal evaluation of this needs an interaction test. It may be the case there is a significant interaction here... but with this N and the data presented, I think it is hard to conclude there really is no signal in the control group.

Response: Thank you for this comment. We have discussed this limitation in the Discussion section in the revised manuscript.

Lines 310-315.
"It should be noted that despite this prespecified subgroup analysis of *CTNNB1*$_{mut}$ tumors, the study was not powered to compare the efficacy of the experimental versus control arms in the *CTNNB1*$_{mut}$ tumor population. Furthermore, the study was not powered to determine the impact of *CTNNB1* mutation status on treatment efficacy within the control arm. Thus, the study may not have been sufficiently powered to detect a beneficial signal that may be less pronounced. Future studies should be designed to evaluate these regimens in a larger *CTNNB1*$_{mut}$ only tumor population."

Discussion line 279 - median not reached with limited follow-up, decreasing hazards of death over time, and small numbers is less meaningful. Reporting the absolute difference is OS estimates at 2 or 3 years would be more meaningful.

Response: Thank you for this comment. Please see the 3-year and 4-year OS rates in the table below. There was more than 3-fold more patients alive at 3 years with *CTNNB1*$_{mut}$ tumors compared to those with *CTNNB1*$_{wt}$ tumors. This was similarly observed for the 4-year OS rate. There was significant improvement in 3-year OS rate in the experimental arm based on *CTNNB1* mutation status that favored *CTNNB1*$_{mut}$ tumors (71.4% vs 20.0%, p = 0.009). This was similarly observed for 4-year OS rate (54.5% vs 13.3%, p = 0.04). We did not observe a difference in the control arm based on *CTNNB1* mutation status for 3-year and 4-year OS rate. To avoid excess number of supplementary tables, we will report the 3-year and 4-year OS rate for the *CTNNB1*$_{mut}$ and *CTNNB1*$_{wt}$ tumor populations treated in the Experimental arm in the text given the median OS is not reached for the *CTNNB1*$_{mut}$ population.

	Control Arm	Experimental Arm
3-year OS rate		
Overall	37.9% (11/29)	43.3% (13/30)
CTNNB1$_{wt}$	29.4% (5/17)	20.0% (3/15)
CTNNB1$_{mut}$	50.0% (6/12)	71.4% (10/14)
4-year OS rate		
Overall	25.9% (8/27)	29.6% (8/27)
CTNNB1$_{wt}$	18.8% (3/16)	13.3% (2/15)
CTNNB1$_{mut}$	36.4% (4/11)	54.5% (6/11)

We have added the following text in the revised manuscript.

Results
Lines 247-250
"3-year OS rate was significantly higher for those with *CTNNB1*$_{mut}$ tumors within the experimental arm (71.4% [10/14] vs 20.0% [3/15], p = 0.009) compared to those with *CTNNB1*$_{WT}$ tumors. This was similarly observed for the 4-year OS rate (54.5% [6/11] vs 13.3% [2/15], p = 0.04)."

Discussion
Lines 301-304
"Within the experimental arm, there was a near 7-fold improvement in median PFS with median OS not reached in *CTNNB1*$_{mut}$ tumors compared to *CTNNB1*$_{WT}$ tumors. Furthermore, the 3-year and 4-year OS rates were significantly higher among those with *CTNNB1*$_{mut}$ tumors."

Minor, line 281 - this is just a report for one patient? So not a median per se but really just the PFS length of that one patient.

Pamela T. Soliman, M.D., M.P.H.
Professor and Ad Interim Chair
psoliman@mdanderson.org

Department of Gynecologic Oncology and Reproductive Medicine
1155 Pressler Street, Unit 1362
Houston, Texas 77030

Response: Thank you for this comment. That is a typo and has been removed. Please see the changes in the revised manuscript.

Lines 304-306
"Notably within the safety lead-in arm, one patient with a CTNNB1 mutation had stable disease without disease progression and had a PFS of 60.4 months prior to death unrelated to disease (urosepsis)."

Pamela T. Soliman, M.D., M.P.H.
Professor and Ad Interim Chair
psoliman@mdanderson.org

Department of Gynecologic Oncology and Reproductive Medicine
1155 Pressler Street, Unit 1362
Houston, Texas 77030

Point-by-point responses to reviewers' comments (Responses in blue)

Reviewer #1 (Remarks to the Author):

The authors have adequately addressed my previous comments. My only remaining concern regarding the revised manuscript pertains to the final statement in the abstract conclusions: "Future study designs investigating the role of ribociclib should be focused in the CTNNB1mut EC tumor population." This conclusion appears overly strong and should, in my view, be removed.
Although cases with CTNNB1-mutant tumors were observed to demonstrate exceptional responses, this does not establish that only patients with CTNNB1-mutant tumors responded to ribociclib. Moreover, while CTNNB1-mutated tumors in the experimental arm (letrozole/everolimus/ribociclib) performed significantly better than CTNNB1 wild-type tumors, this does not necessarily indicate that the observed effect was attributable specifically to ribociclib. Prior studies have shown responses to everolimus in CTNNB1-mutated tumors, suggesting that the exceptional outcomes in this subgroup may be related to the combination of everolimus and ribociclib, rather than to ribociclib alone.

Response: Thank you for this comment. We appreciate the perception of this statement and have removed it from the abstract conclusion. In the manuscript discussion, we have verified that language related to outcomes related to adding ribociclib was in the context of the addition to ribocilib to the combination of everolimus and letrozole.

Reviewer #2 (Remarks to the Author):

Tha authors respond to questions and remarks

Reviewer #3 (Remarks to the Author):

I co-reviewed this manuscript with one of the reviewers who provided the listed reports. This is part of the Nature Communications initiative to facilitate training in peer review and to provide appropriate recognition for Early Career Researchers who co-review manuscripts.

Reviewer #4 (Remarks to the Author):

Lines 256-259: It does not appear that these p-values account for censoring. Kaplan Meier estimates should be reported with Z-tests using Kaplan-Meier variances/standard deviations.

Response: Thank you for this comment. We have calculated KM rate estimates with Z-tests using KM variances and standard/deviations (please see below).

3- year OS

**Statistic**	**KM rate (Greenwood SE)**
CTNNB1 mutant	76.5% (SE 10.3%)
CTNNB1 wild-type	21.4% (SE 10.8%)
Difference	55.0% (SE 14.9%)
Z score	3.694
p-value (two-sided)	0.0002

4-year OS

**Statistic**	**KM rate (Greenwood SE)**
CTNNB1 mutant	68.0% (SE 12.2%)

Pamela T. Soliman, M.D., M.P.H.
Professor and Ad Interim Chair
psoliman@mdanderson.org

Department of Gynecologic Oncology and Reproductive Medicine
1155 Pressler Street, Unit 1362
Houston, Texas 77030

CTNNB1 wild-type	14.3% (SE 9.3%)
Difference	53.7% (SE 15.3%)
Z score	3.514
p-value (two-sided)	0.0004

We have revised the manuscript on lines 246-250.

"The 3-year Kaplan-Meier OS rate estimate was significantly higher for those with $\textit{CTNNB1}_{mut}$ tumors within the experimental arm (76.5% [standard error (SE) 10.3%] vs 21.4% [SE 10.8%], p = 0.0002; Z score 3.7) compared to those with $\textit{CTNNB1}_{WT}$ tumors. This was similarly observed for the 4-year Kaplan-Meier OS rate estimate (68.0% [SE 12.2%] vs 14.3% [SE 9.3%], p = 0.0004; Z score 3.5)."

Line 260 references results in the control arm comparing CTNNB1 mut vs wt, but I don't see an equivalent of 3c and 3d for the control arm.

Relatedly, Sup figure 3e and 3f show much less of a gap between curves (with limited n) then observed in Sup figure 3c and 3d. It is important to understand how much of the results in Sup figure 3c and 3d are due to the prognostic association of CTNNB1. Additional curves with the control arm would be important.

Response: Thank you for these comments. Regarding PFS and OS for the control arm by *CTNNB1* status (mutant vs wildtype), we have these K-M curves shown and referenced in Supplemental figure 3a and 3b. We did not include these in the main figures in order to limit total number of figures. We did not observe a statistically significant difference based on *CTNNB1* status for median PFS (8.2 vs 13.8 months, p=0.08) and OS (39.7 vs 13.3 months, p=0.12). However, this study was not powered to determine benefit of everolimus plus letrozole in the control arm and we have stated this in the discussion section (manuscript lines 311-321).

Regarding prognostic significance of *CTNNB1* mutation status, *CTNNB1* mutant tumors has been associated with poorer prognosis and are more clinically aggressive compared to *CTNNB1* wildtype tumors in the literature (PMID 25214561, 33207095, 35483739, 28281553). Furthermore in prospective biomarker-driven trials (e.g. PORTEC-4A), trials have designated *CTNNB1* wildtype tumors in the favorable category whereas *CTNNB1* mutant tumors are designated in the less favorable category (PMID 41449145).

We have stated in the discussion that there is some evidence for efficacy in everolimus in *CTNNB1* mutant tumors in a prior study by Slomovitz et al.

Please see lines 316-321 in the revised manuscript.

"In the aforementioned phase II study by Slomovitz et al., all patients (4/4) with $\textit{CTNNB1}_{mut}$ endometrioid EC had response to everolimus/letrozole[13]. Thus, the study may not have been sufficiently powered to detect a beneficial signal that may be less pronounced within the control arm and between arms among $\textit{CTNNB1}_{mut}$ tumors. Future studies should be designed to evaluate these regimens in a larger $\textit{CTNNB1}_{mut}$ only tumor population."

Lines 314-316 over-state the conclusions. Finding significant p-values in one group and non-signficant in another group do not by themselves do not "support to the clinical benefit for ribociclib in CTNNB1mut tumors" (line 309-310).

Response: Thank you for this comment. In the prior revisions, we had included text stating the inconclusive results comparing efficacy among *CTNNB1* mutant tumors between treatment arms and based on *CTNNB1* mutation status within the control arm. We have made additional revisions in the manuscript to reduce overstating the conclusions:

Lines 283-285

Pamela T. Soliman, M.D., M.P.H.
Professor and Ad Interim Chair
psoliman@mdanderson.org

Department of Gynecologic Oncology and Reproductive Medicine
1155 Pressler Street, Unit 1362
Houston, Texas 77030

"With respect to our a priori hypothesis examining the benefit of adding ribociclib to everolimus/letrozole in *CTNNB1*$_{mut}$ tumors, there were a number of patients with *CTNNB1*$_{mut}$ tumors who had exceptional PFS and OS outcomes within the experimental arm."

Lines 311-321

"It should be noted that despite this prespecified subgroup analysis of *CTNNB1*$_{mut}$ tumors, the study was not powered to compare the efficacy of the experimental versus control arms in the *CTNNB1*$_{mut}$ tumor population. Furthermore, the study was not powered to determine the impact of *CTNNB1* mutation status on treatment efficacy within the control arm. Although there was no statistically significant difference within the control arm based on CTNNB1 status, there was trend towards better PFS and OS in among *CTNNB1*$_{mut}$ tumors (Supplementary Figure 3a-b). In the aforementioned phase II study by Slomovitz et al., all patients (4/4) with *CTNNB1*$_{mut}$ endometrioid EC had response to everolimus/letrozole[13]. Thus, the study may not have been sufficiently powered to detect a beneficial signal that may be less pronounced within the control arm and between arms among *CTNNB1*$_{mut}$ tumors. Future studies should be designed to evaluate these regimens in a larger *CTNNB1*$_{mut}$ only tumor population."

Figure 4 needs to include all patients (those without tumor measurements should be annotated in some way as no responding). It does not appear to include all patients.

Response: Thank you for this comment. We have annotated the figure to state that there were 4 patients who were not evaluable for anti-tumor activity by RECIST.

Please see the revised legend on lines 600-602 of the revised manuscript:

"Legend: Horizontal lines at -30% and 20% denote partial response and disease progression by RECIST v1.1 criteria, respectively. All four patients with a complete response (three from experimental arm and one from control arm) did not have any evidence of disease recurrence at the time of data cutoff. Three patients in the safety lead-in and one patient in the experimental group were not evaluable for antitumor response and therefore are not included in the waterfall plot."

Pamela T. Soliman, M.D., M.P.H.
Professor and Ad Interim Chair
psoliman@mdanderson.org

Department of Gynecologic Oncology and Reproductive Medicine
1155 Pressler Street, Unit 1362
Houston, Texas 77030

Point-by-point responses to reviewers' comments (Responses in blue)

Reviewer #4 (Remarks to the Author):

Given the sample size, those interaction p-values are rather compelling. It could be useful to provide them somewhere in the manuscript as a reference for any future research, but this is just a thought and not a requirement.

Response: Thank you for this comment and suggestion. Interaction tests generally have significantly lower statistical power than evaluating for the main effects but will often require much larger sample sizes. As a result, despite the observed values, the current study may be underpowered to reliably assess for an interaction/moderation effect. We have stated that this is a limitation in the study and should be evaluated in future studies in the revised manuscript in lines 183-193.

"It should be noted that despite this prespecified subgroup analysis of *CTNNB1*$_{mut}$ tumors, the study was not powered to compare the efficacy of the experimental versus control arms in the *CTNNB1*$_{mut}$ tumor population. Furthermore, the study was not powered to determine the impact of *CTNNB1* mutation status on treatment efficacy within the control arm. Although there was no statistically significant difference within the control arm based on *CTNNB1* status, there was trend towards better PFS and OS in among *CTNNB1*$_{mut}$ tumors (Supplementary Figure 3a-b). In the aforementioned phase II study by Slomovitz et al., all patients (4/4) with *CTNNB1*$_{mut}$ endometrioid EC had response to everolimus/letrozole[13]. Thus, the study may not have been sufficiently powered to detect a beneficial signal that may be less pronounced within the control arm and between arms among *CTNNB1*$_{mut}$ tumors. Future studies should be designed to evaluate these regimens in a larger *CTNNB1*$_{mut}$ tumor population and further evaluate the interaction of *CTNNB1*$_{mut}$ status (e.g. effect modification) on treatment response."
